# Phase 1, randomized, rater and participant blinded placebo-controlled study of the safety, reactogenicity, tolerability and immunogenicity of H1N1 influenza vaccine delivered by VX-103 (a MIMIX microneedle patch [MAP] system) in healthy adults

**DOI:** 10.1371/journal.pone.0303450

**Published:** 2024-06-06

**Authors:** Naveen Garg, Guy Tellier, Noah Vale, Jon Kluge, Jonathan L. Portman, Anna Markowska, Lynda Tussey

**Affiliations:** 1 Centricity Research-Montreal, Point-Claire, Québec, Canada; 2 Centricity Research-Mirabel, Mirabel, Québec, Canada; 3 Centricity Research-Toronto, Toronto, Ontario, Canada; 4 Research and Development, Vaxess Technologies, Cambridge, Massachusetts, United States of America; 5 Development and MAP Production, Vaxess Technologies, Woburn, Massachusetts, United States of America; IAVI, UNITED STATES

## Abstract

**Background:**

The MIMIX platform is a novel microneedle array patch (MAP) characterized by slowly dissolving microneedle tips that deploy into the dermis following patch application. We describe safety, reactogenicity, tolerability and immunogenicity for MIMIX MAP vaccination against influenza.

**Methodology:**

The trial was a Phase 1, exploratory, first-in-human, parallel randomized, rater, participant, study analyst-blinded, placebo-controlled study in Canada. Forty-five healthy participants (18 to 39 years of age, inclusive) were randomized in a 1:1:1 ratio to receive either 15 μg or 7.5 μg of an H1N1 influenza vaccine, or placebo delivered via MIMIX MAP to the volar forearm. A statistician used a computer program to create a randomization scheme with a block size of 3. Post-treatment follow-up was approximately 180 days. Primary safety outcomes included the incidence of study product related serious adverse events and unsolicited events within 180 days, solicited application site and systemic reactogenicity through 7 days after administration and solicited application site erythema and/or pigmentation 14, 28, 56 and 180 days after administration. Immunogenicity outcomes included antibody titers and percentage of seroconversion (SCR) and seroprotection (SPR) rates determined by the hemagglutination inhibition (HAI) assay. Exploratory outcomes included virus microneutralization (MN) titers, durability and breadth of the immune response. The trial was registered with ClinicalTrials.gov, number NCT 06125717.

**Findings:**

Between July 7, 2022 and March 13, 2023 45 participants were randomized to a treatment group. One participant was lost to follow up in the 15 μg group and 1 participant withdrew from the 7.5 μg dose group. Safety analyses included n = 15 per group, immunogenicity analyses included n = 14 for the 15 μg and 7.5 μg treatment groups and n = 15 for the placebo group. No SAEs were reported in any of the treatment groups. All treatment groups reported solicited local events within 7 days after vaccination, with mild (Grade 1) erythema being the most frequent symptom reported. Other local symptoms reported included mostly mild (Grade 1) induration/swelling, itching, pigmentation, skin flaking, and tenderness. Within 7 days after vaccination, 2 participants (4.4%) reported moderate (Grade 2) erythema, 1 participant (2.2%) reported moderate (Grade 2) induration/swelling, and 1 participant (2.2%) reported moderate (Grade 2) itching. There was an overall reduction in erythema and pigmentation reported on Days 15, 29, 57, and 180 among all treatment groups.

Systemic symptoms reported within 7 days after vaccination, included mild (Grade 1) fatigue reported among all treatment groups, and mild (Grade 1) headache reported by 1 participant in the 7.5 μg treatment group.

No study drug related severe symptoms were reported in the study.

Group mean fold rises in HAI titers ranged between 8.7 and 12-fold, SCRs were >76% and SPRs were >92% for both VX-103 dose groups thereby fulfilling serological criteria established by the EMA and FDA for seasonal influenza vaccines. Longitudinal assessments demonstrate persistence of the immune response through at least Day 180.

**Conclusions:**

The MIMIX MAP platform is safe, well tolerated and elicits robust antibody responses.

## Introduction

Although influenza vaccines reduce the risk of illness by between 40% and 60% among the overall population, factors such as age and the degree of antigenic match are known to influence their effectiveness [1–4]. One factor believed to affect influenza vaccine effectiveness is waning immunity within the influenza season, particularly for older subjects [5]. A recent study has suggested that the waning immunity may be linked to the rapid decline of bone marrow plasma cells (which correlate with circulating levels of antibody secreting cells) that are responsible for the long-term maintenance of serum antibody levels [6]. Thus, there is a current need for vaccine platforms that can improve vaccine effectiveness and extend the durability of the immune response.

There has been a long-standing interest in intradermal (ID) influenza vaccines because of their demonstrated potential for dose-sparing and improvement of immune responses [7–10]. Microneedle array patches, or MAPs, are a more recent minimally invasive alternative to needle and syringe for ID delivery. MAPs comprise an array of microprojections attached to a backing that can be applied to the skin with finger pressure or an applicator. They generally are categorized into four main types, hollow, solid/coated, swellable and dissolving with much of the focus being on dissolving and coated microneedle platforms [11–19]. MAPs have a number of potential logistical advantages including reduced costs for transport and distribution (due to their thermostability), easier and safer administration (no need for reconstitution), and greater acceptability and potentially less hesitancy by end-users due to the perception of being less painful, as they are needle-free [15,20–23]. Importantly, they also have the potential for enhanced vaccine immunogenicity, increased breadth of the immune response and dose-sparing effects [24,25] because they efficiently target professional antigen presenting cells (APCs) in the skin [26,27].

The MIMIX MAP is a novel type of dissolving MAP whereby the microneedle tips comprise vaccine antigen and silk fibroin (a stabilizing excipient) to support sustained release of vaccine antigen in the skin. Immunization strategies that support extended antigen availability can result in stronger immune responses [8,28,29], that are associated with larger germinal centers (GCs) and more GC T follicular help (Tfh) cells [30–32] than conventional bolus injections. In mouse models of MIMIX vaccination we have observed sustained stimulation of germinal center responses, trends towards an improvement in the magnitude of HAI titers particularly at timepoints more than 1 month post dosing, a broadening in immunity against drifted influenza strains, and stronger antigen-specific T cell responses than observed with bolus intramuscular (IM) delivery [33].

Use of a water-soluble pedestal, or base, for each microneedle is a second distinguishing feature of the MIMIX MAP that allows for deployment of the array’s tips following application to the skin. Within minutes of placing the patch on the skin, the moisture of the skin dissolves the base, thereby delivering the array of tips to the dermis. Since silk fibroin is resorbable, these tips gradually dissolve and release their payload over time.

The MIMIX MAP therefore draws from two fundamental immunological concepts for vaccines, targeted delivery to professional APC in the skin and sustained supply of antigen, to generate robust and durable immune responses. These attributes have the potential to address some of the shortcomings of current licensed influenza vaccines. This study describes a first in human, Phase 1 feasibility study of MIMIX FLU (code name VX-103) in which the influenza A virus A/Guangdong-Maonan/ SWL1536/2019CNIC-1909 (H1N1) vaccine is delivered intradermally by MIMIX MAP. We hypothesize that as a vaccination strategy, MIMIX MAP delivery will lead to robust durable immune with the potential for dose sparing.

## Materials and methods

### Study design

We conducted a Phase 1, parallel randomized, rater, participant, and study analyst-blinded, placebo-controlled study at 3 Centricity Clinical Research sites in Canada (Montreal, Mirabel and Toronto, CA). The primary objective was to describe the safety, reactogenicity, and tolerability profiles of VX-103 for a fractional (7.5 μg) and a standard (15 μg) H1N1 vaccine dose level versus placebo. Secondary objectives were to assess the immunogenicity of VX-103 based on geometric mean HAI titers and the dose-sparing potential of VX-103 immunization by comparing the safety, reactogenicity, tolerability and immunogenicity profiles for each dose level. As exploratory objectives, the durability and breadth of the immune response, virus microneutralization antibody titers, and HA IgG against the vaccine strain were also assessed. A planned interim analysis of accumulated safety and immunogenicity data was carried out after all participants had completed their Day 57 visits.

VX-103 Clinical Protocol Version 4 (**S1 Text**) was approved by Advarra Institutional Review Board (Approval Number SSU0018857; Ontario, Canada). Written informed consent was obtained from all participants. The study was conducted under the regulatory oversight of Health Canada (Dossier ID HC6-024-c258401, Control #264261) in conformance with the International Council for Harmonization Tripartite Guidelines for Good Clinical Practice, with applicable local regulations and with the ethical principles defined in the Declaration of Helsinki. The trial was registered with ClinicalTrials.gov, number NCT06125717.

This study is reported per the Consolidated Standards of Reporting Trials (CONSORT) guidelines (**S1 Checklist**).

### Participants

Healthy adults aged 18–39 years old, inclusive, with a body mass index of 18 to 35 kg/m^2^, inclusive, were eligible. Men and women of procreative potential were required to practice a highly effective method of contraception through 60 days post-vaccination. Women of childbearing potential were required to have a negative urine pregnancy test prior to vaccination. Those with confirmed or suspected immunosuppression or immunodeficiency, history of autoimmune disease impaired immune responsiveness; experienced Coronavirus-19 within the prior 60 days; or experienced influenza or received an influenza vaccine within the prior 24 months were not eligible.

### Randomization and masking

Forty-five participants were randomized in a 1:1:1 ratio to receive either 15 μg or 7.5 μg of the H1N1 influenza vaccine, or placebo (no vaccine) delivered via the MIMIX MAP to the volar forearm. A validated SAS^®^ program was used to create a distributed, block randomization scheme that generated 90 blocks, with a block size of 3. Each block comprised 3 treatment ID numbers associated with the 3 treatment descriptions. Blocks were apportioned to the 3 clinical sites, some blocks remained unused once the study was fully enrolled. A password protected randomization list providing the treatment assignments within each block was created by the statistician and provided to the clinical sites. All study personnel were unblinded, with the exception of the rater, the participant and the study analysts.

### Vaccine administration

Study treatment was prepared by an unblinded local pharmacist or designee at the study site. The MIMIX MAP was administered to the skin on the volar forearm (specifically, ~4 and 8 cm below the elbow joint) on Day 1 by a trained, blinded study team member. Application sites were free from any blemishes that might have interfered with the detection of local reactions. If possible, the non-dominant arm was used for administration of treatment. The patch remained on the skin for a minimum of 5 minutes and no longer than 10 minutes.

### Vaccine

The monovalent bulk drug substance (GC H1N1, GC3110A) for influenza strain A/ Guangdong-Maonan/SWL 1536/2019-like (H1N1) was sourced from GC Biopharma Corp (Hwasun-eup, Hwasun-gun, Jeollanam-do, Korea). The strain was the A/H1N1 component recommended for the 2020–2021 northern hemisphere (NH) influenza season and was included in GC Biopharma Corp’s influenza vaccine GCFLU. It is a split, inactivated influenza vaccine grown in embryonated healthy flock (non-SPF) eggs. Monovalent bulk was aseptically aliquoted by Berkshire Sterile Manufacturing (Lee, MA) and tested at Vaxess Technologies Inc. (Woburn, MA) and Boston Analytical (Salem, NH) for Vaxess acceptance. The single radial immunodiffusion (SRID) assay was used to confirm the initial concentration of the HA antigen using sera and reference antigens procured from the National Institute for Biological Standards and Control (NIBSC; Potters Bar, Hertfordshire, UK).

### MAP print solutions

Aliquoted monovalent bulk was prepared at Vaxess Technologies (Woburn, MA) for MAP printing by reducing the detergent concentration, concentrating the HA and then formulating the Tip Print Solution to contain 6.2 mcg/mL of HA antigen, 1% silk fibroin (as a novel excipient) and 0.5% Tween-20. The VaxArray assay (InDevR, Boulder, CO) specific for the HA subtype was used to quantify concentrated HA using GC H1N1 DS as a reference. The GC H1N1 concentration value measured at receipt of the bulk by SRID and the concentrated HA values measured by VaxArray are linked through the GC H1N1 DS reference. Tip Print Solution for placebo MAPs contained excipients with no antigen. The Base Formulation used to form the pedestal of each microneedle is a viscous solution comprising 60%wt polyvinylpyrrolidone (PVP) and 0.1%wt Triton X-100.

### MAP printing process

The printing process at Vaxess Technologies (Woburn, MA) uses a silicone mold (Elkem Silbione 4350, Liquid Silicone Rubber) that defines the shape of the microneedle array. The mold is approximately 2.5 cm by 2.5 cm square and contains an 11x11 array of evenly spaced microprojection shaped cavities. Each cavity defines a 1mm long cone with a 30-degree included angle. Molds are packaged inside two layers and are subjected to dry heat (200°C for 4 hr) before use to reduce bioburden.

To form each microneedle tip, Print Solution containing the vaccine (or not for the placebo) was dispensed to partially fill each microprojection shaped cavity of the mold via machine guided nanoliter dispensing (Nordson Picopulse). Print Solution was applied to every microprojection cavity for the high dose and placebo MAP, 121 cavities. Print solution was applied to 60 cavities for the low dose MAP.

Tips were dried in situ under defined humidity condition to form the solid Tip. To form the pedestal or base of each microneedle, Base Formulation was dispensed to each cavity using the machine guided dispensing system. After drying the base under defined humidity conditions, the adhesive side of a bilayer backing (3M Medical Tape 1509 and a non-adhesive structural support (the Plastic Film) made of PET) was applied to form the microarray.

### The MAP delivery system

MAPs were packaged in a single-use, disposable MAP Cartridge. For application, each MAP Cartridge was removed from its packaging and mounted to a single use spring-loaded Applicator. MAPs were deployed onto the skin by using the Applicator to push (deploy) the MAP onto the participant’s skin. The Cartridge was removed from the skin along with the Applicator after MAP deployment. Within five minutes post deployment the MAP Base dissolved, leaving the Tips in the skin to support prolonged release of the antigen. The MAP Backing was removed at the end of the five-minute period.

### Procedures

Participants received study vaccine or placebo on Day 1 and were followed through 6 months thereafter. Safety, reactogenicity, and tolerability were assessed based on physical examinations, vital signs, clinical laboratory results (hematology, chemistry, coagulation, and urinalysis), photographs of the treatment site, local reactogenicity and tolerability assessments, and documentation of adverse events (AEs), and serious adverse events (SAEs). Physical examinations were carried out on Study Days 1 (pre-dose), 2, 4, 8, 15, 29, 57, 85, 119, 180. Safety laboratory tests were carried out on Study Days 1 (pre-dose), 4, 29 and 180. All local and systemic reactogenicity reporting in the 7 days after immunization were supported via in-person clinic visits on Study Days 2, 4 and 8 and by telephone on Study Days 3, 5 and 6. Additional reporting of erythema and coloration were supported by in-person clinic visits on Days 15, 29, 57, 85, and 180.

Hemagglutinin inhibition (HAI) titers were assessed on Study Days 1 (pre-dose), 29, 57, 85, 119 and 180. Virus microneutralization (MN) titers, HAI titers against the antigenically drifted strain and anti-HA IgG were assessed on Study Days 1 (pre-dose), 29 and 180.

### Hemagglutination inhibition (HAI) titers

The HAI assay was used to measure antibodies to the vaccine influenza strain A/Guangdong-Maonan/ SWL1536/2019 CNIC-1909 (H1N1) and an antigenically drifted influenza strain A/Victoria/2570/2019 IIVR-215 (H1N1) in human sera from participants in the study. Testing was performed using validated assays at Q2 Solutions, Vaccines (Durham, NC). Briefly, serum samples were treated with receptor destroying enzyme (RDE; Accurate Chemical) overnight, diluted to 1:10, and serial diluted 2-fold in triplicate from 1:10 to 1:10240. After addition of an equal volume of standardized virus (4 HA / 25 μL), neutralization was performed for 1 hour at 37°C, followed by addition of turkey red blood cells (RBCs). Plates were tilted and the titer determined as the reciprocal of the last dilution that fully inhibits hemagglutination as compared to an RBC control well. Each serum sample was tested in triplicate within the same assay. The three titer results were reported as the geometric mean titer (GMT) for the triplicate. Titers were summarized as geometric mean titers, percentage of seroprotection, and percentage of seroconversion.

### Virus microneutralization (MN) titers

MN assays were conducted by Vaxess Technologies (Cambridge. MA) on serum samples collected on Days 1, 29 and 180. The assay was qualified for use against the A/Guangdong-Maonan/SWL1536/2019 CNIC-1909 strain. Briefly, samples were heat-inactivated at 56°C for 30 minutes and stored at -20°C until use. Sera were serially diluted 2-fold starting from a 1:10 dilution in Dulbecco’s modified Eagle medium with bovine serum albumin and 200 TCID50 (median tissue culture infectious doses) were added to each test. Plates were incubated at 37°C for 60 minutes. After incubation, Madin-Darby canine kidney cells were added at 1.5 x 10^4^ cells/well and the plates were further incubated for 18 to 20 h at 37°C in 5% CO2. Cells were fixed with 80% acetone for 15 minutes at room temperature. After incubation, plates were washed with wash buffer and incubated with goat anti-mouse IgG HRP (SeraCare, Cat No. 5220–0339; 1:8000 in PBS, 0.05% tween, 5% milk) for 1hr at RT. Following incubation with HRP colorimetric substrate (0.5mg/mL *o*-phenylenediamine dihydrochloride, 0.05M phosphate citrate buffer, 0.03% sodium perborate, pH 5.0) signal was detected at 490nm after addition of stop solution (0.5M sulfuric acid) with a Biotek Synergy H1 spectrophotometer. The MN titer for each sample was defined as the reciprocal of the highest serum dilution with an optical density below or equal to cutoff value, which was determined as a mean of the sums of the medians of positive and negative control wells. Geometric means for each sample were calculated from three replicates.

### Outcomes

This was a descriptive study with no formal statistical hypothesis testing. However, a group size of n = 15 has 79% probability of observing an AE with a true event rate of 10%. Solicited AEs were events that participants were specifically asked about and recorded. Unsolicited AEs were spontaneously reported and recorded events.

Safety endpoints included the number and percentage of participants with solicited local and systemic AEs within 7 days post-vaccination, by severity. The percentage of participants reporting injection site erythema and discoloration were also evaluated on Days 29, 57, 85, and 180. Unsolicited AEs were collected at any time. Solicited local symptoms included arm pain, tenderness, erythema, swelling, bruising, itching, skin flaking, and skin coloration. Systemic symptoms including fever, nausea/vomiting, diarrhea, headache, fatigue, and myalgia.

Adverse events were categorized as mild or Grade 1 (aware of symptom but well tolerated), moderate or Grade 2 (hindering enough to interfere with normal daily activity), and severe or Grade 3 (preventing normal daily activity) based on Food and Drug Administration toxicity grading [34]. Continuous results for erythema and swelling (in centimeters) were converted to categorical severity grades as follows: < 1 cm as none, 1 to 5 cm as mild (Grade 1), 5.1 to 10 cm as moderate (Grade 2), and > 10 cm as severe (Grade 3). Continuous results for bruising (in centimeters) were converted to categorical severity grades as follows: none visible as none, ≤ 2 cm as mild (Grade 1), 2 to 5 cm as moderate (Grade 2), and > 5 cm as severe (Grade 3). Continuous results for skin coloration (in centimeters) were converted to categorical severity grades as follows: < 1 cm as none, ≥ 1 cm slightly visible as mild (Grade 1), ≥ 1 cm and noticeable as moderate (Grade 2), and ≥ 1 cm and clearly defined as severe (Grade 3).

Immunogenicity endpoints included geometric mean titer (GMT), geometric mean ratio (GMR), geometric mean fold-rise (GMFR), seroconversion rate (SCR), and seroprotection rate (SPR). As exploratory immunogenicity endpoints, GM MN and HA IgG titers against the vaccine strain and GM HAI titers against an antigenically drifted H1N1 influenza strain were reported for each treatment group on Study Days 1 (pre-vaccination), 29 and 180 for all participants.

### Analysis sets

The randomized set comprised all participants randomized in the study and was used for the disposition summary. The safety analysis set included all participants who received study treatment. This population was used for demographic, baseline characteristic, and safety analyses. The immunogenicity analysis set included participants who received their assigned treatment and completed at least 1 immunogenicity assessment beyond the Day 1 assessment and was used for analysis of immunogenicity endpoints.

### Statistical analyses

The AE summaries included treatment-emergent adverse events (TEAEs), which occurred or worsened after the start of study vaccination. A causally related AE was defined as any AE that was assessed by the investigator as having a relationship to treatment. All AEs were coded by system organ class (SOC) and preferred term (PT) according to Medical Dictionary for Regulatory Activities (MedDRA) Version 25.0. The overall incidence of TEAEs (number and percentage of participants) were summarized by treatment. It included TEAEs, severity of TEAEs in relation to treatment of TEAEs, TEAEs leading to study discontinuation, SAEs, and SAEs resulting in death.

All solicited systemic and local symptoms were summarized through Day 8. Other AEs assessed as possibly, probably, or definitely related to vaccination were collected through Day 180 and summarized. The proportion of participants in each treatment group with any non-zero report were tabulated for each local and systemic solicited and unsolicited reactogenicity event. The proportion of participants with reactogenicity events was compared between dose levels and between each dose level and the placebo using Fisher’s exact test.

Calculated titer values were summarized using descriptive statistics: n, mean percent coefficient of variation (CV%), geometric mean (GM), geometric mean CV%, standard deviation (SD), minimum, median, maximum, and 95% confidence interval (CI). All derived immunogenicity secondary endpoint parameters, including geometric mean titer (GMT), geometric mean ratio (GMR), geometric mean fold increase (GMFI), seroconversion rate (SCR), and (SPR) were summarized by scheduled timepoint and treatment group. SCR and 95% CI was defined as the percentage of participants with either a pre-vaccination HAI titer < 1:10 and post-vaccination HAI titer of ≥ 1:40 or a pre-vaccination HAI titer of ≥ 1:10 and a minimum 4-fold rise in post-vaccination HAI antibody titers. SPR and 95% CI defined as the proportion of participants with HAI titer ≥ 1:40.

## Results

### Disposition and demographics

**Fig 1:**
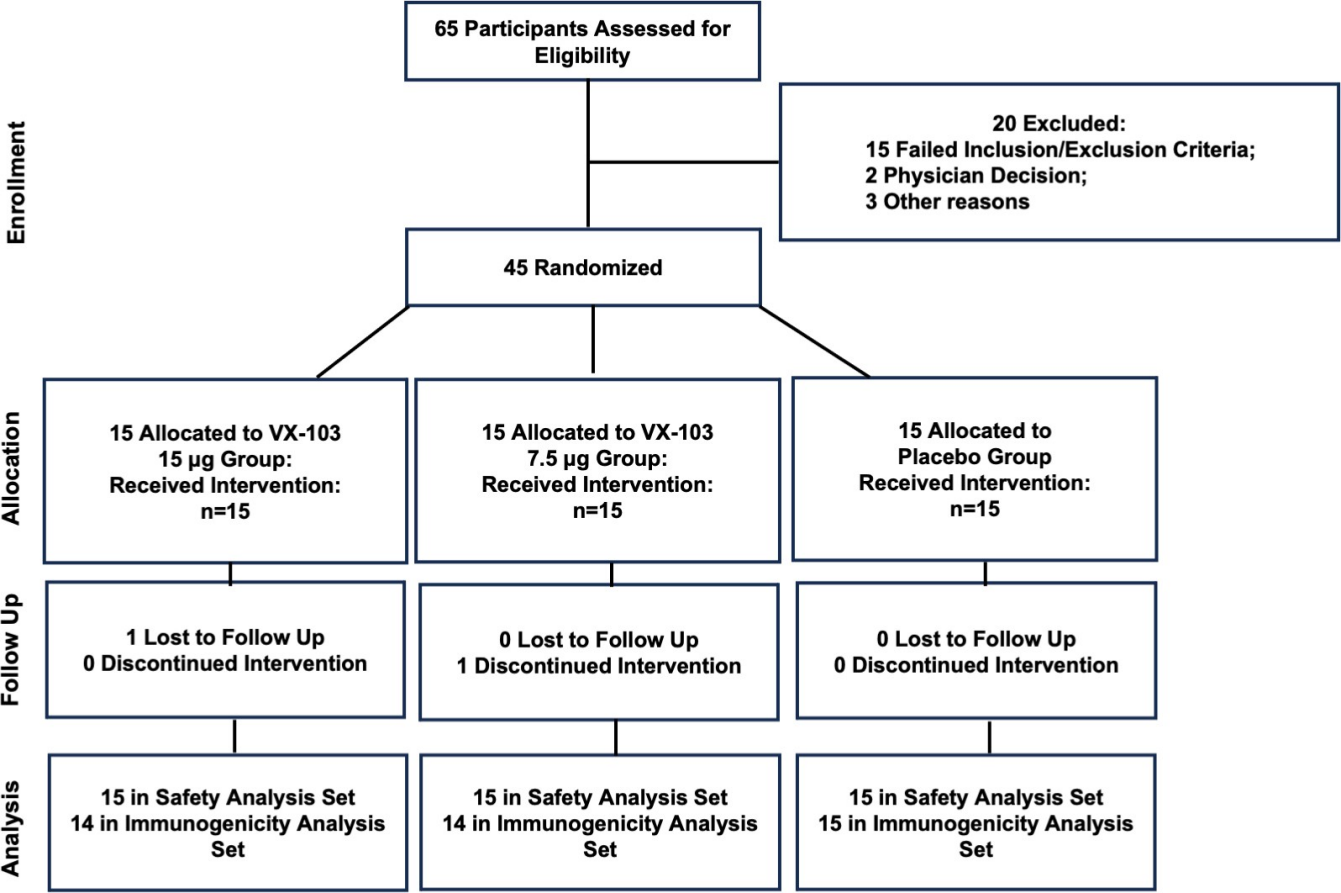
Trial profile. Randomization and flow of participants in the study.

The study was conducted from 07 July 2022 to 13 March 2023 at 3 Canadian Centricity clinical sites. Sixty-five participants were screened, 20 were screen failures, 45 were enrolled as planned, with 15 in each treatment group. Forty-three participants completed the study, with 1 participant in the 15 μg group being lost to follow-up at Study Day 70 and another participant in the 7.5 μg group) electing to withdraw at Study Day 30 (**Fig 1**).

Overall, most (29; 64%) participants were male and white (35; 78%). The mean age of participants was 26.6 years (range 18, 39). Mean height, weight, and body mass index were 173.1 cm, 75.3 kg, and 25.0 kg/m^2^, respectively (**Table 1**).

**Table 1:** Demographic and baseline characteristics (safety analysis set).

	VX-103 15 μg (N = 15)	VX-103 7.5 μg (N = 15)	Placebo (N = 15)	Overall (N = 45)
Parameter (Unit)	Not Hispanic or Latino^a^	Not Hispanic or Latino	Not Hispanic or Latino	Not Hispanic or Latino
Age (Years)				
n	15	15	15	45
Mean (SD)	25.9 (5.51)	27.5 (5.40)	26.3 (4.37)	26.6 (5.04)
Min, Max	18, 39	21, 38	19, 35	18, 39
Sex (n [%])				
Female	5 (33.3)	5 (33.3)	6 (40.0)	16 (35.6)
Male	10 (66.7)	10 (66.7)	9 (60.0)	29 (64.4)
Race (n [%])				
American Indian or Alaska Native	0	0	0	0
Asian	4 (26.7)	2 (13.3)	3 (20.0)	9 (20.0)
Black or African American	1 (6.7)	0	0	1 (2.2)
Native Hawaiian or Other Pacific Islander	0	0	0	0
White	10 (66.7)	13 (86.7)	12 (80.0)	35 (77.8)
Other	0	0	0	0

^a^ No Hispanic/Latino participants were enrolled in the study; 2 of 18 screen failures were Hispanic/Latino.

### Reactogenicity and tolerability

All treatment groups reported local symptoms within 7 days after vaccination, with erythema being the most frequent local symptom reported (15 participants [100%] in the 15 μg treatment group, 12 participants [80.0%] in the 7.5 μg treatment group, and 14 participants [93.3%] in the placebo group), followed by induration/swelling (9 participants [60.0%] in the 15 μg treatment group, 6 participants [40.0%] in the 7.5 μg treatment group, and 3 participants [20.0%] in the placebo group). Itching, pigmentation, skin flaking, and tenderness were also reported.

**Fig 2:**
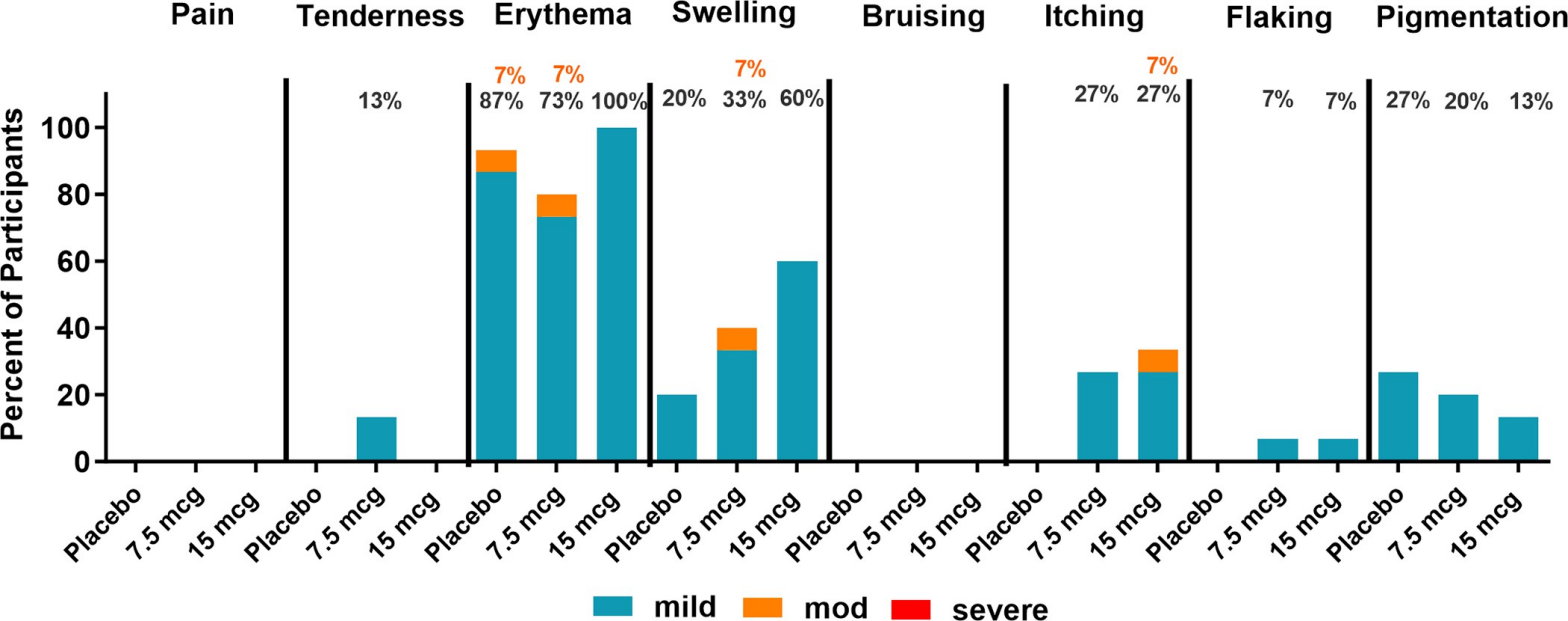
Solicited local reactogenicity events 7 days after vaccination. The incidence of local events is shown overall, by severity, and by treatment group. N = 15 / treatment group.

No severe reactogenicity events were reported in the study. The most severe local injection site symptoms reported within 7 days after vaccination for all treatment groups were mild (Grade 1) erythema (39 participants [86.7%]), moderate (Grade 2) erythema (2 participants [4.4%], mild (Grade 1) induration/swelling (17 participants [37.8%]), moderate (Grade 2) induration/swelling (1 participant [2.2%]), mild (Grade 1) itching (8 participants [17.8%]), moderate (Grade 2) itching (1 participant [2.2%]), and mild (Grade 1) pigmentation (9 participants [20.0%]). Mild (Grade 1) skin flaking was reported by 1 participant each in the 7.5 μg and 15 μg treatment groups (4.4% overall), and mild (Grade 1) tenderness was reported by 2 participants in the 7.5 μg treatment group (4.4% overall) (**Fig 2 and S1 Table**).

**Fig 3:**
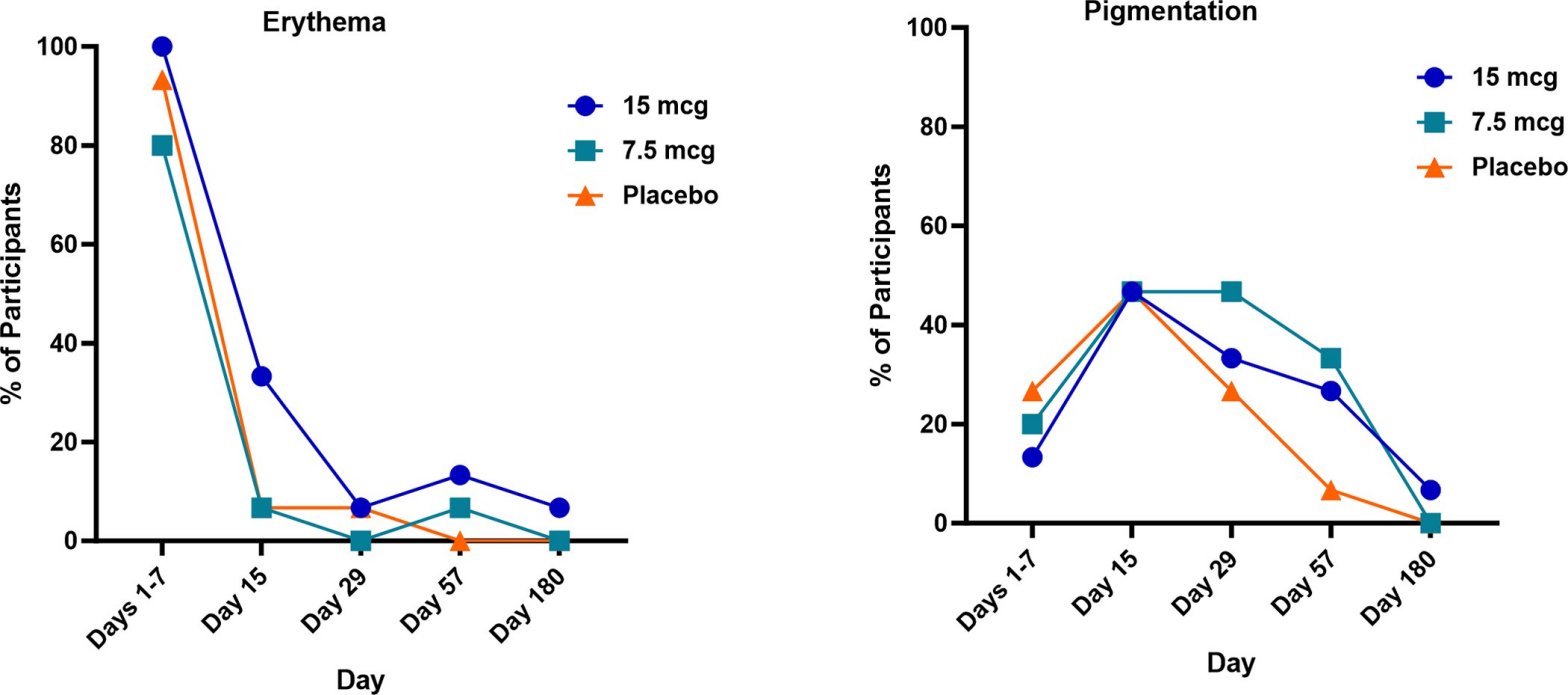
Resolution of MAP application site erythema and pigmentation over time. The percentage of participants reporting erythema (left) and/or pigmentation (right) through 7 days post vaccination and then post study day 8 is shown by treatment group. Participants reporting erythema and pigmentation concurrently are counted twice.

Both erythema and pigmentation were followed post Study Day 8. By Study Days 15 and 29, there was a reduction in mild (Grade 1) erythema reported (7 participants [15.6%], 2 participants [4.4%], respectively) as discoloration of the application site transitioned from mild erythema to mild pigmentation. There was also an overall reduction in mild (Grade 1) pigmentation reported on Days 15, 29, 57, and 180 (21 participants [46.7%], 16 participants [35.6%], 10 participants [22.2%], and 1 participant [2.2%] respectively) (**Fig 3 and S2 Table**).

**Fig 4:**
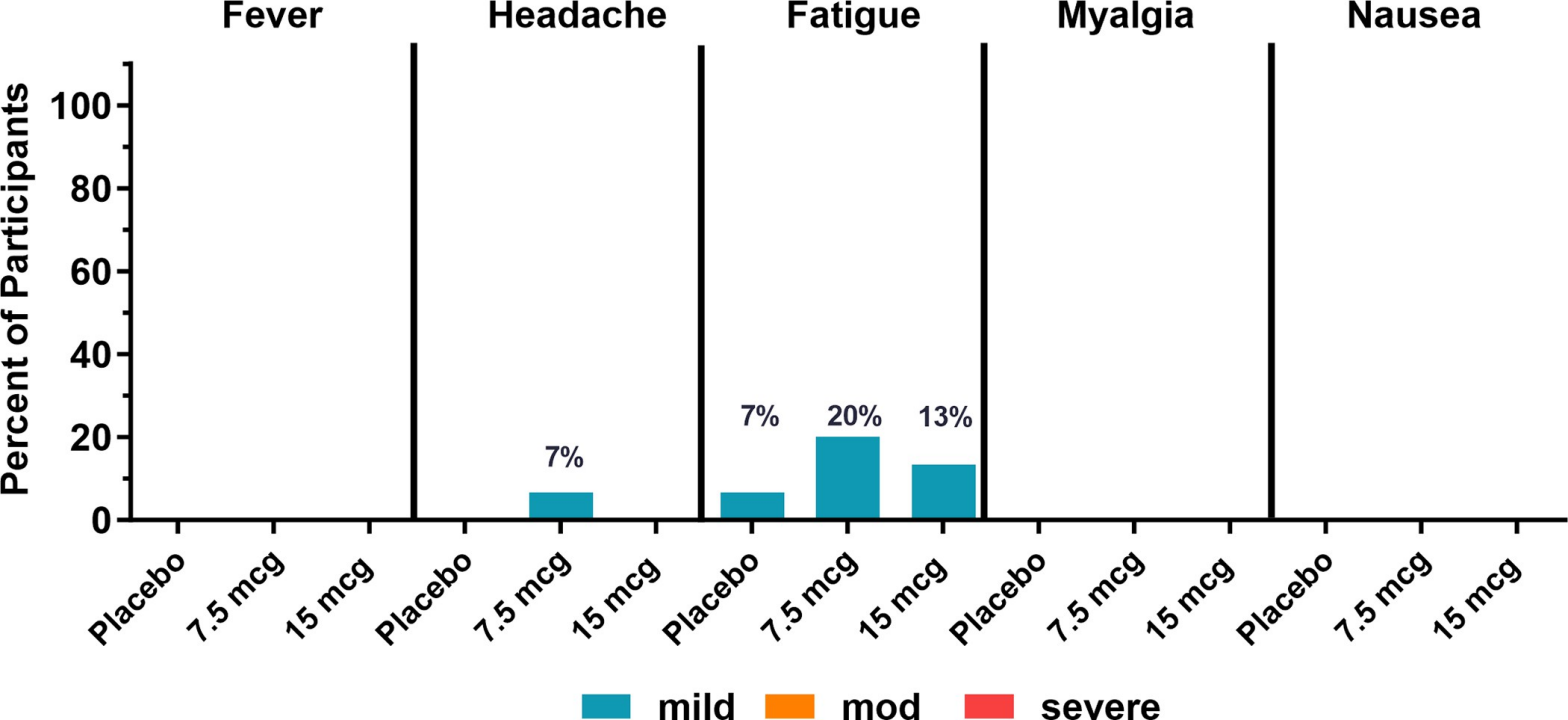
Solicited systemic reactogenicity events 7 days after vaccination. The incidence of local events is shown overall, by severity, and by treatment group. N = 15 / treatment group.

Within 7 days after vaccination, mild (Grade 1) fatigue was reported among all treatment groups (2 participants [13.3%] in the 15 μg treatment group, 3 participants [20.0%] in the 7.5 μg treatment group, and 1 participant [6.7%] in the placebo group). Mild (Grade 1) headache was reported by 1 participant [6.7%] in the 7.5 μg treatment group (**Fig 4 and S1 Table**).

Four (9%) participants experienced a vaccine-related or possibly related, unsolicited TEAE, including transient mild (Grade 1) application site discomfort occurring 30 minutes post treatment for a participant in the 15 μg dose group and resolving in 2 minutes; and moderate (Grade 2) application site pain occurring 24 hours post treatment for a participant in the 15 μg dose group and resolving in 30 minutes; moderate (Grade 2) oropharyngeal pain (7.5 μg dose group); and mild (Grade 1) chills (placebo group). There were 3 severe (Grade 3) TEAEs of blood creatine phosphokinase increase reported in 3 participants (20.0%) in the 7.5 μg treatment group that were considered not related to study drug. These increases occurred in 3 male participants, 21–23 years old. Peak values were 1306, 1917 and 3383 U/L (the normal range used for the laboratory data was 0–224 U/L). The increases were deemed by the investigator to be related to gym overuse.

No participant experienced an SAE or discontinued from the study because of a TEAE. Furthermore, with the exception of the cases of CPK TEAEs noted above, no other laboratory findings were considered clinically significant, with no clinically relevant changes within or across treatment groups seen. No clinically relevant trends in changes or clinically significant abnormalities in vital signs were noted.

### Immunogenicity

HAI GMTs, HAI median titers, group mean-fold rises, SCR and SPR against the vaccine strain are summarized in **Table 2**. VX-103 elicited statistically significant rises in HAI GMTs with group mean-fold rises ranging between 8.7 (p = 0.0041 vs placebo) and 12-fold (p = 0.0017 vs placebo) for the 7.5 and 15 μg dose levels respectively. Post dose geometric mean titers differed significantly from the placebo group for each dose level and at each post vaccination timepoint (p ≤ 0.007 for all timepoints) but not between dose levels suggesting dose sparing potential. However, with a larger study N the trend towards lower titers for the 7.5 μg versus the 15 μg dose level might have met statistical significance.

**Table 2:** Summary of Hemagglutinin Inhibition Assay (HAI) antibody titers, seroconversion, and Seroprotection rates (immunogenicity analysis set).

Visit Statistics	VX-103 15 μg (N = 14)	VX-103 7.5 μg (N = 14)	Placebo (N = 15)
**Day 1 –Pre-Vaccination**			
** HAI Titers: n**	14	14	15
GM	45.7	32.8	33.8
95% C.I.	(24.72, 84.39)	(14.00, 76.89)	(18.92, 60.28)
Median	40	40	40
IQR	51	95	60
** Seroprotection Rate**			
n (%)	10 (71.4)	9 (64.3)	10 (66.7)
95% C.I.	(47.76, 95.09)	(39.19, 89.39)	(42.81, 90.52)
p-Value^[7]^			0.9190
**Day 29**			
** HAI Titers: n**	13	14	15
GM	473.2	285.1	34.3
95% C.I.	(181.88, 1230.95)	(136.59, 595.02)	(20.52, 57.29)
Median	508	361.5	40
IQR	2379	594	60
GMR^[^^1^^]^	13.80	8.31	1.00
GMR 95% C.I.			(17.02, 47.57)
p-Value^[2]^	0.0016	0.0005	
p-Value^[^^2^^]^		0.0984	
** Day 29 Fold Increase: n**	13	14	15
GM^[^^3^^]^	12.0	8.7	1.0
95% C.I.	(5.48, 26.41)	(4.57, 16.51)	(0.91, 1.14)
Mean (SD)	23.8(25.16)	15.0(17.28)	1.0(0.28)
p-Value^[^^4^^]^	0.0017	0.0041	
p-Value^[^^5^^]^		0.2999	
** Seroconversion Rate Day 29**			
n (%)	10 (76.9)	11 (78.6)	0
95% C.I.	(54.02, 99.83)	(57.08, 100.00)	N/A
p-Value^[^^7^^]^			<0.0001
Difference Across Groups^[^^6^^]^		0.0012	
** Seroprotection Rate Day 29**			
n (%)	12 (92.3)	13 (92.9)	10 (66.7)
95% C.I.	(77.82, 100.00)	(79.37, 100.00)	(42.81, 90.52)
p-Value^[^^7^^]^			0.0969
**Day 57**			
HAI Titers: n	13	13	15
GM	433.0	282.6	36.5
95% C.I.	(175.69, 1066.91)	(115.05, 694.24)	(21.25, 62.60)
Median	640	320	40
IQR	1266	1028	60
GMR^[^^1^^]^	11.87	7.75	1.00
GMR 95% C.I.			(15.87, 46.98)
p-Value^[^^2^^]^	0.0024	0.0007	
p-Value^[^^2^^]^		0.2945	
**Day 57-Fold Increase: n**	13	13	15
GM^[^^3^^]^	11.0	7.5	1.1
95% C.I.	(5.42, 22.37)	(3.74, 14.85)	(0.90, 1.30)
Mean (SD)	19.8(22.93)	13.8(17.77)	1.1(0.41)
p-Value^[^^4^^]^	0.0039	0.0100	
p-Value^[^^5^^]^		0.4638	
** Seroconversion Rate Day 57**			
n (%)	11 (84.6)	10 (76.9)	0
95% C.I.	(65.00, 100.00)	(54.02, 99.83)	N/A
p-Value^[^^7^^]^			<0.0001
Difference Across Groups^[^^6^^]^		0.0023	
** Seroprotection Rate Day 57**			
n (%)	12 (92.3)	12 (92.3)	10 (66.7)
95% C.I.	(77.82, 100.00)	(77.82, 100.00)	(42.81, 90.52)
p-Value^[^^7^^]^			0.1099
**Day 85**			
** HAI Titers: n**	14	14	14
GM	396.6	218.9	32.8
95% C.I.	(183.32, 857.91)	(95.46, 502.05)	(18.87, 57.07)
Median	361.5	320	40
IQR	828	500	62.5
GMR^[^^1^^]^	12.09	6.67	1.00
GMR 95% C.I.			(19.57, 49.55)
p-Value^[2]^	0.0021	0.0011	
p-Value^[^^2^^]^		0.1623	
** Day 85 Fold Increase: n**	14	14	14
GM^[^^3^^]^	8.7	6.7	1.0
95% C.I.	(4.41, 17.10)	(3.52, 12.64)	(0.92, 1.16)
Mean (SD)	15.2(16.86)	10.9(10.40)	1.1(0.28)
p-Value^[^^4^^]^	0.0041	0.0016	
p-Value^[^^5^^]^		0.4171	
** Seroconversion Rate Day 85**			
n (%)	10 (71.4)	11 (78.6)	0
95% C.I.	(47.76, 95.09)	(57.08, 100.00)	N/A
p-Value^[^^7^^]^			<0.0001
Difference Across Groups^[^^6^^]^		0.0012	
** Seroprotection Rate Day 85**			
n (%)	13 (92.9)	13 (92.9)	9 (64.3)
95% C.I.	(79.37, 100.00)	(79.37, 100.00)	(39.19, 89.39)
p-Value^[^^7^^]^			0.0644
**Day 119**			
** HAI Titers: n**	14	14	13
GM	341.8	185.6	36.0
95% C.I.	(156.91, 744.63)	(80.15, 429.87)	(19.48, 66.36)
Median	341.8	287.0	40
IQR	764.5	570	65
GMR^[^^1^^]^	9.51	5.16	1.00
GMR 95% C.I.			(21.01, 50.93)
p-Value^[2]^	0.0060	0.0012	
p-Value^[^^2^^]^		0.1588	
** Day 119 Fold Increase: n**	14	14	13
GM^[^^3^^]^	7.5	5.7	1.0
95% C.I.	(3.72, 15.06)	(2.79, 11.45)	(0.82, 1.17)
Mean (SD)	14.4(17.63)	10.3(10.78)	1.0(0.33)
p-Value^[^^4]^	0.0116	0.0050	
p-Value^[^^5^^]^		0.4623	
** Seroconversion Rate Day 119**			
n (%)	9 (64.3)	10 (71.4)	0
95% C.I.	(39.19, 89.39)	(47.76, 95.09)	N/A
p-Value^[^^7^^]^			0.0003
**Difference Across Groups**^[^^6^^]^		0.0002	
** Seroprotection Rate Day 119**			
n (%)	13 (92.9)	13 (92.9)	9 (69.2)
95% C.I.	(79.37, 100.00)	(79.37, 100.00)	(44.14, 94.32)
p-Value^[^^7^^]^			0.1376
**Day 180**			
** HAI Titers: n**	14	14	15
GM	289.9	179.6	35.4
95% C.I.	(132.30, 635.12)	(78.95, 408.56)	(20.55, 60.86)
Median	320.0	240.0	40
IQR	574.0	534.7	60
GMR^[^^1^^]^	8.20	5.08	1.00
GMR 95% C.I.			(18.08, 48.09)
p-Value^[^^2^^]^	0.0076	0.0006	
p-Value^[^^2^^]^		0.2291	
** Day 180 Fold Increase: n**	14	14	15
GM^[^^3^^]^	6.3	5.5	1.0
95% C.I.	(3.13, 12.86)	(2.79, 10.75)	(0.86, 1.28)
Mean (SD)	12.3(16.60)	9.8(10.62)	1.1(0.49)
p-Value^[^^4^^]^	0.0144	0.0039	
p-Value^[^^5^^]^		0.6311	
** Seroconversion Rate Day 180**			
n (%)	10 (71.4)	9 (64.3)	0
95% C.I.	(47.76, 95.09)	(39.19, 89.39)	N/A
p-Value^[^^7^^]^			0.0001
Difference Across Groups^[^^6^^]^		0.0001	
** Seroprotection Rate Day 180**			
n (%)	13 (92.9)	13 (92.9)	10 (66.7)
95% C.I.	(79.37, 100.00)	(79.37, 100.00)	(42.81, 90.52)
p-Value^[^^7^^]^			0.0856

**Abbreviations:** ANOVA = analysis of variance; CI = confidence interval; GM = geometric mean; GMR = geometric mean ratio; GMT = geometric mean titer; HAI = hemagglutinin inhibition; n = number of participants in the respective category; N = number of participants in the Immunogenicity Analysis Set; N/A = not applicable; SD = standard deviation.

**Note:** GM and 2-sided 95% CIs were based on the log-transformed data, back-transformed to the original scale. The N’s may have changed for each subsequent visit.

[1] GMR was defined as GMT of vaccine group / GMT of Placebo. CI is calculated from an exact binomial

[2] p-value is from comparison of each vaccine dose GMT with Placebo and 7.5 μg group GMT with 15 μg vaccine group by ANOVA.

[3] Fold increase is defined as the GMT of the HAI titer increase from Day 1 pre-vaccination for each timepoint

[4] p-value is from comparison of GM Fold Rise by Student’s t test. The difference in arithmetic mean HAI titer fold increase may be compared across treatment groups using ANOVA. The ANOVA model included Treatment group as a fixed effect.

[5] p-value is from comparison of GM Fold Rise with 15 μg vaccine group by Student’s t test. The difference in arithmetic mean HAI titer fold increase may be compared across treatment groups using ANOVA.

[6] Seroconversion and seroprotection rates were by chi-square test.

[7] p-value is from chi-square test for comparison between all the treatment groups.

SCRs were comparable for each VX-103 dose group and differed significantly from placebo (p<0.0001) with 10 of 13 [76.9%] and 11 of 14 [78.6%] participants seroconverting in the 15 μg and 7.5 μg treatment groups, respectively, at Day 29. At Day 180, 10 of 14 [76.9%] and 9 of 14 [64.3%] participants remained seroconverted in the 15 μg and 7.5 μg treatment groups, respectively. SPRs at Day 29 were 12 of 14 [92.3%] and 13 of 14 [92.9%] in the 15 μg and 7.5 μg treatment groups, respectively, versus 10 of 15 [66.7%] in the placebo group. At Day 180, SPRs remained at 13 of 14 [92.9%] in both treatment groups, versus 10 of 15 [66.7%] in the placebo group. SPR did not differ significantly from the placebo group at either timepoint, reflecting the high baseline titers and rates of seroprotection at study start.

**Fig 5:**
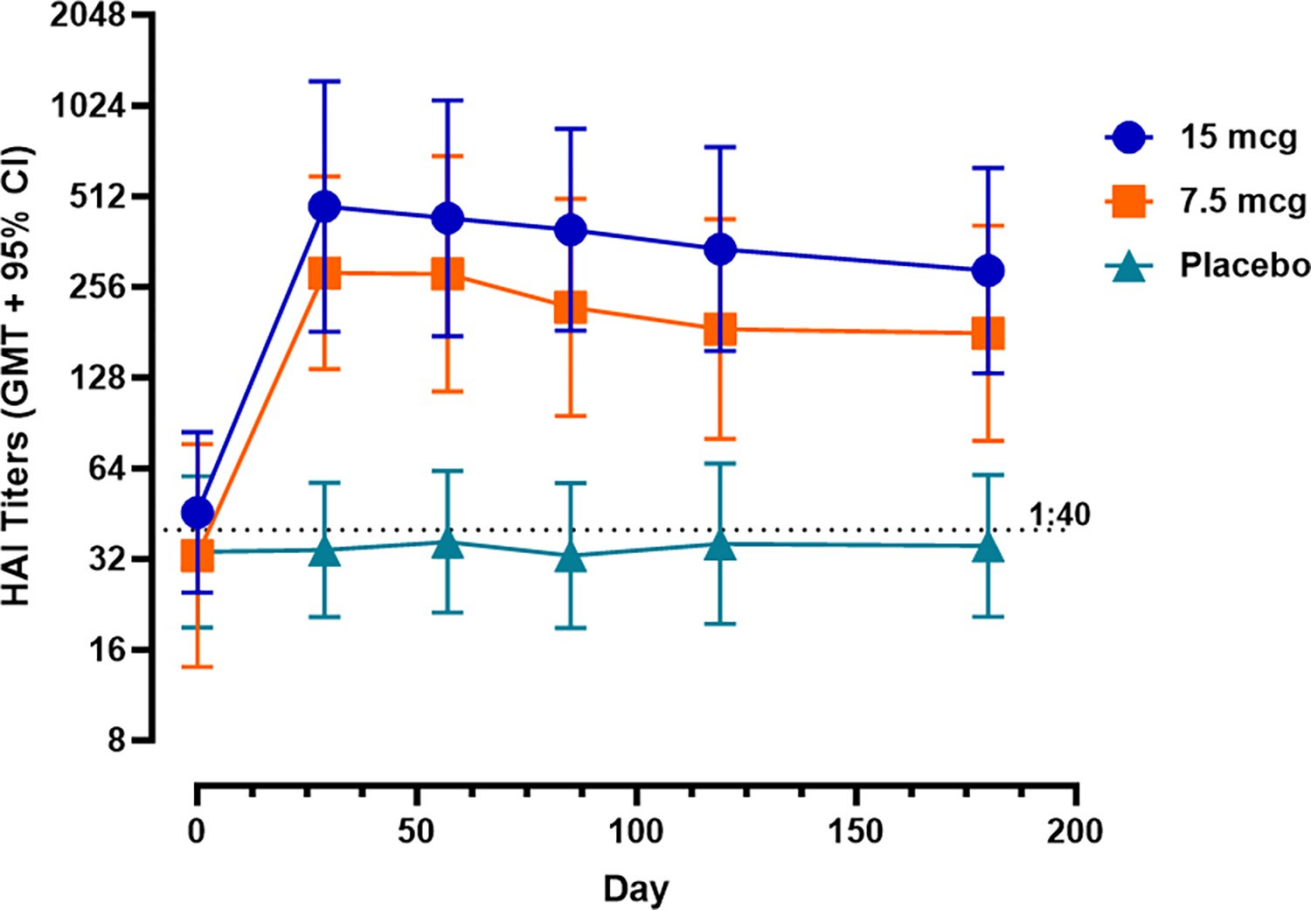
Longitudinal HAI titers by treatment group and study day. Lines depict the group GM titer and 95% CI at each timepoint for each treatment group. The dotted line at 1:40 represents seroprotective levels of antibody. n = 14 for 7.5 and 15 μg treatment groups and 15 for the placebo treatment group.

Longitudinal GM HAI titers, by treatment group, presented in **Fig 5** (S1 Dataset) further demonstrate persistence of the immune response through at least Day 180.

**Fig 6:**
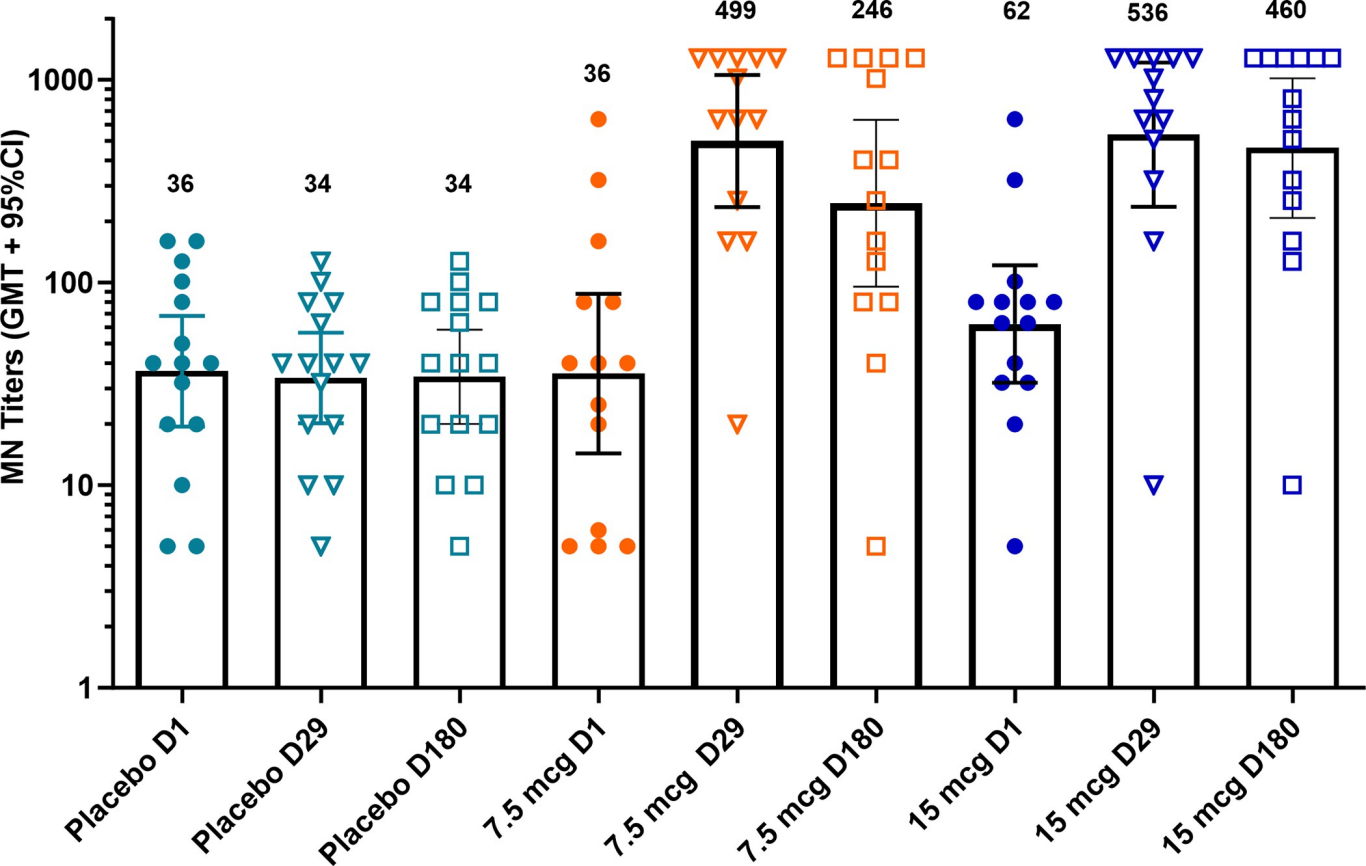
Virus MN titers through day 180. Data points depict GM MN titers for each subject. Group GM ± 95% confidence intervals are depicted by columns and are indicated above each dataset. n = 14 for 7.5 and 15 μg treatment groups and 15 for the placebo treatment group.

Virus MN titers were an exploratory immunogenicity assessment measured on sera collected at Days 1, 29, and 180 (**Fig 6 and S2 Dataset**). Microneutralization titers did not rise in the placebo group. In comparison to the placebo group, statistically significant rises in GM MN titers were observed for both dose levels of VX-103 (p<0.0001 for both dose levels at both timepoints). MN titers correlated strongly with HAI titers (Pearson correlation coefficient r = 0.784; p<0.001 at Day 29; r = 0.755; p <0.001 at Day 180).

**Fig 7:**
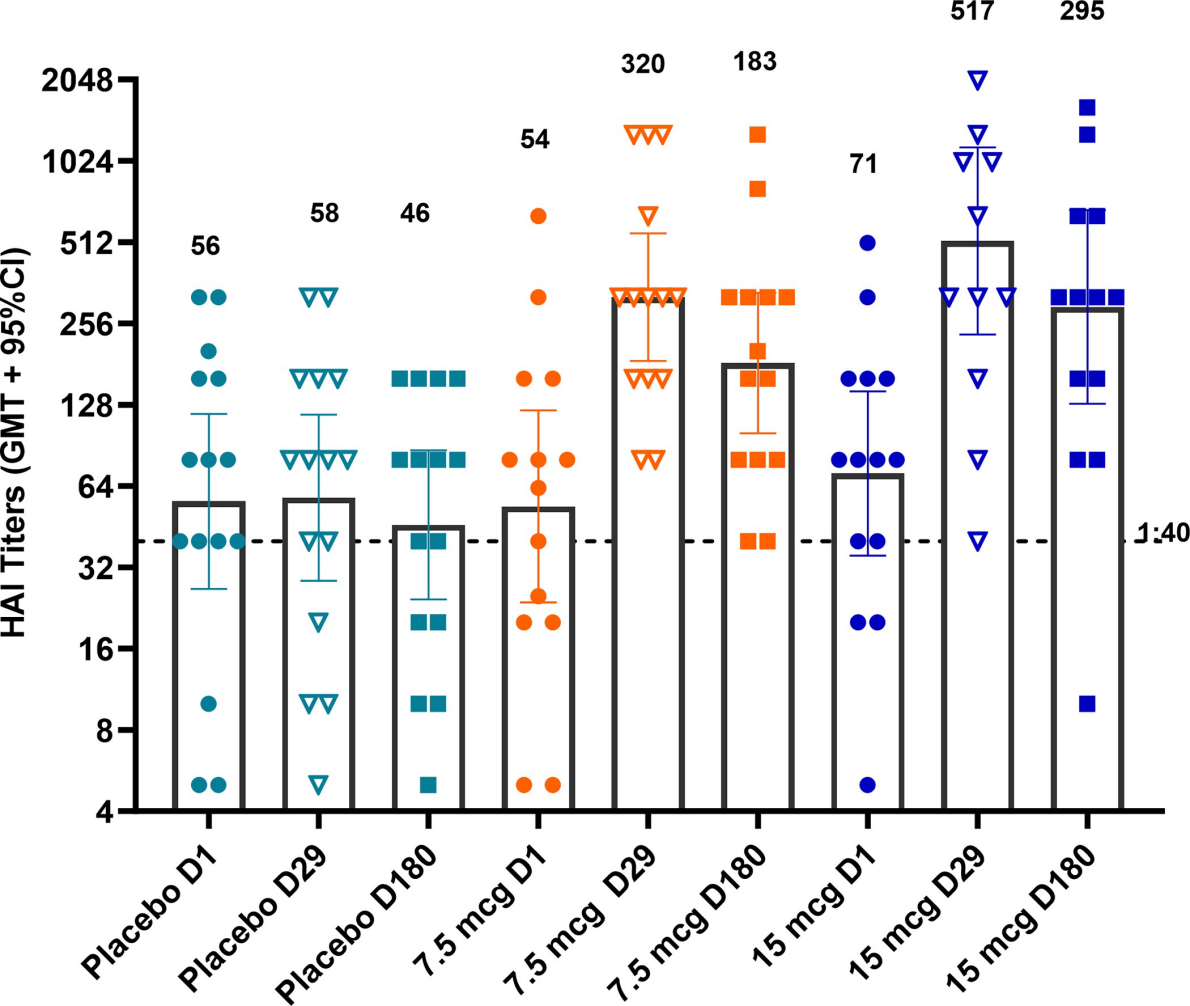
HAI titers against an antigenically drifted strain. HAI titers against the A/Victoria/2570/2019 (H1N1 6B.1A.5a2) strain are shown by treatment group. Data points depict GM HAI titers for each subject. Group GM ± 95% confidence intervals are depicted by columns and are indicated above each dataset. The dotted line at 1:40 represents seroprotective levels of antibody. n = 14 for 7.5 and 15 μg treatment groups and 15 for the placebo treatment group.

In an additional exploratory assessment, sera collected on Days 1, 29 and 180 were tested in the HAI assay against the antigenically drifted strain A/Victoria/2570/2019. As shown in **Fig 7 (S3 Dataset)**, no rise in HAI titers was observed in the placebo group, while both dose levels of VX-103 elicited significant rises in HAI titers against the drifted strain (p<0.0001 at Day 29 and p<0.005 at Day 180 for both dose levels compared to placebo).

HAI titers against the A/Victoria/2570/2019 strain correlated strongly with HAI titers against the vaccine strain at both Study Days 29 and 180 (**S1 Fig**; Pearson correlation coefficient r = 0.910; p<0.001 at Day 29; r = 0.879; p <0.001 at Day 180).

In a final exploratory assessment, anti-HA IgG titers against the A/Guangdong H1N1 strain measured at Study Days 29 and 180 were found to correlate strongly with HAI titers for both dose levels of VX-103 (**S2 Fig,** Pearson correlation coefficient r = 0.947; p<0.0001 at Day 29; r = 0.924; p <0.0001 at Day 180). No rises in A/Guangdong H1N1 HA specific IgG were observed in the placebo group.

## Discussion

This study showed for the first time in a human clinical trial that influenza vaccination with a MIMIX MAP is generally safe, well tolerated, and immunogenic. It is notable that no solicited pain or bruising and only limited application site tenderness was reported in the study. Pruritis, or itching, is often a frequent complaint with intradermally delivered vaccines [23,35]. In our study, itching was generally mild, limited to a subset of participants receiving the vaccine and resolved within several days post vaccination. The relatively high rates of erythema and pigmentation are consistent with previous reports describing intradermal vaccination either by needle or syringe [36,37] or by a different MAP platform [16,21]. Erythema and pigmentation at the application site did take longer to resolve for vaccine-containing MAPs. This is consistent with observations for other MAP platforms [15,23,38] and suggests that the localized discoloration is immune mediated. The high rate of erythema in the placebo group is presumably related to the device constituents of VX-103.

The frequency of systemic symptoms reported for participants receiving the vaccine was remarkably low. The low frequency of systemic events is similar to that reported by Fernando et al [15] where a single H1N1 influenza vaccine was delivered with the coated microneedle array, Nanopatch. While it is tempting to attribute lower systemic reactogenicity to intradermal delivery both Marra and Egunsola [7,10] found that the risks of systemic adverse events, such as headache, malaise, and myalgia were similar with intradermal and intramuscular administration methods. Thus, the MIMIX MAP platform may have a more favorable systemic safety profile than delivery by needle and syringe perhaps due to the sustained release of vaccine antigen from the MAP.

The FDA and EMA have established criteria for immunological endpoints that are based on HAI titers and which may be used as supporting evidence, or surrogates, of vaccine efficacy for seasonal influenza vaccines. In young healthy adults the absolute criteria for these surrogate endpoints specify a SCR of >40%, a SPR of >70% and a GMT fold increase of 2.5 [34,39]. VX-103-elicited fold increases in GM HAI titers that ranged between 8.7 and 12-fold for the 7.5 and 15 μg treatment groups respectively thereby fulfilling this aspect of FDA and EMA criteria. For context, in Phase 3 studies supporting licensure of Fluzone® Quadrivalent [40] and Fluarix Quadrivalent [41]vaccines, GMT fold increases to the H1N1 component were 14.3 (n = 1112) and 10.6 (n = 257) respectively.

SPR and SCR also fulfilled EMA and FDA criteria for both dose levels of VX-103. At Study Day 29, VX-103-elicited SPR were 92.6% (7.5 μg dose group) and 92.3% (15 μg dose group) while SCR were 78.6% (7.5 μg dose group) and 76.9% (15 μg dose group). For further context, in the Fluarix® and Fluzone® Phase 3 studies referenced above, SPR to the H1N1 strain were 91.8% [41] and 96.4% [40] while SCR to the H1N1 strain were 71.6% [41] and 72.2% [40].

In sum, VX-103 elicited immune responses met EMA and FDA criteria for surrogate endpoints and were similar in magnitude and response rates to those reported in Phase 3 evaluations of licensed influenza vaccines.

Baseline SPR for the 15 μg, 7.5 μg and placebo groups were notably high at 71.4%, 64.3% and 66.7% respectively and show that over 60% of the subjects enrolled in the study had seroprotective levels of HAI titers prior to vaccination. Because exclusion criteria excluded participants receiving an influenza vaccine 2 years prior to study enrollment, the high baseline titers are likely related to natural exposure. It is generally accepted that high baseline titers actually have a negative impact on the ability to achieve a 4-fold rise in the HAI response (i.e. seroconversion rate) [42]. Still, VX-103 elicited SCR and SPR continued to meet FDA and EMA criteria through Day 180 for both dose groups. These data are important given the interest in dose sparing and in developing vaccines that ensure protective levels of immune response throughout the influenza season. Longitudinal response curves for each treatment group further demonstrated that VX-103 elicited titers largely persisted through at least day 180.

Although the HAI assay is commonly used to measure serum antibodies against influenza, it has been criticized for its lack of mechanistic relevance to natural influenza infection of eukaryotic cells. In contrast, the virus MN assay is a functional assessment that measures the concentration of antibodies required to prevent infection of cells. Both Tsang and Verschoor have demonstrated that MN titers correlate with protection in household contacts and children respectively [43,44]. More recently, Heeringa has demonstrated that there is a strong correlation between HAI and MN titers [45]. As an exploratory objective, we assessed MN titers and observed statistically significant rises for both dose levels of VX-103 at Days 29 and 180 that correlated strongly with HAI titers. Microneutralization titers did not rise in the placebo group.

Antigenic drift among influenza viruses is a process of selection for naturally occurring virus variants under the pressure of population immunity. Under this process, mutations in the major protective antigen, HA, accumulate at a frequency of approximately 1% per year, such that every 2 to 5 years an antigenically distinct strain emerges that is poorly neutralized by antibodies raised on prior strains, requiring updating of seasonal influenza vaccines. Because antigenic drift is constantly occurring, influenza vaccines that elicit antibodies that recognize ‘antigenically drifted’ strains are of interest. In 2019 the H1N1 pdm09 subclade 6B.1A.5 split into 2 antigenically distinct groups based on reactivity with ferret immune sera. This antigenic difference led to a recommendation by the WHO to replace the H1N1 Guangdong strain with the A Victoria strain as the H1N1 component for the NH seasonal vaccine in the 2021–2022 season. In a final exploratory objective, VX-103 was found to elicit robust, durable HAI titers against this antigenically distinct influenza A strain (A/Victoria/2570/2019) that emerged in an adjacent influenza season.

In sum, both dose levels elicited durable HAI and MN titers against the vaccine strain, demonstrating that VX-103 elicits persistent immune responses and has dose-sparing potential. Both dose levels elicited robust immune responses to an antigenically drifted influenza strain, demonstrating the potential for broadly reactive immune responses.

There are several limitations to the study. Inclusion of an IM delivered, licensed comparator would have facilitated interpretation of the data as well as comparison of safety and immunogenicity for the two different routes of vaccination. However, the H1N1 strain used in the study was mismatched with regards to the H1N1 strain in concurrent licensed seasonal vaccine. To include an IM comparator therefore would have required us to formulate a second investigational product. For logistical reasons, we then elected to defer comparisons of the two vaccination routes to a study that can include an antigen-matched, licensed comparator. The small group sizes also limited statistical power for evaluating dose sparing potential and durability of the immune response.

## Conclusion

A single 15 μg or 7.5 μg dose of VX-103 delivered via MIMIX MAP immunization to healthy participants was generally safe and well-tolerated with mostly mild side effects. A single 15 μg or 7.5 μg dose of VX-103 delivered via MIMIX MAP immunization to healthy participants is immunogenic, with both dose levels eliciting durable HAI, MN, and anti-HAI titers against the vaccine strain, demonstrating that VX-103 elicits persistent immune responses through at least day 180 and has dose-sparing potential. Both dose levels elicited robust immune responses to an antigenically drifted influenza strain, demonstrating the potential for broadly reactive immune responses.

## Supporting information

S1 Checklist CONSORT 2010 checklist of information to include when reporting a randomised trial*. (DOC)

S1 Fig Correlation of HAI titers against the A/ Guangdong-Maonan/SWL 1536/2019-like H1N1 and A/Victoria/2570/2019 H1N1 strains at study days 29 and 180. For the 7.5 and 15 μg dose groups, HAI titers elicited against the H1N1 Guangdong strain were plotted against GM titers elicited against the H1N1 Victoria strain for each participant at Study Days 29 (left) and 180 (right). Pearson correlation coefficient and p values are shown in each graph.
(TIF)

S2 Fig Anti HA IgG Titers Against the A/Guangdong-Maonan/ SWL1536/2019CNIC-1909 strain. GM **(**+/- 95% CI) IgG Titers against the H1N1 Guangdong strain were measured by ELISA. For the 7.5 and 15 μg dose groups, HA IgG titers are plotted against HAI titers for each participant at Study Days 29 (left) and 180 (right). Pearson correlation coefficient and p values are shown in each graph.
(TIF)

S1 Table Local injection site symptoms and system symptoms within 7 days after vaccination (safety analysis set). Source data for Figs 2 and 4 taken from the VX103-01 clinical data package published according to data standards set for the by the Clinical Data Interchange Standards Consortium.
(PDF)

S2 Table Abbreviated local injection site symptoms occurring 15, 29, 57 and 180 days after vaccination (safety analysis set). Source data for Fig 3 taken from the VX103-01 clinical data package published according to data standards set for the by the Clinical Data Interchange Standards Consortium.
(PDF)

S1 Text Clinical trial protocol. (PDF)

S1 Dataset Individual reciprocal geometric mean HAI titers against the A/ Guangdong-Maonan/SWL 1536/2019-like H1N1 strain by study day and treatment. (XLSX)

S2 Dataset Individual reciprocal microneutralization titers against the A/ Guangdong-Maonan/SWL 1536/2019-like H1N1 strain by study day and treatment. (XLSX)

S3 Dataset Individual reciprocal geometric mean HAI titers against the A/Victoria/2570/2019 H1N1 strain by study day and treatment. (XLSX)
