# Supplementary figures and images for: Phase 1, randomized, rater and participant blinded placebo-controlled study of the safety, reactogenicity, tolerability and immunogenicity of H1N1 influenza vaccine delivered by VX-103 (a MIMIX microneedle patch [MAP] system) in healthy adults
Source: PLoS One. 2024 Jun 6;19(6):e0303450. doi: 10.1371/journal.pone.0303450 (PMC11156369; doi:10.1371/journal.pone.0303450)

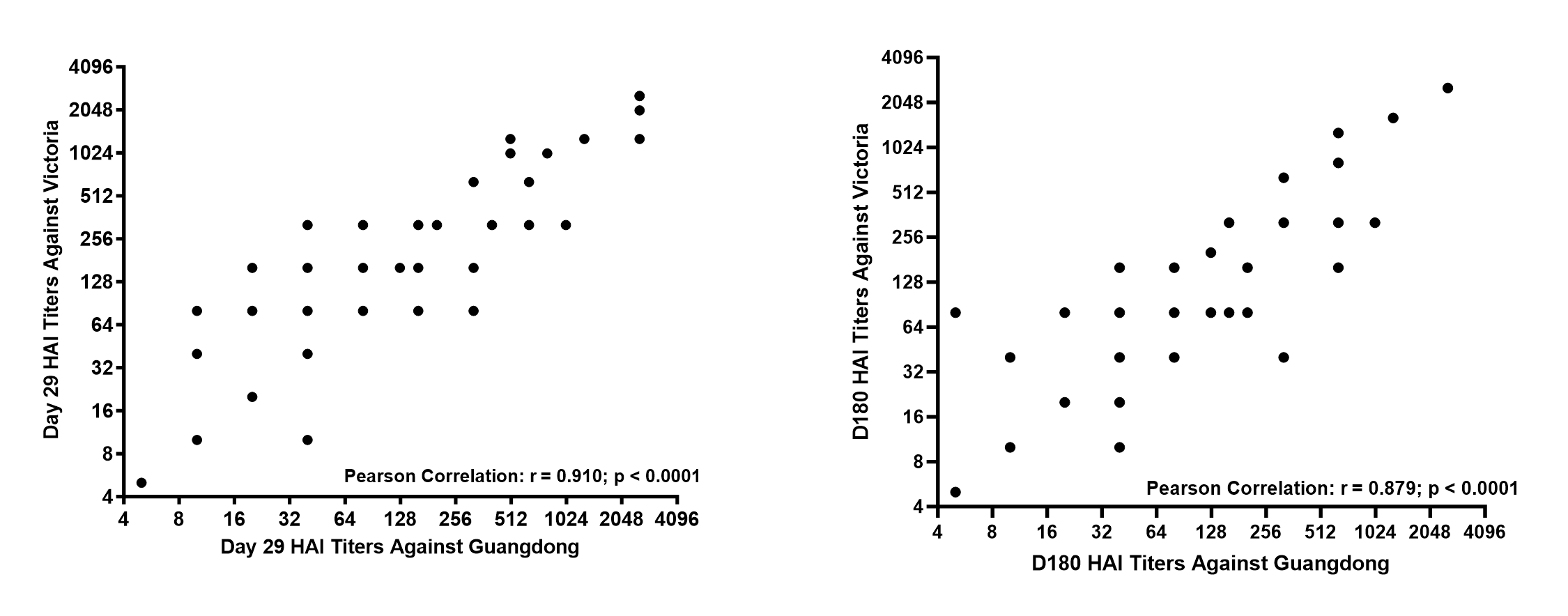

Supplement: S1 Fig — For the 7.5 and 15 μg dose groups, HAI titers elicited against the H1N1 Guangdong strain were plotted against GM titers elicited against the H1N1 Victoria strain for each participant at Study Days 29 (left) and 180 (right). Pearson correlation coefficient and p values are shown in each graph. (TIF) [file pone.0303450.s002.tif]

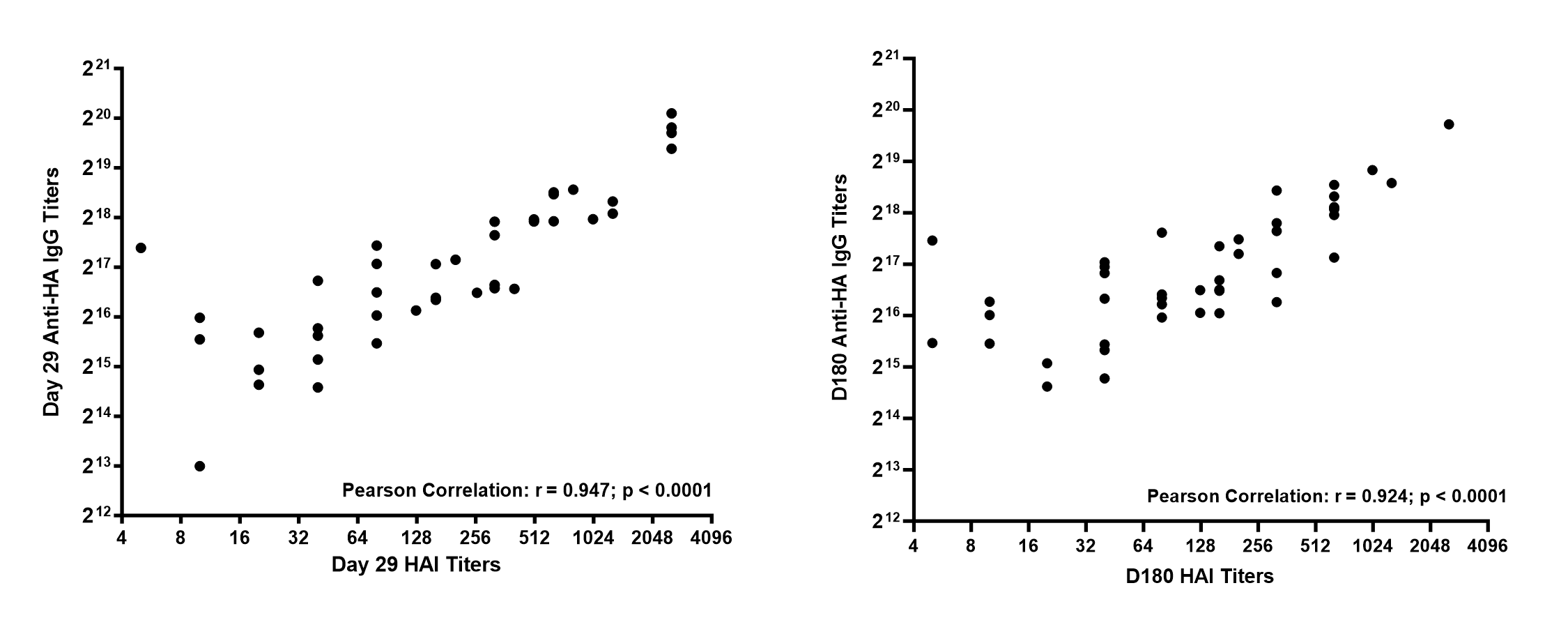

Supplement: S2 Fig — GM (+/- 95% CI) IgG Titers against the H1N1 Guangdong strain were measured by ELISA. For the 7.5 and 15 μg dose groups, HA IgG titers are plotted against HAI titers for each participant at Study Days 29 (left) and 180 (right). Pearson correlation coefficient and p values are shown in each graph. (TIF) [file pone.0303450.s003.tif]
