# Supplementary material for: Phase 1, randomized, rater and participant blinded placebo-controlled study of the safety, reactogenicity, tolerability and immunogenicity of H1N1 influenza vaccine delivered by VX-103 (a MIMIX microneedle patch [MAP] system) in healthy adults
Source: PLoS One. 2024 Jun 6;19(6):e0303450. doi: 10.1371/journal.pone.0303450 (PMC11156369; doi:10.1371/journal.pone.0303450)
Supplement: S1 Table — Source data for Figs 2 and 4 taken from the VX103-01 clinical data package published according to data standards set for the by the Clinical Data Interchange Standards Consortium. (PDF) [file pone.0303450.s004.pdf]

Table 14.3.3.5.2
Local Injection Site Symptoms and System Symptoms within 7 days after Vaccination (Safety Analysis Set)

Reactogenicity Level<br>Symptom	VX-103 15 µg<br>(N=15)	VX-103 7.5 µg<br>(N=15)	Placebo<br>(N=15)	p-Value [1]	p-Value [2]	p-Value [3]
Any Complaints within 7 days after Vaccination						
n (%)	15 (100.0)	13 (86.7)	14 (93.3)	>0.999	>0.999	>0.999
95% C.I.	(78.20, 100.00)	(59.54, 98.34)	(68.05, 99.83)			
Any Local Symptoms						
n (%)	15 (100.0)	13 (86.7)	14 (93.3)	>0.999	>0.999	>0.999
95% C.I.	(78.20, 100.00)	(59.54, 98.34)	(68.05, 99.83)			
Erythema	15 (100.0)	12 (80.0)	14 (93.3)			
Induration/Swelling	9 (60.0)	6 (40.0)	3 (20.0)			
Itching	5 (33.3)	4 (26.7)	0			
Pigmentation	2 (13.3)	3 (20.0)	4 (26.7)			
Skin Flaking	1 (6.7)	1 (6.7)	0			
Tenderness	0	2 (13.3)	0			
Any Systemic Symptoms						
n (%)	2 (13.3)	3 (20.0)	1 (6.7)	>0.999	0.5977	0.6467
95% C.I.	(1.66, 40.46)	(4.33, 48.09)	(0.17, 31.95)			

Data Source: Listing 16.2.7.1
Note: At each level of subject summarization, a subject is counted once if they reported one or more events. Two-sided 95% Confidence Interval (C.I.) on the rate using the exact binomial.
Only Local Injection Site Symptoms and Systemic Symptoms were considered, as solicited Adverse Events were summarized.
[1] p-value from Fisher Exact test comparing 15 mcg vaccine and Placebo treatment groups
[2] p-value from Fisher Exact test comparing 7.5 mcg vaccine and Placebo treatment groups
[3] p-value from Fisher Exact test comparing 7.5 mcg and 15 mcg vaccine treatment groups

Table 14.3.3.5.2
Local Injection Site Symptoms and System Symptoms within 7 days after Vaccination (Safety Analysis Set)

Reactogenicity Level<br>Symptom	VX-103 15 µg<br>(N=15)	VX-103 7.5 µg<br>(N=15)	Placebo<br>(N=15)	p-Value [1]	p-Value [2]	p-Value [3]
Fatigue	2 (13.3)	3 (20.0)	1 (6.7)			
Headache	0	1 (6.7)	0			

Data Source: Listing 16.2.7.1
Note: At each level of subject summarization, a subject is counted once if they reported one or more events. Two-sided 95% Confidence Interval (C.I.) on the rate using the exact binomial.
Only Local Injection Site Symptoms and Systemic Symptoms were considered, as solicited Adverse Events were summarized.
[1] p-value from Fisher Exact test comparing 15 mcg vaccine and Placebo treatment groups
[2] p-value from Fisher Exact test comparing 7.5 mcg vaccine and Placebo treatment groups
[3] p-value from Fisher Exact test comparing 7.5 mcg and 15 mcg vaccine treatment groups
