# Supplementary material for: Phase 1, randomized, rater and participant blinded placebo-controlled study of the safety, reactogenicity, tolerability and immunogenicity of H1N1 influenza vaccine delivered by VX-103 (a MIMIX microneedle patch [MAP] system) in healthy adults
Source: PLoS One. 2024 Jun 6;19(6):e0303450. doi: 10.1371/journal.pone.0303450 (PMC11156369; doi:10.1371/journal.pone.0303450)
Supplement: S2 Table — Source data for Fig 3 taken from the VX103-01 clinical data package published according to data standards set for the by the Clinical Data Interchange Standards Consortium. (PDF) [file pone.0303450.s005.pdf]

Table 14.3.3.6.1
Abbreviated Local Injection Site Symptoms Occurring 15, 29, 57 and 180 days after Vaccination (Safety Analysis Set)

Reactogenicity Level<br>Symptom	Grade	VX-103 15 µg<br>(N=15)	VX-103 7.5 µg<br>(N=15)	Placebo<br>(N=15)	Total<br>(N=45)
Local Symptoms					
Day 15					
Erythema	1	5 (33.3)	1 (6.7)	1 (6.7)	7 (15.6)
Pigmentation	1	7 (46.7)	7 (46.7)	7 (46.7)	21 (46.7)
Day 29					
Erythema	1	1 (6.7)	0	1 (6.7)	2 (4.4)
Pigmentation	1	5 (33.3)	7 (46.7)	4 (26.7)	16 (35.6)
Day 57					
Erythema	1	2 (13.3)	1 (6.7)	0	3 (6.7)
Induration/Swelling	1	1 (6.7)	0	0	1 (2.2)
Pigmentation	1	4 (26.7)	5 (33.3)	1 (6.7)	10 (22.2)
Day 180					
Application site discolouration	1	1 (6.7)	0	0	1 (2.2)
Pigmentation	1	1 (6.7)	0	0	1 (2.2)

Data Source: Listing 16.2.7.2, 16.2.7.3
Only Local Injection Site Symptoms were considered, as solicited Adverse Events were summarized.
