# Supplementary material for: Phase 1, randomized, rater and participant blinded placebo-controlled study of the safety, reactogenicity, tolerability and immunogenicity of H1N1 influenza vaccine delivered by VX-103 (a MIMIX microneedle patch [MAP] system) in healthy adults
Source: PLoS One. 2024 Jun 6;19(6):e0303450. doi: 10.1371/journal.pone.0303450 (PMC11156369; doi:10.1371/journal.pone.0303450)
Supplement: S1 Text — (PDF) [file pone.0303450.s006.pdf]

PROTOCOL VX-103-01

A Phase 1, Randomized, Rater and Subject Blinded Placebo Controlled Study to Evaluate the Safety, Reactogenicity, Tolerability and Immunogenicity of a Standard and a Fractional Dose of H1 Influenza Vaccine Delivered by VX-103 (a MIMIX Microneedle Patch (MAP) System) in Healthy Adults ≥18-39 Years of Age

**Investigational product:**	VX-103: Seasonal Influenza Vaccine Delivered by MIMIX Microneedle Array (MAP) System
**Sponsor:**	Vaxess Technologies, Inc.<br>359 Allston Street<br>Cambridge, MA 02139
**Sponsor's Study Director:**	Lynda Tussey, PhD<br>Chief Development Officer<br>Vaxess Technologies, Inc.<br>Phone: 267-253-4528
**Amendment:**	**4.0**
**Version / Date:**	**4.0** / 02-Aug-2022

The information in this study protocol is strictly confidential and is available for review to Investigators, study site personnel, the ethics committee, and the health authorities. It will not be disclosed to third parties without written authorization from the Sponsor, except to obtain informed consent from persons receiving the study treatment. Once the protocol is signed, its terms are binding for all parties.

CONFIDENTIAL

INVESTIGATOR AGREEMENT & STATEMENT OF COMPLIANCE

Table of Contents

LIST OF ABBREVIATIONS **5**

PROTOCOL SYNOPSIS **7**

1. INTRODUCTION **12**

1.1 Background *12*

1.2 Vaccine Design *12*

1.3 Purpose *14*

2 OBJECTIVES **14**

3 STUDY DESIGN **15**

4 RATIONALE **18**

4.1 Rationale for study design *18*

4.2 Rationale for dose/regiment *18*

4.3 Rationale for choice of control product (comparator/placebo) *19*

4.4 Risks and benefits *19*
4.4.1 Blood sample volume 19
4.4.2 Potential risk associated with the COVID-19 Pandemic 19

5 STUDY POPULATION **19**

5.1 Inclusion Criteria *20*

5.2 Exclusion Criteria *20*

6 TREATMENT **22**

6.1 Study Treatment *22*
6.1.1 Investigational and control product 22
6.1.2 Additional Study Treatments 24
6.1.3 Treatment Arms/Groups 24

6.2 Other Treatments *25*
6.2.1 Concomitant Therapy 25
6.2.2 Restriction for study participants 25

6.3 Participant numbering, treatment assignment, randomization *26*
6.3.1 Participant numbering 26
6.3.2 Treatment assignment, randomization 26

6.4 Treatment blinding *26*

6.5 Dose escalation and dose modification *28*

6.6 Additional treatment guidance *28*
6.6.1 Treatment compliance 28
6.6.2 Recommended treatment of adverse events 28
6.6.3 Emergency breaking of assigned treatment codes 29

6.7 Preparation and dispensation 29

7 INFORMED CONSENT PROCEDURES 30

8 VISIT SCHEDULE AND ASSESSMENTS 31

8.1 Screening 35
8.1.1 Eligibility screening 35
8.1.2 Information to be collected on screening failures 35

8.2 Participant demographics/other baseline characteristics 35

8.3 Efficacy 36
8.3.1 Appropriateness of efficacy assessments 36

8.4 Safety, Reactogenicity and Tolerability 36
8.4.1 Laboratory evaluations 37
8.4.3 Pregnancy and assessments of fertility 38
8.4.4 Appropriateness of safety measurements 39

8.5 Additional assessments 39
8.5.1 Immunogenicity 39
8.5.2 Use of residual biological samples 40

9 STUDY DISCONTINUATION AND COMPLETION 40

9.1 Discontinuation and completion 40
9.1.1 Study treatment discontinuation and study discontinuation 40
9.1.2 Withdrawal of informed consent 41
9.1.3 Lost to follow-up 41
9.1.4 Study stopping rules 41
9.1.5 Early study termination by the sponsor 42

9.2 Study completion and post-study treatment 43

10 SAFETY MONITORING AND REPORTING 43

10.1 Definition of adverse events and reporting requirements 43
10.1.1 Adverse events 43
10.1.2 Serious adverse events 44
10.1.3 SAE reporting 45
10.1.4 Pregnancy reporting 46
10.1.5 Reporting of study treatment errors including misuse/abuse 46

10.2 Additional Safety Monitoring 47

11 DATA COLLECTION AND DATABASE MANAGEMENT 47

11.1 Data collection 47

11.2 Database management and quality control 48

11.3 Site monitoring 48

12 DATA ANALYSIS AND STATISTICAL METHODS 49

12.1 Analysis sets 49

12.2 Participant demographics and other baseline characteristics 49

12.3 Treatments 49

12.4 Analysis of the primary endpoint(s) ... 49
12.4.1 Definition of primary endpoint(s) ... 50
12.4.2 Statistical model, hypothesis, and method of analysis ... 50
12.5 Analysis of secondary endpoints ... 50
12.5.1 Efficacy and/or immunogenicity endpoint(s) ... 50
12.5.2 Safety endpoints ... 51
12.5.3 Immunogenicity ... 51
12.6 Analysis of exploratory endpoints ... 51
12.7 Interim analyses ... 52
12.8 Sample size calculation ... 52
13 ETHICAL CONSIDERATIONS AND ADMINISTRATIVE PROCEDURES ... 52
13.1 Regulatory and ethical compliance ... 52
13.2 Responsibilities of the investigator and IRB/IEC ... 52
13.3 Publication of study protocol and results ... 52
13.4 Quality Control and Quality Assurance ... 53
14 PROTOCOL ADHERENCE ... 53
14.1 Protocol amendments ... 53
REFERENCES ... 54
APPENDICES ... 55
Appendix 1: Local Tolerability Scoring ... 55
Appendix 2: Other Local Tolerability Scoring ... 56
Appendix 3: Systemic Toxicity Grading Scales ... 57

Table of Tables

Table 1: Objectives and related endpoints ... 14
Table 2: Randomization Scheme for VX-103 ... 15
Table 3: Investigational and control product ... 22
Table 4: Blinding and Unblinding Plan ... 28
Table 5: Assessment Schedule ... 32
Table 6: Assessment Specification ... 36
Table 7: Clinical laboratory parameters collection plan ... 38
Table 8: Guidance for capturing the study treatment errors including misuse/abuse ... 47

Table of Figures

Figure 1: The MIMIX MAP ... 13
Figure 2: The MIMIX MAP Cartridge ... 14
Figure 3: The MIMIX MAP Applicator ... 14
Figure 4: Overall Study Design ... 18
Figure 5: MIMIX MAP System Use Steps ... 24

LIST OF ABBREVIATIONS

AE	Adverse event
ALT	Alanine aminotransaminase
ALP	Alkaline Phosphate
AST	Aspartate aminotransaminase
BCC	Basal cell carcinoma
BP	Blood pressure
Bpm	Beats per minute
BUN	Blood urea nitrogen
CFR	Code of Federal Regulations
Cm	Centimeters
Cr	Creatinine
CRO	Contract Research Organization
dL	Deciliter
°C	Degrees Celsius
°F	Degrees Fahrenheit
eCRF	Electronic case report form
ELISA	Enzyme-linked immunosorbent assay
FDA	Food and Drug Administration
FSH	Follicle Stimulating Hormone
GCP	Good Clinical Practice
HAI	Hemagglutinin inhibition assay
HBcAb	Hepatitis C virus antibody
HBsAg	Hepatitis B surface antigen
HC	Health Canada
Hgb	Hemoglobin
HIV	Human Immunodeficiency Virus
HR	Heart rate
ICF	Informed consent form
ICH	International Conference on Harmonization
IEC	Independent Ethics Committee
IgE	Immunoglobulin E

IgG	Immunoglobulin G
i.m.	Intramuscular
IRB	Institutional Review Board
IUD	Intrauterine Device
LH	Luteinizing hormone
µg	Microgram
MAP	Microneedle Array Patch
MedDRA	Medical Dictionary for Regulatory Activities
mg	Milligram
mm	Millimeter
mmHg	Millimeter mercury (re: blood pressure)
NYHA	New York Heart Association
PLT	Platelet
PT	Prothrombin Time
PTT	Partial Thromboplastin Time
RR	Rate ratio
SAE	Serious adverse event
SBP	Systolic Blood Pressure
SCC	Squamous cell carcinoma
SGOT	Serum glutamic-oxaloacetic transaminase
SGPT	Serum glutamic pyruvic transaminase
T. Bili	Total Bilirubin
ULN	Upper limit of normal
US	United States
WBC	White Blood Cell
WHO	World Health Organization
WOC	Withdraw of Consent

PROTOCOL SYNOPSIS

**STUDY NUMBER**	VX-103-01
**STUDY TITLE**	A Phase 1, Randomized, Rater and Participant Blinded Placebo Controlled Study to Evaluate the Safety, Reactogenicity, Tolerability and Immunogenicity of a Standard and a Fractional Dose of H1 Influenza Vaccine Delivered by VX-103 (a MIMIX Microneedle Array Patch (MAP) System) in Healthy Adults ≥18-39 Years of Age
**SPONSOR**	Vaxess Technologies, Inc.
**PHASE**	1 (First-Time-In-Humans)
**STUDY POPULATION**	Healthy adult male and non-pregnant female participants aged 18 to 39 years old inclusive
**NUMBER OF SITES**	Up to 3 sites
**STUDY DURATION**	Participants will be screened within a 30-day period prior to receiving a single dose of investigational product or placebo. Participants will have approximately 11 in-person study visits and a study duration of approximately 6 months following dosing.
**INVESTIGATIONAL PRODUCT**	VX-103, a microneedle array patch (MAP) in which the tips (drug product) contain 1% silk fibroin, 0.5% Tween 20 and influenza A H1 A/Guangdong-Maonan/SWL1536/2019 CNIC-1909 antigen influenza antigen (primary drug substance) derived from embryonated hen's eggs (GC Pharma, Republic of Korea).
**CONTROL PRODUCT**	A MAP in which the tips contain 1% silk fibroin, 0.5% Tween 20 (no antigen).
**REGIMEN AND DOSING for VX-103**<br>(Healthy Adults ≥18-39)	A single VX-103 MIMIX MAP immunization at doses of 7.5 µg or 15 µg of GC FLU or placebo to be given by MIMIX MAP to the volar forearm on Study Day 1
**OBJECTIVES**	**Primary:**<br>To describe the safety, reactogenicity and tolerability profiles of VX-103 delivered as a single MIMIX MAP immunization in healthy adults ≥18-39 years of age for two influenza vaccine dose levels, fractional H1 vaccine dose level 7.5 µg and standard H1 vaccine dose level 15 µg vs placebo.<br>**Secondary:**<br>To assess the immunogenicity of VX-103 vaccine delivered in a single MIMIX MAP dose regimen in healthy adults in ≥18-39 years of age.<br>To assess VX-103 delivery as a dose sparing skin immunization strategy by comparing the safety, reactogenicity, tolerability and immunogenicity of a fractional H1 vaccine dose level (7.5 µg) delivered by MIMIX MAP to a standard H1 vaccine dose level (15 µg) delivered by MIMIX MAP.
**STUDY DESIGN**	A rater and participant-blinded, randomized, placebo (no antigen)-controlled study to describe the safety, reactogenicity, tolerability and immunogenicity of VX-103 vaccine dose levels, *e.g.*, 7.5 µg and 15 µg of investigational product, delivered as a single dose on Study Day 1 intradermally via MIMIX MAP skin immunization, compared to placebo.<br>A maximum of 45 healthy adults ≥18-39 years of age will be centrally randomized in a 1:1:1 ratio among three (3) groups. Each participant will receive the either the standard dose (15 µg), a fractional dose (7.5 µg) of the H1 influenza antigen, or placebo (no antigen) delivered via MIMIX MAP to the volar forearm.

<table>
<tr><td></td><td>Each participant will remain at the study site for at least one hour (+30 minutes) to be observed for any immediate reactogenicity complaints associated with the Day 1 treatment.

Group	N	Day 1 Observer blind IP or Placebo administered
		MIMIX MAP Dose
1	15	15 µg
2	15	7.5 µg
3	15	Placebo

Participants will be followed for up to 180 days post immunization and evaluated during clinic visits on Study Days 2 (+1), 4 (+1), 8 (±2), 15 (±2), 29 (±2), 57 (±2), 85 (±7), 119 (±7), (final end of study visit) 180 (±7) following vaccination.

Participants will be contacted telephonically when an in-person visit is not scheduled through 8 days after treatment and will be assessed for solicited systemic and local adverse reactions and adverse events (AE) through Study Day 8, and solicited erythema and pigmented coloration on Study Days 15, 29, 57 and 180. Unsolicited AEs will be assessed from Study Days 1 through Day 29 after vaccination. Photographs of the treatment sites will be taken at each study visit through Day 57 and then at the end of study visit (Day 180).</td></tr>
<tr><td>SAMPLE SIZE</td><td>Approximately 45 young male and non-pregnant female adult volunteers will be enrolled.</td></tr>
<tr><td>KEY INCLUSION/ EXCLUSION CRITERIA
(Healthy Adults ≥18-39)</td><td><u>KEY INCLUSION</u>
• Male or female aged 18 – 39 years inclusive
• Provide written informed consent to participate
• Healthy participants without acute or chronic, clinically significant pulmonary, cardiovascular, hepatic or renal functional abnormality
  ○ As determined by medical history, physical exam, laboratory screening
• Body Mass Index 18-35 kg/m2, inclusive, at screening
• Females should fulfill one of the following criteria:
  ○ At least one year post-menopausal,
  ○ Surgically sterile,
  ○ Will use oral, implantable, transdermal or injectable contraceptives during the screening window and until 60 days after vaccination
  ○ Willing to use a form of highly effective contraception approved by the Investigator (e.g., intrauterine device (IUD), female condom, diaphragm with spermicide, cervical cap, use of condom by the sexual partner or a sterile sexual partner) for study duration and until 60 days after vaccination.
• Women of childbearing potential must have a negative urine pregnancy test at screening and within 24 hours preceding receipt of vaccination
• Male participants must be surgically sterile (e.g., vasectomy) or agree to practice highly effective contraception from the vaccination until 60 days after vaccination.
<u>KEY EXCLUSION CRITERIA</u>
• Any medical condition that in the judgement of the investigator would make subject participation in the study unsafe.
• Having cancer or received treatment for cancer within three years (persons with a history of cancer who are disease-free without treatment for three years or more are eligible), excluding basal cell carcinoma (BCC) or squamous cell carcinoma (SCC), which are allowed unless located at the vaccination site.
• Impaired immune responsiveness (of any cause), including diabetes mellitus.</td></tr>
</table>

	• Receipt or plan to receive a non-study vaccine within 30 days prior to vaccination or 60 days after vaccination.<br>• Receipt of any influenza vaccine in previous 24 months and/or planned receipt of influenza vaccine for the duration of the study.<br>• Diagnosed influenza infection in the previous 24 months prior to screening.<br>• Diagnosed COVID infection via medical personnel or at home test within the past 60 days prior to screening<br>• Female participant who is pregnant or breastfeeding.<br>• A tattoo, scar or excessive hair in volar forearm region which would impede assessment of MAP application site<br>• Allergy to influenza vaccine or components, or history of severe local or systemic reaction to any vaccination.<br>• History of anaphylactic type reaction to injected vaccines<br>• History of or current allergy to latex<br>• History of Guillain-Barré Syndrome.<br>• Positive test result for hepatitis B surface antigen (HBsAg,), hepatitis C virus antibody (HBcAb), or human immunodeficiency virus (HIV) types 1 or 2 antibodies at screening.<br>• History of chronic obstructive pulmonary disease or history of other lung disease.<br>• History of severe allergic reactions to eggs.
**STUDY SCHEDULE**	There will be a Screening window that is up to 30 days where all necessary assessments will be conducted to determine eligibility prior to randomization on study Day 1. Investigational product (test article) administration will occur on Day 1 for each randomized subject and will be given by an unblinded administrator.<br>In-clinic safety evaluations will be performed at screening, at Day 1 before and after administration of the test articles, and at Days 2 (+1), 4 (±1), 8 (±2), 15 (±2), 29 (± 2), 57 (± 2), 85 (± 7), 119 (± 7) and 180 (± 7).<br>Telephone calls from the site to the participant will be made on Days 5, 6 and 7.<br>Photographs of the MIMIX MAP application site will be taken on the day of administration (pre-dose, then 1 (±2), 30 (±5) and 60 (±5) minutes post-MAP removal), and on Days 2 (+1), 4 (±1), 8 (±2), 15 (±2), 29 (± 2) 57 (±2) and180 (±7).<br>Specimens for assessment of immune response will be collected at screening, before first vaccination on Day 1, and on Days 29 (±2), 57 (+2), 85 (± 7), 119 (± 7) and 180 (± 7).<br>**A detailed schedule of assessments appears in** Table 5**.**
**SAFETY ASSESSMENTS**	On day of vaccination, observe study participants in clinic for a minimum of 1 hour (+ 30 minutes) post-dose as directed by the study physician or designate.<br>Solicitation of local and systemic reactogenicity events will be captured via clinic visits as applicable using standardized grading. Reactogenicity assessment will also include unsolicited complaints.<br>All local and systemic reactogenicity reporting in the 7 days after immunization will be supported via in-person visits and telephonically. Calls are to query for any adverse events using, complete Appendix 1, 2 and the systemic section of Appendix 3, and to determine if an on-site visit is needed.<br>Skin tolerability assessments including pain, induration, tenderness, erythema and edema (Appendix 1: Local Tolerability Scoring) will be carried out on the day of administration (pre-dose, then 1 (±2), 30 (±5) and 60 (±5) minutes post-MAP removal), and on Days 2 (+1), 4 (+1), 8 (±2). Erythema assessments only will be carried out on Days 15 (±2), 29 (± 2), 57(±2) and 180 (±7).<br>Other local tolerability assessments of the MAP application site including bruising, itching, skin flaking and pigmented skin coloration (Appendix 2: Other Local Tolerability Scoring) will be carried out on the day of administration (pre-dose and 60 (±5) minutes post-MAP removal), and on Days 2 (+1), 4 (±1), 8 (±2). Pigmented coloration assessments only will be carried out on Days 15 (±2), 29 (± 2), 57 (±7) and180 (±7).

<table>
<tr><td></td><td>Clinic visit on Days 2 (+1), 4 (+1), 8 (±2), 15 (±2), 29 (± 2), 57 (± 2), 85 (±7), 119 (± 7) and 180 (± 7) will assess adverse events, concomitant medications, and clinical labs.</td></tr>
<tr><td>IMMUNOGENICITY ASSESSMENTS</td><td>Serum hemagglutination-inhibition (HAI) titers will be assessed on pre-vaccination on Day 1, and on Days 29 (±2), 57 (±2), 85 (± 7), 119 (±7) and 180 (± 7) after vaccination. Anti-HA, virus microneutralization titers, anti-silk fibroin Immunoglobulin G (IgG) and total IgE titers will be assessed on pre-vaccination on Day 1, and on Days 29 (± 2), and 180 (± 7) after vaccination.</td></tr>
<tr><td>HALTING RULES</td><td>
<table>
<tr><th>Event</th><th>Number of Participants</th></tr>
<tr><td>Death or any life-threatening serious AE (SAE)</td><td>1</td></tr>
<tr><td>Any SAE that cannot reasonably be attributed to a cause other than vaccination</td><td>1</td></tr>
<tr><td>Any withdrawal from the study or withdrawal from study vaccine(s)/product(s) (by investigator or subject request) following a grade 3 AE that cannot reasonably be attributed to a cause other than vaccination</td><td>1</td></tr>
<tr><td>Any local or general solicited AE leading to hospitalization, or fever > 40°C or 104°F (oral route) that cannot reasonably be attributed to a cause other than vaccination, or necrosis at the injection site, within the 7-day (Days 1-7) post-vaccination period</td><td>1</td></tr>
<tr><td>Any grade 3 solicited local AE lasting 48h or more in an investigational group, within the 7-day (Days 1-7) post-vaccination period</td><td>1</td></tr>
<tr><td>Any grade 3 solicited general AE lasting 48 hours or more in an investigational group, that cannot reasonably be attributed to a cause other than vaccination, within the 7- day (Days 1-7) post-vaccination period</td><td>1</td></tr>
<tr><td>Any grade 3 unsolicited AE in an investigational group, that cannot reasonably be attributed to a cause other than vaccination, within the 7-day (Days 1-7) postvaccination period OR Any grade 3 abnormality in the same pre-specified hematological or biochemical laboratory parameters in an investigational group, that cannot reasonably be attributed to a cause other than vaccination, within the 7 days post-vaccination</td><td>1</td></tr>
</table>
</td></tr>
<tr><td>FOLLOW-UP DURATION</td><td>Participants will be followed for up to 180 days (± 7) after vaccination.</td></tr>
<tr><td>ENDPOINT PARAMETERS</td><td>a) Safety analysis will be identified within the Statistical Analysis Plan and includes:</td></tr>
</table>

	○ Standard descriptive demography. All participants who receive any investigational product will be included in the safety analyses.<br>○ Proportion of participants in each treatment group with any non-zero report will be tabulated for each local and systemic solicited and unsolicited reactogenicity event; groups will be compared by Fisher's exact test (alternatively, a Cochran-Mantel-Haenszel [CMH] test will be used to analyze events by ordinal severity category).<br>○ Participants with adverse events (including clinical laboratory abnormalities) will be summarized by Medical Dictionary for Regulatory Activities (MedDRA) body organ system and preferred term, severity, relatedness and, separately, by seriousness.<br>○ Proportion of participants in each treatment group with AE reports within each body organ system will be compared in the same manner. Significant heterogeneity will be probed at the preferred term level.<br>b) Immunogenicity Parameters<br>○ Sera collected will be analyzed for determination of HAI titers. Serum IgG anti-H1 HA, virus microneutralization titers, serum IgE and anti-silk fibroin by Enzyme-linked immunosorbent assay (ELISA) will be assessed on (vaccination day), and on Days 29 (±1), and 180 (± 5).<br><br>The results of these determinations will be evaluated as follows:<br><br>• Calculation of the geometric mean of pre- and post-vaccination HAI and anti-H1 HAI IgG serum antibody titers for each treatment group.<br>• Change from pre- to post-vaccination in geometric mean HAI and anti-H1 HAI IgG titers for each treatment group.<br>• Seroconversion rate defined as percentage of participants with either a pre-vaccination HAI titer <1:10 and post vaccination HAI titer of ≥ 1:40 or a pre-vaccination HAI titer of ≥ 1:10 and a minimum four-fold rise in post vaccination HAI antibody titer at each timepoint.<br>• Seroprotection rate defined as the percentage of participants with a HAI titer ≥ 1:40 at each timepoint<br>• Calculation of the geometric mean of pre- and post-vaccination influenza virus microneutralization titers<br>• Change from pre- to post-vaccination in geometric mean microneutralization titers<br>• Calculation of the geometric mean of pre- and post-vaccination anti-silk fibroin serum IgG antibody titers for each treatment group.<br>• Change from pre- to post-vaccination in geometric mean anti-silk fibroin IgG titers<br>• Calculation of the geometric mean of pre- and post-vaccination total serum IgE titers<br>• Change from pre-to post-vaccination in geometric mean total IgE titers

1. INTRODUCTION

1.1 Background

Although influenza vaccines reduce the risk of flu illness by between 40% and 60% among the overall population, factors such as age and the degree of antigenic match are known to affect their effectiveness (Allen & Ross, 2018; Belongia et al., 2016; Darvishian et al., 2014; Rondy et al., 2017).

A recently emerging factor affecting influenza vaccine effectiveness is waning immunity within the influenza season. Although, several studies have reported decreases in vaccine effectiveness over the influenza season, waning effects have not been observed consistently across age groups, virus subtypes, and seasons. Still, the emerging data has prompted the Center for Disease Control (CDC) to provide a timing recommendation for receipt of the influenza vaccine along with the suggestion that getting vaccinated early (in July or August) is likely to be associated with reduced protection later in the flu season (CDC, 2020). A recent study has suggested that the waning immunity may be linked to the rapid decline of bone marrow plasma cells (which correlate with circulating levels of antibody secreting cells) that are responsible for the long-term maintenance of serum antibody levels (Davis et al., 2020).

There has been a long-standing interest in intradermal influenza vaccines because of their dose-sparing potential and therefore their ability to mitigate potential vaccine shortages, which could occur from unanticipated loss of expected supplies or from excessive demand owing to high rates of infection, such as during pandemics (Kenney et al., 2004; La Montagne & Fauci, 2004). A recent meta-analysis conducted (Egunsola et al., 2021) reported no statistically significant difference in seroconversion rates between the 3-µg, 6-µg, 7.5-µg, and 9-µg intradermal vaccine doses and the 15-µg intramuscular vaccine dose for each of the H1N1, H3N2, and B strains, but rates were significantly higher with the 15-µg intradermal dose compared with the 15-µg intramuscular dose for the H1N1 strain (rate ratio [RR], 1.10; 95% CI, 1.01-1.20) and B strain (RR, 1.40; 95% CI, 1.13-1.73). Thus, intradermal delivery has the potential not only for dose sparing but also to improve immune responses to influenza vaccines.

In summary, while current influenza vaccines are safe and offer moderate protection against influenza associated disease, unmet needs remain. There is a current need for vaccine platforms that can improve vaccine effectiveness, extend the durability of the immune response while also providing dose sparing capabilities.

1.2 Vaccine Design

The MIMIX MAP is a novel intradermal MAP vaccination system comprising microneedles of silk fibroin and vaccine antigen designed for prolonged release of antigen into the skin, the largest immune organ in the body. Sustained antigen presentation is known to modulate the magnitude, duration and specificity of immune responses. In mouse models of MIMIX

vaccination we have observed sustained stimulation of germinal center responses, trends towards an improvement in the magnitude of HAI titers particularly at timepoints more than 1 month post dosing, a broadening in immunity against drifted influenza strains, and stronger antigen-specific T cell responses than observed with bolus intramuscular (IM) delivery (Stinson et al., 2021). These attributes have the potential to address some of the shortcomings of current licensed influenza vaccines.

The MIMIX MAP includes the Microneedle Array and a Backing that comprises an Adhesive, and a structural Plastic Film as shown in Figure 1. The Microneedle Array is made of up of a regular pattern of microneedles that include the antigen containing Tip (DP) and a structural base. Each MIMIX MAP is produced by an additive manufacturing approach using a silicon mold that defines the microneedle shape. The MAP is separated from the silicon mold through assembly to the Backing.

Figure 1: The MIMIX MAP

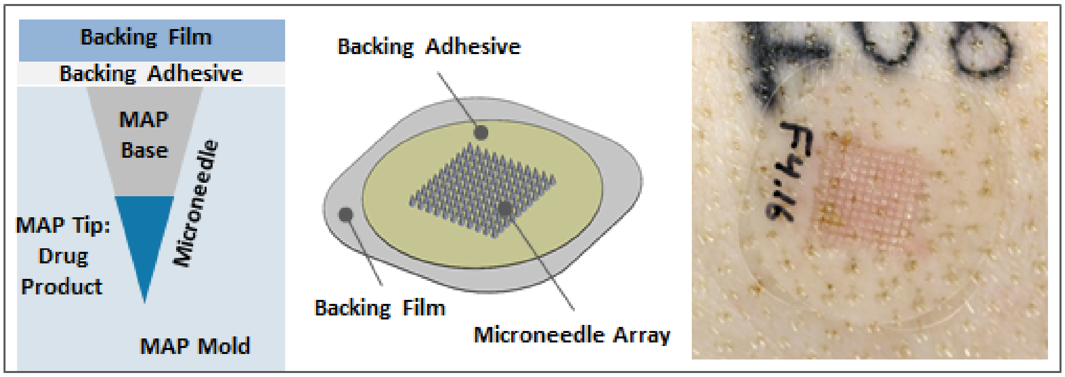

After separation from the silicon mold the MAP is packaged in a Carrier with a Retainer to form the MAP Cartridge; a single-use, disposable subsystem that consists of the MAP, a Carrier, and Retainer (Figure 2). The Carrier is designed to hold the MAP and mediates mounting to a spring-loaded Applicator. The Retainer secures the MAP in the Carrier. The MAP Cartridge is encased in a polystyrene Tray and inserted in a foil lined pouch with a desiccant for shipping and storage at 4°C. The MAP Cartridge is the only part of VX-103 that contacts the participant.

Figure 2: The MIMIX MAP Cartridge

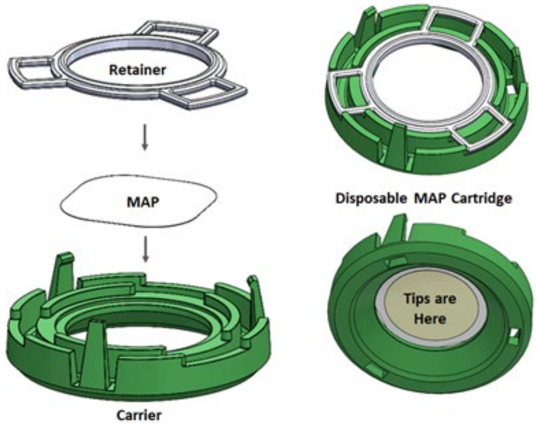

To facilitate consistent MAP application, Vaxess has developed a single use, spring-loaded Applicator (Figure 3). Snap hooks on the Carrier component of the MAP Cartridge allow for mounting of the Cartridge to the Applicator. The Applicator utilizes a spring-loaded piston to generate downward force on the MAP during delivery. Each Applicator will be individually packaged and provided to the Clinical sites to support MAP application.

Figure 3: The MIMIX MAP Applicator

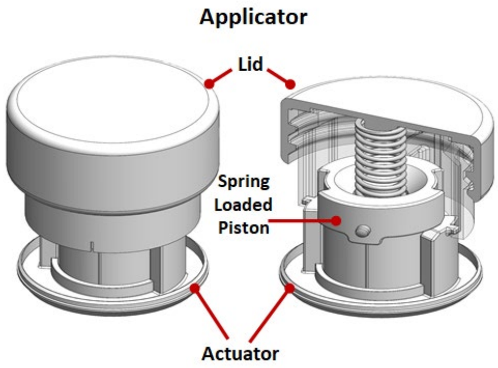

1.3 Purpose

The purpose of this study is to generate the necessary safety, reactogenicity, and tolerability information that will support further clinical development of VX-103 delivered as MIMIX MAP for seasonal influenza.

2 OBJECTIVES

Table 1: Objectives and related endpoints

Objective(s)	Endpoint(s)
**Primary objective(s)**	**Endpoint(s) for primary objective(s)**
• To describe the safety, reactogenicity and tolerability profiles of VX-103	• Changes in physical exams, vital signs, laboratory parameters of hematology, clinical chemistry, urinalysis,

delivered as a single MIMIX MAP immunization in healthy adults ≥18-39 years of age for two influenza vaccine dose levels, fractional H1 vaccine dose level 7.5 µg and standard H1 vaccine dose level 15 µg vs placebo.	AEs, and local and systemic solicited and unsolicited reactogenicity event(s)
**Secondary objective(s)**	**Endpoint(s) for secondary objective(s)**
• To assess the immunogenicity of VX-103 delivered in a single MIMIX MAP dose regimen in healthy adults in ≥18-39 years of age.<br>• To assess VX-103 MIMIX MAP delivery as a dose sparing skin immunization strategy by comparing the safety, reactogenicity, tolerability and immunogenicity of a fractional H1 vaccine dose level (7.5 µg) delivered by MIMIX MAP to a standard H1 vaccine dose level (15 µg) delivered by MIMIX MAP.	• Influenza A H1 geometric mean HAI titer; mean fold rise and seroconversion rates for each treatment group and timepoint on Days 1, 29, 57, 85, 119, 180.<br>• MIMIX MAP dose level comparisons in vital signs, laboratory parameters of hematology, clinical chemistry, urinalysis, adverse events, and local and systemic solicited and unsolicited reactogenicity event(s)<br>• Influenza A H1 geometric mean HAI titer; mean fold rise and seroconversion rates for each route and timepoint for the fractional dose versus full dose delivered by MIMIX
**Exploratory objective(s)**	**Endpoint(s) for exploratory objective(s)**
• To assess seroprotection rates, anti-H1 HA IgG, virus microneutralizing (MN) titers, anti-fibroin IgG and total IgE titers to the influenza vaccine	• Influenza A H1 seroprotection rate for each treatment group on Days 1, 29, 180 by HAI and anti-influenza A H1 HA GMT, virus MN titers, anti-fibroin IgG, and total IgE for each treatment group on Days 1, 29, 180
• To evaluate the durability of the immune response for each vaccine dose level	• Influenza A H1 seroconversion rate, GMT, fold increase in GMT and SPR by HAI on Days 29 and 180 for treatment group
• To assess, in at least a subset of samples, the breadth of the influenza A H1 antigen responses	• HAI titers against a panel of drifted H1 strains Days 1, 29, 180

3 STUDY DESIGN

This is an exploratory, FIH, randomized, rater and participant-blinded, placebo-controlled study in 45 healthy participants. This study will be carried out in healthy adult (18 to 39 years of age, inclusive) participants where each participant will receive either the standard dose (15 µg), a fractional dose (7.5 µg) of the H1 influenza antigen, or placebo (no antigen) delivered via MIMIX MAP skin to the volar forearm. The groups will be randomized in a 1:1:1 ratio among the three groups (15 µg, 7.5 µg, and placebo, see Table 2).

Table 2: Randomization Scheme for VX-103

Group	N	Day 1 Observer blind IP or Placebo administered
		**MIMIX MAP Dose**
1	15	15 µg
2	15	7.5 µg
3	15	Placebo

There will be up to a 30-day screening period (Day ±30 to Day 0) to assess eligibility. Eligible participants will be admitted into the study clinic on Day 1 and if eligible, randomized on Day 1. All participants will be followed for safety with the following assessments as outlined in the Assessment Schedule in Table 5.

Prior to treatment, blood will be collected for safety assessments and measurement of immune response (baseline at Day 1) and vital signs collected.

Prior to treatment, the MAP administration site will be identified, examined and marked with indelible ink and photographed using a study specific iPhone and a DermLite imaging system or similar system. Imaging instructions will be outlined in a separate document. All sites will use the same system.

The process for using the applicator and applying the MAP will be described to all participants before treatment.

After treatment, the used MAP backing will be discarded in a biohazard waste container while the used MAP Cartridges and Applicators will be returned to Vaxess Technologies at the conclusion of the trial. Any Applicator malfunctions will be documented on site source documents and captured within the EDC.

Treatment sites will be examined/assessed and photographed after MAP delivery as set out in the Assessment Schedule (Table 5).

Following MAP administration on Day 1, post-dose assessments will include Skin Tolerability Scores (Appendix 1: Local Tolerability Scoring) 1 (±2), 30 (±5) and 60 (±5) minutes post-MAP removal, Other Local Tolerability assessments of the MAP application site (Appendix 2: Other Local Tolerability Scoring) 60 (±5) minutes post-MAP removal, iPhone photographs 1 (±2), 30 (±5) and 60 (±5) minutes, DermLite attached to iPhone photographs 60 (±5) minutes post-MAP removal and vital signs. Participants will be discharged from the unit after all assessments have been completed and a 1-hour observation period has elapsed.

On Day 2 (24 hr post treatment), participants will return to the clinic for an abbreviated physical exam, vital sign measurements, a review of any systemic or local site reactions (skin tolerability and other local tolerability assessments) and for further application site photographs to be taken.

On Day 4 (3 days post treatment), participants will return to the clinic for an abbreviated physical exam, vital sign measurements, a review of any systemic or local site reactions (skin tolerability and other local tolerability assessments) and for further application site photographs to be taken. Blood samples will be collected for hematology and chemistry.

On Day 5, 6 and 7 a phone call will be made to participants to ask about spontaneous adverse events or changes on concurrent medication.

On Day 8 (+/- 1 day) participants will return to the clinic for an abbreviated physical exam, vital sign measurements, a review of any systemic or local site reactions (skin tolerability and other local tolerability assessments) and for further application site photographs to be taken.

On Day 15 (+/- 2 day), participants will return to the clinic for an abbreviated physical exam, vital sign measurements, for erythema (Appendix 1: Local Tolerability Scoring) only and pigmented coloration assessments (Appendix 2: Other Local Tolerability Scoring) only and for further application site photographs to be taken.

On Day 29 (+/- 2 day) participants will return to the clinic for an abbreviated physical exam, vital sign measurements, for erythema (Appendix 1: Local Tolerability Scoring) only and pigmented coloration assessments (Appendix 2: Other Local Tolerability Scoring) only and for further application site photographs to be taken. Blood samples will be collected for hematology, biochemistry and immunogenicity analysis.

On Day 57 (+/- 4 days), participants will return to the clinic for additional application site photographs, an abbreviated physical exam, vital sign measurements and for erythema (Appendix 1: Local Tolerability Scoring) only and pigmented coloration assessments (Appendix 2: Other Local Tolerability Scoring) only. Blood samples will be collected for immunogenicity analysis.

On Day 85 (+/- 7 days), participants will return to the clinic for an abbreviated physical exam and vital sign measurements. Blood samples will be collected for immunogenicity analysis.

On Day 119 (+/- 7 days), participants will return to the clinic for an abbreviated physical exam and vital sign measurements. Blood samples will be collected for immunogenicity analysis.

On Day 180 (+/- 7 day) participants will return to the clinic for an abbreviated physical exam, vital sign measurements, a review of erythema (Appendix 1: Local Tolerability Scoring) only and pigmented coloration assessments (Appendix 2: Other Local Tolerability Scoring) and for further application site photographs to be taken. Blood samples will be collected for hematology, chemistry and immunogenicity analysis.

Procedures at all visits will be performed according to the Assessment Schedule (Table 5).

Participants will be informed to contact the clinic during the course of the study if they have any concerns at all regarding their health and wellbeing.

If required by the Investigator, participants from any of the treatment groups may attend the clinical unit for unscheduled visits to allow assessment and treatment of any adverse events they may experience.

The post-treatment follow-up after vaccine administration will be approximately 180 days (Figure 4).

Figure 4: Overall Study Design

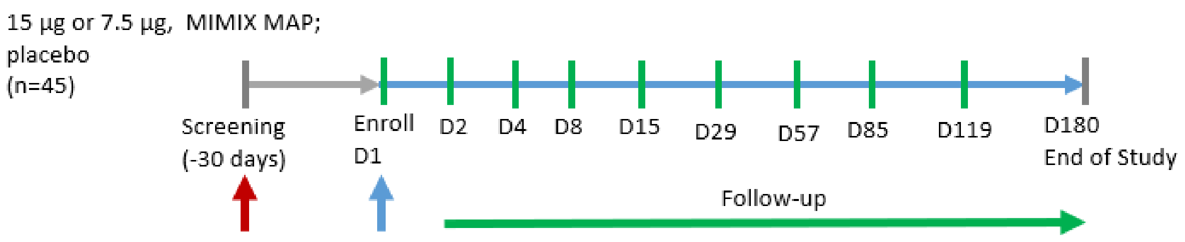

Each participants' study duration will be approximately 7.5 months (222 days) (Table 5) accordingly.

4 RATIONALE

4.1 Rationale for study design

This is a standard FIH study design. The study will use a rater and participant-blinding, a placebo-controlled design and a single dose regimen.

- **Healthy participants:** The absence of co-morbid disease in healthy participants allows for an unbiased assessment of the safety and tolerability
- **Blinding:** Blinding allows for an unbiased assessment of readouts such as adverse events
- **Dietary restrictions:** Food restriction from at least 4 hours prior to dosing until after completion of the post dose assessments is implemented to avoid effects of food on assessments.
- **Randomization:** Will be performed via a randomization plan**.** This decreases the chance of an imbalance in participant characteristics between groups, thereby facilitating and unbiased assessment of safety, reactogenicity and tolerability
- **Inpatient for dosing:** This allows for close observation of safety, reactogenicity and tolerability, and ensures compliance with instructions for all participants.

4.2 Rationale for dose/regiment

Vaxess will utilize a bulk concentrate of the A/H1N1 virus antigen components (GC Pharma) recommended for the 2020-2021 northern hemisphere (NH) influenza season. The finished vaccine (commercialized as "GC-Flu") that incorporates this bulk is a split-virus, formalin-inactivated trivalent (or, more recently, quadrivalent) influenza vaccine produced in embryonated eggs. GC-Flu is not licensed in the US or Canada but has consistently met the requirements of the European Pharmacopoeia and the World Health Organization (WHO) since 2009. The standard dose per component of GC-Flu (and other licensed seasonal influenza vaccines) is 15 µg per strain. Thus, Vaxess has selected the standard influenza vaccine dose as one of the dose levels for evaluation. The fractional dose of 7.5 µg was selected as a dose level that has been demonstrated to have comparable immunogenicity to 15 µg and therefore has the potential to be dose sparing (Song et al., 2013).

4.3 Rationale for choice of control product (comparator/placebo)

This study is a safety, reactogenicity and tolerability study conducted in healthy participants, and in the frame of a first administration in human, placebo is the most appropriate comparator for the objectives of the study.

4.4 Risks and benefits

There is no benefit expected for participants in this study.

The risk to participants in this trial may be minimized by compliance with the eligibility criteria and study procedures, as well as close clinical monitoring, and as applicable, inpatient status, stopping rules, minimal study duration, periodic review of safety data by sponsor and investigator. Refer to the Investigator's Brochure (IB) for additional information.

The investigational drug will be prepared by a trained pharmacy staff according to the study specific procedure included in the pharmacy manual and administered by a trained study team member in order to comply with the unblinded/blinded roles.

4.4.1 Blood sample volume

The anticipated maximum blood volume collected from participants is approximately 85 mL. Additional samples may be required for additional safety monitoring.

Timings of blood sample collection are outlined in the assessment schedule (Table 5).

See Section 8.5.2 Use of residual biological samples for information about the use of residual samples.

4.4.2 Potential risk associated with the COVID-19 Pandemic

Vaxess is committed to supporting the safety and well-being of our study participants, investigators, and site staff. All local regulations and site requirements are being applied in the countries that are affected by the COVID-19 pandemic, including COVID-19 testing of participants if applicable. The Vaxess team will review the situation in each participating country and work with investigators to continue to ensure the safety of participants during the conduct of the trial. As the COVID-19 situation evolves, investigators must use their best judgement to minimize risk to participants during the conduct of the study.

5 STUDY POPULATION

The study population comprises healthy male and female participants. A total of approximately 45 healthy adult participants will be enrolled in the study and randomized at one-three study sites.

Replacement participants will be enrolled to replace participants who discontinue the study as outlined in Section 9.1.1.1 Replacement policy. Participants who fail initial screening will not be allowed to rescreen.

The investigator must ensure that all participants being considered for the study meet all of the inclusion and none of the exclusion criteria prior to enrollment.

Each inclusion and exclusion criterion specifies if the criterion should be assessed at screening and/or baseline (pre-vaccination on Day 1). A relevant record (e.g. checklist) of the eligibility criteria must be stored with the source documentation at the study site.

Deviation from any entry criterion excludes a participant from randomization into the study.

5.1 Inclusion Criteria

1. Male or female aged 18 – 39 years, inclusive at screening.
2. Provide written informed consent to participate in the trial.
3. Participants who, in the opinion of the investigator, can and will comply with the requirements of the protocol.
4. Healthy participants without acute or chronic, clinically significant pulmonary, cardiovascular, hepatic or renal functional abnormality
    a. as determined by medical history, physical exam, laboratory screening
5. Body Mass Index 18-35 kg/m2, inclusive, at screening.
6. Females should fulfill one of the following criteria:
    - At least one year post-menopausal,
    - Surgically sterile,
    - Will use oral, implantable, transdermal or injectable contraceptives during the screening window and until 60 days after vaccination
    - Willing to use a highly effective method of contraception approved by the Investigator for study duration and until 60 days after vaccination
7. Women of childbearing potential must have a negative urine pregnancy test at screening and within 24 hours preceding receipt of vaccination.
8. Male participants must be surgically sterile (e.g., vasectomy) or agree to practice highly effective method of contraception from screening until 60 days after vaccination.
9. Vital signs within following range:
    a. oral temperature <100.0°F (37.8°C),
    b. pulse <100 bpm,
    c. Systolic blood pressure (SBP) ≤ 150 to 85 mmHg
10. Clinical screening laboratory evaluations (White Blood Cells [WBCs], hemoglobin [Hgb], platelets [PLTs], Alanine Transaminase [ALT], Aspartate Transaminase [AST], Creatinine [Cr], Alkaline Phosphatase [ALP], Total Bilirubin [T. Bili], Lipase, Prothrombin Time [PT], Partial Thromboplastin Time [PTT]) are within acceptable normal reference ranges at the clinical laboratory being used or determined to not be clinically significant in the opinion of the investigator.

5.2 Exclusion Criteria

1. Any medical condition that in the judgement of the investigator would make the subject participation in the study unsafe.
2. Any condition, including confirmed or suspected immunosuppressive or immunodeficient condition, that in the judgement of the investigator would make it unsafe for the subject to participate.

3. History of or current autoimmune disease or impaired immune responsiveness (of any cause).
4. Having cancer or received treatment for cancer within three years (persons with a history of cancer who are disease-free without treatment for three years or more are eligible), excluding basal cell carcinoma (BCC) or squamous cell carcinoma (SCC), which are allowed unless located at the vaccination site.
5. Impaired immune responsiveness (of any cause), including diabetes mellitus.
6. Presently receiving or having a recent history of receiving (within the past six months) any medication or therapeutic modality that affects the immune system such as allergy shots, immune globulin, interferon, immunomodulators, radiation therapy, cytotoxic drugs or drugs known to be frequently associated with significant major organ toxicity, or systemic corticosteroids (oral or injectable). Inhaled and topical corticosteroids are allowed.
7. Receipt or plan to receive a non-study vaccine within 30 days prior to VX-103-01 vaccination or 60 days after vaccination.
8. Receipt of any influenza vaccine in previous 24 months and/or planned receipt of influenza vaccine through the duration of the study.
9. Diagnoses influenza infection in the previous 24 months prior to screening.
10. Diagnosed COVID infection via medical personnel or at home test within the past 60 days prior to screening.
11. Female participant who is pregnant or breastfeeding.
12. A tattoo, scar or excessive hair in volar forearm region which would impede assessment of MAP application site.
13. Allergy to influenza vaccine or components, or history of severe local or systemic reaction to any vaccination.
14. History of anaphylactic type reaction to injected vaccines
15. History of or current allergy to latex.
16. History of Guillain-Barré Syndrome.
17. Receipt of any investigational product or nonregistered drug within 30 days prior to vaccination or currently enrolled in any investigational drug study or intends to enroll in such a study within the study period.
18. Receipt of blood or blood products 8 weeks prior to vaccination or planned administration during the study period.
19. Donation of blood or blood products within 8 weeks prior to screening or at any time during the study.
20. Acute disease and/or fever (≥ 38.0°C / 100.4°F via oral cavity) within 72 hours prior enrollment.
21. Positive test result for HBsAg, HBcAb, or HIV types 1 or 2 antibodies at screening.
22. Diagnosed eczema, atopic dermatitis or other inflammatory skin disease.
23. Significant cardiovascular disease e.g., NYHA Class 3 or 4 congestive heart failure; myocardial infarction within the past six months; unstable angina, coronary angioplasty within the past six months; uncontrolled ventricular cardiac arrhythmias; resting HR >100 bpm

24. History of chronic obstructive pulmonary disease or history of other lung disease.
25. History of severe allergic reactions to eggs.
26. Use of illicit and recreational drugs throughout the trial (i.e. cocaine, amphetamines, etc.) is prohibited. The use of Marijuana is only prohibited during the 7 days prior to enrollment until after Study Day 8.
27. Diagnosis of or family history of Keloid Disease
28. History of drug or chemical abuse in the year prior to screening.
29. Consumption of alcohol for 48 hours prior to enrollment and for 24 hours after enrollment.

6 TREATMENT

6.1 Study Treatment

Details on the requirements for storage and management of study treatment, and instructions to be followed for participant numbering, dispensing, and taking study treatment are outlined in the pharmacy manual.

The MIMIX MAP application will be administered by MIMIX skin immunization to the volar forearm on Study Day 1.

All blood samples will be taken from the veins of the contralateral arm of immunization.

Refer to the 'dietary restrictions and smoking' 6.2.2.2 Dietary restrictions for details of dosing and food intake, if relevant.

6.1.1 Investigational and control product

The investigational product, VX-103 and the placebo will be prepared by Vaxess and supplied as open-labeled product to the unblinded site pharmacist (Table 3). The single use, spring-loaded applicator will be packaged and provided separately.

Table 3: Investigational and control product

Investigational/ Control Product (Name and Strength)	Dosage Form	Route of Administration	Supply Type	Sponsor
VX-103 7.5 µg.	Microneedle array patch (MAP) with tips containing 1% silk fibroin, 0.5% Tween 20 and influenza A H1 A/Guangdong-Maonan/SWL1536/2019 CNIC-1909 antigen influenza antigens	Intradermal, skin immunization	Open label bulk supply; patches	Sponsor
VX-103 15 µg	Microneedle array patch (MAP) with tips containing 1% silk fibroin, 0.5% Tween 20 and influenza A H1 A/Guangdong-Maonan/SWL1536/2019 CNIC-1909 antigen influenza antigens	Intradermal, skin Immunization	Open label bulk supply; patches	Sponsor

Matching Placebo	Microneedle array patch (MAP) with tips containing 1% silk fibroin, 0.5% Tween 20 and no antigen	Intradermal, skin immunization	Open label bulk supply; patches	Sponsor

An unblinded pharmacist or authorized designee is required to prepare the study product.

The MIMIX MAP will be administered to the volar forearm on Study Day 1 by study personnel that have the appropriate training and are listed on the Delegation of Authority log. Prior to treatment, the forearm region of the arm must be examined, marked with indelible ink, photographed and cleaned. For the volar forearm, the site of the application must be an area of skin between ~ 4 and 8 cm below the elbow joint.

Application sites will be selected to be free from sores, scarring, blemishes, moles, tattoos, skin conditions, sunburn, heavy hair, redness or coloration which might interfere with the detection of local reactions. If possible, the non-dominant arm of the subject should be used for administration of treatment. Please refer to the VX-103-01 Instructions for Use for additional detail.

All MAP Cartridges must have been removed from 4°C storage by the Study Pharmacist and allowed to equilibrate to room temperature for at least one hour prior to opening the primary pouch, and used within 3 hours of removing from 4°C storage. Once the primary pouch has been opened the MAP must be applied within 15 minutes (+5).

MIMIX MAPs will be applied using the separate spring-powered applicator described in Section 1.2 Vaccine Design following the MIIMX MAP System use sequence shown in Figure 5 and as outlined in the Instructions for Use document Briefly, the MAP Cartridge is loaded onto the applicator by pushing the applicator directly into the open tray and between the snap hooks of the MAP Cartridge. The snap hooks are used to attach the MAP Cartridge to the applicator and to hold the Cartridge in place for MAP delivery. The loaded applicator must be held perpendicular to and just touching the skin of the application site with the arm supported.

The top of the applicator is pressed downward to apply the MAP. Following MAP deployment, the MAP Applicator and Cartridge are immediately removed from the skin, returned to the original Applicator shipping pouch, then to the original Applicator box provided by Vaxess and retained. The patch will remain on the skin for a minimum of 5 minutes and no longer than 10 minutes. The MAP is removed by study staff using gloved fingers, by gently tensioning the skin around the patch with one hand, whilst pulling the MAP directly up and away from the skin with the other hand. Following removal, the MAP should be discarded.

After the MAP has been removed, skin tolerability assessments (Appendix 1: Local Tolerability Scoring) including pain, induration, tenderness and erythema will be assessed 1 (±2), 30 (±5) and 60 (±5) minutes post-MAP removal. Other local tolerability assessments (Appendix 2: Other Local Tolerability Scoring) including bruising, itching, skin-flaking and pigmented coloration will be assessed 60 (±5) minutes post-MAP removal.

If erythema, bruising or induration ≥1 cm around the treatment site is present, the maximum diameter will be recorded.

The MAP application site will be photographed with the study specific iPhone 1 (±2), 30 (±5) and 60 (±5) minutes post-MAP removal and with the iPhone attached to the study specific DermLite Imaging system 60 (±5) minutes post-MAP removal. Please refer to the Imaging Instructions document for additional details.

During the follow up visits, tolerability assessments, photographs of the MAP application site and blood samples will be taken for safety and immunogenicity assessments per the Assessment Schedule Table 5.

Figure 5: MIMIX MAP System Use Steps

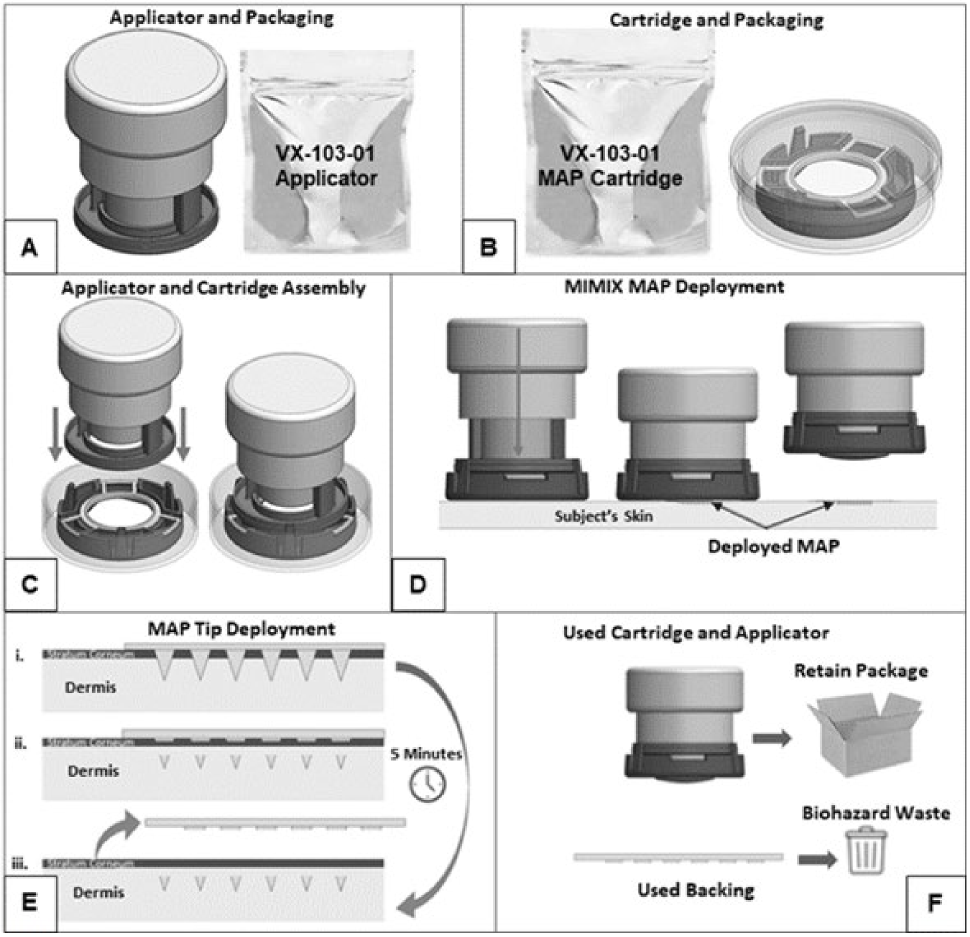

6.1.2 Additional Study Treatments

No other treatment beyond investigational drug and control are included in this trial.

6.1.3 Treatment Arms/Groups

Participants will be randomized at Day 1 to one of the following "n" treatment arms/groups. The participants will be randomized in a 1:1:1 manner.

Approximately 45 healthy adult participants will be enrolled in the study.

The planned doses are the following:

- Group 1: 15 µg
- Group 2: 7.5 µg
- Group 3: Placebo

6.2 Other Treatments

6.2.1 Concomitant Therapy

Concomitant therapy for intercurrent medical events should be discussed between the sponsor and the investigator or his designee.

Approved killed, inactivated, peptide, deoxyribonucleic acid and ribonucleic acid (RNA) vaccines may be permitted 30 days prior to VX-103 dosing or 60 days after VX-103 dosing according to the investigator's discretion and per local guidance. All medications, procedures, and significant non-drug therapies (including physical therapy) administered after the participant was enrolled into the study must be recorded on the appropriate electronic case report form (eCRF).

Each concomitant drug must be individually assessed against all exclusion criteria/prohibited medication. If in doubt, the investigator should contact the Sponsor’s medical monitor or designee before randomizing a participant or allowing a new medication to be started. If the participant is already enrolled, contact Vaxess or designee to determine if the participant should continue participation in the study.

6.2.2 Restriction for study participants

For the duration of the study, participants should be informed and reminded of the restrictions outlined in this section.

6.2.2.1 Restrictions to prevent pregnancy

Non-clinical reproductive toxicity studies have not yet been performed to date with VX-103. It is unknown if there is any potential impact on a pregnancy by VX-103. Therefore, sexually active males and females participating in this clinical trial are required to use highly effective contraception methods.

The following highly effective methods of contraception are accepted:

- Established use of oral, intravaginal, or transdermal combined (estrogen and progestogen containing) hormonal contraception associated with inhibition of ovulation.
- Established use of oral, injectable, or implantable progestogen-only hormonal contraception associated with inhibition of ovulation.
- Placement of an intrauterine device (IUD) or intrauterine hormone-releasing system (IUS).
- Barrier methods of contraception: male condom with either cap, diaphragm or sponge with spermicide (double barrier methods). The use of double barrier methods should always be supplemented with the use of a spermicide. Female condom and male condom should not be used together.
- Male sterilization (provided that the partner is the sole sexual partner of the patient and that the sterilized partner has received medical assessment of the surgical success.)

- Sexual abstinence

6.2.2.2 Dietary restrictions

1. No alcohol for 48 hours prior to enrollment and for 24 hours after Day 1.
2. The use of recreational Marijuana is only prohibited during the 7 days prior to enrollment until after Study Day 8.

6.3 Participant numbering, treatment assignment, randomization

6.3.1 Participant numbering

Each participant is identified in the study by a Participant Number (Participant No.) that is assigned when the participant is enrolled for screening and is updated for the participant upon enrollment in the trial. The Participant No. consists of the Site Number (Site No.) (as assigned by Vaxess or designee to the investigative site) with a sequential participant number suffixed to it, so that each participant's participation is numbered uniquely across the entire database. Upon signing the informed consent form, the participant is assigned to the next sequential Participant No. available.

6.3.2 Treatment assignment, randomization

A randomization list will be produced by Icon by following their internal process using a validated system that automates the random assignment of treatment arms to randomization numbers in the specified ratio.

The randomization scheme for participants will be reviewed by the study biostatistician.

6.4 Treatment blinding

All groups are unblinded except the rater and subject who will remain blinded to study treatment throughout the study, except where indicated below.

The identity of the treatments will be concealed by the use of study vaccines that are all identical in packaging, labeling, schedule of administration, appearance, and odor.

Site staff

All site staff that have been delegated as a rater on the Delegation of Authority log will be blinded to study treatment during treatment allocation and participant dosing.

Unblinding a single participant at site for safety reasons (necessary for participant management) will occur via an emergency system in place at the site.

The unblinded pharmacy staff will receive a randomization list or treatment allocation information from Vaxess or designee with the appropriate treatment allocation numbers. Appropriate measures must be taken by the unblinded pharmacist to ensure that the treatment assignments are concealed from the blinded site staff.

Sponsor staff

The following unblinded sponsor roles are required for this study:

- Unblinded clinical staff managing drug re-supply to site
- Blinded sample analyst(s) (immunogenicity)

The monitors are not provided with a randomization list directly but will be unblinded through review of source documentation compiled by the unblinded pharmacist or designee, which details treatment allocation to individual participants. The monitors will also be able to review the treatment allocation information/randomization list provided to the unblinded pharmacist. The name(s) of the monitor(s) are detailed in the monitoring plan, if applicable.

Sponsor or designee clinical staff are required to assist in the management and re-supply of investigational drug product. These individuals are not provided with randomization lists directly but may be unblinded through communication of drug re-supply needs via the unblinded site pharmacists.

The sample analysts will receive a copy of the randomization schedule to facilitate analysis of the samples. The sample analysts will provide the sample data to the study team under blinded conditions unless otherwise allowed.

The study statistician will be able to access the full randomization list from the start of the study and is allowed to share unblinded information with the rest of the clinical trial team as appropriate for internal decision purposes, as outlined in Table 4. For example, unblinded summaries and unblinded individual data can be shared with the team whenever necessary.

Study programmers and other personnel involved in study data analysis (e.g. immunogenicity expert) are allowed to access treatment assignment information from the start of the study for the purpose of data analysis.

The Vaxess clinical trial team is allowed to share unblinded results with other sponsor staff (e.g. decision boards) as required for internal decision making on the study or the project while the study is ongoing.

All unblinded personnel will otherwise keep randomization lists and data or information that could unblind other study team members confidential and secure except as described above.

Following final database lock, all roles may be considered unblinded.

Table 4: Blinding and Unblinding Plan

Role	Randomization list generated	Treatment allocation & dosing	Safety event (single participant unblinded)
Participants	B	B	UI
Rater Only staff	B	B	UI
Unblinded site staff e.g. pharmacy staff and data entry	B	UI	UI
Vaccine Clinical Supply and Randomization	UI	UI	UI
Unblinded sponsor staff or designee e.g. for study treatment resupply, unblinded monitor(s), sample analyst(s)	B	UI	UI
Unblinded Pharmacovigilance sponsor staff or designee	B	UI	UI
Statistician/statistical programmer/ data analysts (e.g. immunogenicity)	B	UI	UI
All other sponsor staff not identified above (i.e. trial team, project team, management & decision boards, support functions)	B	UI	UI

Key:
UI: Allowed to be unblinded on individual participant level
B: Remains blinded
NA: Not applicable to this study

6.5 Dose escalation and dose modification

Investigational or other study treatment dose adjustments and/or interruptions are not permitted.

6.6 Additional treatment guidance

6.6.1 Treatment compliance

VX-103 will be administered at the study site. The site must maintain an accurate record of the receipt and of dispensing of study treatment in a drug accountability log. Note: study treatment includes investigational product and placebo.

Immunogenicity parameters will be determined in all participants treated with VX-103, as detailed in Section 8.5.1 Immunogenicity.

6.6.2 Recommended treatment of adverse events

At present there is insufficient information to provide specific recommendations regarding treatment of AEs.

In the case of unanticipated AEs, the investigator should consider the criteria for study interruptions (see Section 9.1 Discontinuation and completion).

Medication, including the use of acetaminophen that is used to treat AEs must be recorded on the appropriate eCRF.

6.6.3 Emergency breaking of assigned treatment codes

Emergency breaking of the assigned treatment code must only be undertaken when it is essential to treat the participant safely and efficaciously.

Most often, study treatment discontinuation and knowledge of the possible treatment assignments are sufficient to treat a study participant who presents with an emergency condition. Since only the rater and subject are blinded, the site will maintain a copy of the randomization and treatment list in a secure location. Should a blind need to be broken, the site staff will follow their local procedures and will notify the Sponsor immediately.
The investigator must also immediately inform the sponsor's local monitor that the code has been broken.

It is the investigator’s responsibility to ensure that there is a dependable procedure in place to allow access to break the blind at any time in case of emergency. The investigator will provide:

- protocol number
- participant number

In addition, oral and written information to the participant must be provided on how to contact the investigator’s backup in cases of emergency, or when he/she is unavailable, to ensure that un-blinding can be performed at any time.

6.7 Preparation and dispensation

Each study site will be supplied with investigational product in packaging as described under investigational and control drugs section (as described in Section 6.1.1 Investigational and control product).

Clinical supplies are to be dispensed only in accordance with the protocol. For preparation of study treatment, the unblinded pharmacist or authorized designee will access the treatment allocation from the provided randomization list. Study vaccine delivered by MIMIX MAP (VX-103 or placebo) will be prepared by an unblinded local pharmacist or designee at the study site.

Please refer to the pharmacy manual for detailed instructions on drug preparation and administration. Appropriate documentation of the participant-specific dispensing process must be maintained.

7 INFORMED CONSENT PROCEDURES

Eligible participants may only be included in the study after providing (witnessed, where required by law or regulation), institutional review board/ independent ethics committee (IRB/IEC)-approved informed consent.

If applicable, in cases where the participant's representative(s) gives consent (if allowed according to local requirements), the participant must be informed about the study to the extent possible given his/her understanding. If the participant is capable of doing so, he/she must indicate agreement by personally signing and dating the written informed consent document.

Informed consent must be obtained before conducting any study-specific procedures (e.g. all of the procedures described in the protocol). The process of obtaining informed consent must be documented in the participant source documents.

Vaxess or designee will provide to investigators in a separate document a proposed informed consent form that complies with the ICH GCP guidelines and regulatory requirements and is considered appropriate for this study. Any changes to the proposed consent form suggested by the investigator must be agreed by Vaxess or designate before submission to the IRB/IEC.

Information about common side effects already known about the investigational product can be found in the IB. This information will be included in the participant informed consent and should be discussed with the participant during the study as needed. Any new information regarding the safety profile of the investigational product that is identified between IB updates will be communicated as appropriate, for example, via an investigator notification or an aggregate safety finding. New information might require an update to the informed consent and then must be discussed with the participant.

The following informed consents are included in this study:

- Main study consent, which also includes:
  - A subsection that requires a separate signature for the 'Optional Consent for Additional Research' to allow future research on data/samples collected during this study
- As applicable, Pregnancy Outcomes Reporting Consent for female participants or the female partners of any male participants who took study treatment

Male participants must be informed that if a female partner becomes pregnant while he is enrolled in the study, contact with the female partner will be attempted to request her consent to collect pregnancy outcome information.

A copy of the approved version of all consent forms must be provided to Vaxess or designate after IRB/IEC approval.

8 VISIT SCHEDULE AND ASSESSMENTS

The Assessment Schedule (Table 5) lists all assessments and when they are performed. All data obtained from these assessments must be supported in the participant's source documentation.

Participants should be seen for all visits/assessments as outlined in the assessment schedule (Table 5) or as close to the designated day/time as possible. Missed or rescheduled visits should not lead to automatic discontinuation. Participants who prematurely discontinue the study for any reason should be scheduled for a visit as soon as possible, at which time all of the assessments listed for the final visit will be performed. At this final visit the adverse event and concomitant medications will be recorded on the eCRF.

The preferred sequence of data collection during study visits is vital signs and followed by blood sampling.

Every effort will be made to take the immunogenicity sample at the protocol specified time. Other assessments e.g. vital signs, etc. will be taken prior to the immunogenicity sample.

Table 5: Assessment Schedule

Procedures	Screening Visit 00, Day±30 to 0	Enrollment/Baseline Visit 01, Day 1				Visit 02, Day 2 1 day post Dose (+1)	Visit 03, Day 4 3 days post Dose (+1)	Telephone Visit Day 5, 6, 7	Visit 04 Day 8 (± 2)	Visit 05 Day 15 (± 2)	Visit 06 Day 29 (± 2)	Visit 7 Day 57 (± 2)	Visit 8 Day 85 (± 7)	Visit 9 Day 119 (± 7)	Final Study Visit 10 180 (± 7)	Unscheduled Visit	Early Termination Visit
		Pre Dose	1 min Post Dose	30 min Post Dose	60 min Post Dose												
**Informed Consent[a]**	X																
**Demographic Data[b]**	X																
**Review Eligibility Criteria**	X	X															
**Medical History[c]**	X																
**Physical Exam[d]**	X	X				X	X		X	X	X	X	X	X	X	X	X
**Height & Weight**	X																
**Concomitant Medications[e]**	X	X				X	X		X	X	X	X	X	X	X	X	X
**Vital Signs[g]**	X	X				X	X		X	X	X	X	X	X	X	X	X
**Hematology[h]**	X	X					X				X				X	X	X
**Chemistry[i]**	X	X					X				X				X	X	X
**HBV, HPC, HIV Serology testing**	X																
**COVID-19 Test**	X	X															
**Urinalysis**	X																
**Pregnancy Test[j]**	X	X															
**Urine Drug & Alcohol Screen[k]**	X	X															
**VX-103 Randomization & Study Dosing**		X															

**ILI Surveillance**	X	X															
**Photographs of Treatment Site**[l]		X	X	X	X	X	X		X	X	X	X			X		
**Solicited Local AEs Appendix 1**		X	X	X	X	X	X	X	X	X*	X*	X*			X*		
**Solicited Local AEs Appendix 2**		X			X	X	X	X	X	X**	X**	X**			X**		
**Solicited Systemic AEs Appendix 3**		X			X	X	X	X	X								
**Adverse Event Collection (Unsolicited)**[m]	X	Days 1-29															
**SAEs, MAEs and NOCMCs**		Days 1-180															
**Serum for Serological Immunogenicity Assays: HAI Titers**		X									X	X	X	X	X		X
**Serum for Exploratory Endpoints: HAI Drifted Strain; Anti HA IgG, Anti- Fibroin IgG, IgE and MN Titers**		X									X				X		X
**Safety Telephone Contact**[n]								X									

*Erythema assessments only from Appendix 1: Local Tolerability Scoring
**Coloration assessment only from Appendix 2: Other Local Tolerability Scoring

a. The signed informed consent of the subject must be obtained before study participation.
b. Record demographic data such as year of birth, sex, race (ethnicity and geographic ancestry) in the subject's eCRF
c. Obtain the subject's medical history by interview and/or review of the subject's medical records and record any pre-existing conditions or signs and/or symptoms present in a subject prior to the first study vaccination in the eCRF.
d. Perform a physical examination of the subject, including assessment of oral body temperature and resting vital signs: systolic/diastolic blood pressure, heart rate and respiratory rate after at least 5 minutes of rest, pulmonary auscultation. Collected information needs to be recorded in the eCRF. Any findings from the(se) physical examination(s) need to be recorded in the subject's medical record, in the medical history screen of the eCRF or reported as an AE or SAE, as applicable, if they meet the protocol definition of AE or SAE. Treatment of any abnormality observed during physical examination has to be performed according to local medical practice outside this study or by referral to an appropriate specialist. After the screening visit, an abbreviated physical exam should be performed at each visit until the final study visit.

e. All participants will be questioned about concomitant medications at each visit. Medications taken within 30 days prior to day 1 will be documented in the eCRF. Any changes to a subject's medications will also be documented in the eCRF.
f. The time of placement and removal of the MIMX-MAP will be recorded in the eCRF. The unblinded site staff shall refer to the pharmacy manual for specific instructions. VX-103-01 MAP should be placed on the skin for a minimum of 5 minutes and a maximum of 10 minutes
g. Vital signs measurements include temperature, respiration rate, heart rate, and blood pressure after a 5-minute seated rest.
h. Hematology: will be drawn and analyzed locally. The data will be captured in the eCRF and the lab reports stored with the subject's source documents at the site.
i. Chemistry: will be drawn and analyzed locally. The data will be captured in the eCRF and the lab reports stored with the subject's source documents at the site.
j. Female participants of childbearing potential are to have a urine pregnancy test prior to any study vaccine administration. The test result is to be recorded in the eCRF. The investigational product/products may only be administered if the pregnancy test is negative. A serum pregnancy test instead of a urine pregnancy test should only be considered if required by country, local or ethics committee regulations. If a serum pregnancy test instead of a urine pregnancy test is required by country, local or ethics committee regulations, a blood sample will be collected from women of childbearing potential at the vaccination visits and will be used for the test as per local guidance. Note: Pregnancy test must be performed even if the subject is menstruating at the time of the study visit.
k. At minimum, a basic urine alcohol & drug screen will be used to analyze for amphetamines, Opiates, Phencyclidine (PCP), Cocaine, Marijuana, and alcohol.
l. All photographs should be taken with the study specific iPhone or the study specific DermLite DL4 Dermatoscope attached to the iPhone. Subject ID, Subject initials, and timepoint should be included in each iPhone photograph with a label. On Study Day 1, iPhone photographs should be taken of the treatment site both pre dosing (within 30 minutes prior to dosing) and 1 + 2 minutes, 30 ± 5 and 60 ± 5 minutes post removal of the MAP. iPhone photographs will also be taken a single time on Days 2, 4, 8, 15, 29, 57 and 180. For each iPhone photograph hold the phone 8-10 cm above the Application site, take one, in focus, 1x magnification photo. Capture the Application site and label.
Dermatoscope images will be taken with the Dermatscope attached to the iPhone, the lens spacer rotated to '0' and the faceplate removed. The image will be taken with the lens spacer gently touching the application site periphery. The rim of the Dermatoscope lens spacer should be wiped with alcohol before and after use. Photographs with the Dermatoscope attached to the iPhone should be taken of the treatment site pre dosing (within 30 minutes prior to dosing) and a single time on Days 29 and 180. Photographs with the Dermatoscope should be 'captioned' using the iPhone 'add a caption' function. The Caption should include the Subject ID, initials and timepoint. All photos should be taken under consistent lighting conditions. All photographs will be uploaded to a secure file system, BOX. Please see VX-103-01 Imaging Instructions for additional instructions.
m. Standard adverse event collection will take place from the time the informed consent is signed through Day 29. This will capture all adverse events that are not included from the solicited adverse event collection appendices.
n. These calls are to query for any adverse events, complete Appendix 1, 2 and the systemic section of Appendix 3 and determine if an on-site visit is needed.

8.1 Screening

Each subject will be screened prior to enrollment on VAX-103-01. Re-screens will not be permitted.

8.1.1 Eligibility screening

Results of below screening measurements will be available as source data at the study site and will not be recorded within the eCRF.

8.1.1.1 Hepatitis screen, HIV screen

All participants will be screened for HBsAg and, if standard local practice, Hepatitis B core antigen (HBcAg). Screening for Hepatitis C will be based on Hepatitis C virus (HCV) antibodies and if positive, HCV RNA levels should be determined.

Evaluation for HIV seropositivity will be performed, and, if positive, confirmation by a second technique available at the laboratory site e.g. Western blot. Appropriate counseling will be made available by the investigator in the event of a positive confirmatory test. Notification of state and federal authorities, as required by law, will be the responsibility of the investigator.

8.1.1.2 Alcohol test, Drug screen

Participants will be tested for substances of abuse (e.g. alcohol, amphetamines, barbiturates, benzodiazepines, cannabinoids, cocaine and opiates). A positive screen for marijuana is not considered to be a screen-failure unless it is within 7 days of enrollment or the investigator believes there to be a history of abuse.

8.1.2 Information to be collected on screening failures

Participants who sign an informed consent form and subsequently found to be ineligible prior to randomization will be considered a screen failure. The reason for screen failure should be recorded on the appropriate eCRF. The demographic information, informed consent, and inclusion/exclusion pages must also be completed for screen failure participants. No other data will be entered into the clinical database for participants who are screen failures, unless the participant experienced a SAE during the screening phase (see Section 10 SAFETY MONITORING AND REPORTING for SAE reporting details). If the participant fails to be randomized, the study team should document the reason within the appropriate eCRF.

Participants who are randomized and fail to start treatment, e.g. participants randomized in error, will be considered an early terminator. The reason for early termination should be recorded on the appropriate eCRF.

8.2 Participant demographics/other baseline characteristics

Participant demographics: date or year of birth (if permitted), sex, race, predominant ethnicity (if permitted) and relevant medical history/current medical conditions (until date of signature of informed consent) will be recorded in the eCRF. Where possible, the diagnosis and not symptoms should be recorded.

All prescription medications, over-the-counter drugs and significant non-drug therapies prior to the start of the study must be documented. See the protocol Section 6.2.1 Concomitant Therapy for further details on what information must be recorded on the appropriate page of the eCRF.

8.3 Efficacy

No efficacy assessments are planned for this study, and immunogenicity samples will be collected at the time-points defined in the assessment schedule (Table 5).

Immunogenicity samples will be obtained and evaluated in all participants at all dose levels, including the placebo group. In order to better define the immunogenicity profile, the timing of the sample collection may be altered based on emergent data.

Immunogenicity assessments are described in Section 8.5.1 Immunogenicity.

8.3.1 Appropriateness of efficacy assessments

Not applicable.

8.4 Safety, Reactogenicity and Tolerability

Safety assessments are specified below (Table 6) with the assessment schedule detailing when each assessment is to be performed. Each clinical research site must have epinephrine on hand and the staff should administer in case of anaphylaxis. Should anaphylaxis occur, the site staff should treat according to institutional guidelines.

For details on AE collection and reporting, refer to Section 10 SAFETY MONITORING AND REPORTING.

Table 6: Assessment Specification

Assessment	Specification
Physical examination	A complete physical examination will include the examination of general appearance, skin, neck (including thyroid), eyes, ears, nose, throat, lungs, heart, abdomen, back, lymph nodes, extremities, vascular, and neurological. If indicated based on medical history and/or symptoms, rectal, external genitalia, breast, and pelvic exams will be performed. A complete physical examination will be at screening and final study or early termination visits.<br>An abbreviated physical exam will include the examination of general appearance, axillary and cervical lymph nodes, site of MAP, any other body system directed by participant's symptoms, and vital signs (blood pressure [SBP and DBP] and pulse). An abbreviated physical exam will be at visits starting from post screening and before final study visit except where a complete physical examination is required (see above).<br>Information for all physical examinations must be included in the source documentation at the study site. Clinically relevant findings that are present prior to signing informed consent must be recorded on the appropriate eCRF that captures medical history. Significant findings made after signing informed consent which meet the definition of an AE must be recorded as an adverse event.
Vital signs	Vital signs will include the collection of oral temperature (recorded in °C), blood pressure (BP) and pulse measurements. The same method for obtaining temperature should be used for the duration of the trial.<br>After the participant has been sitting for 5 minutes, with back supported and both feet placed on the floor, systolic and diastolic BP will be measured using an automated validated device, e.g. OMRON with an appropriately sized arm cuff. In case the arm cuff sizes

	available are not large enough for the participant's arm circumference, a sphygmomanometer with an appropriately sized cuff may be used.<br>If vital signs are out-of-range at screening and/or baseline (see Exclusion Criteria Section 5 STUDY POPULATION of the protocol for details), two additional readings can be obtained, so that a total of three consecutive assessments are made, with the participant seated quietly for approximately five minutes preceding each repeat assessment. The last reading must be within the ranges provided in the eligibility criteria in order for the participant to qualify.<br>In case of repeated vital assessments, the eCRF should contain the qualifying results.
Safety Laboratory Assessments	Hematology, chemistry, coagulation parameters and urinalysis are obtained from the site's local laboratory (See Table 5).
Height and weight	Height is obtained in centimeters (cm) and body weight is obtained in kilograms (kg) and rounded to the nearest 0.1 kg. Weight is obtained in indoor clothing, without shoes.<br>Body mass index (BMI) will be calculated using the following formula:<br>BMI = Body weight (kg) / [Height (m)]^2<br>Indicate whether rounding should be done to nearest whole number or not (e.g. if range in inclusion/exclusion criteria is 18-30 kg/m^2 and calculated BMI is 30.44, discuss with Medical Lead whether the BMI value can be rounded to 30 kg/m^2).<br>The Screening Visit height measurement will be used for BMI calculations throughout the study.
Photographs of treatment site	Site staff will take a picture of the treatment site and upload to a secure file server at each scheduled interval.
Local Reactogenicity and Tolerability and Systemic Safety Assessments	Local reactogenicity and tolerability, specifically, pain, induration, tenderness, erythema and edema will be assessed according to Appendix 1: Local Tolerability Scoring.<br>Other local tolerability measures including bruising, itching, skin flaking and application site pigmented skin coloration will be assessed as outlined in Appendix 2: Other Local Tolerability Scoring.<br>Systemic safety measures will be assessed as outlined in Appendix 3: Systemic Toxicity Grading Scales.<br>All assessments will take place according to Table 5.<br>If an AE is reported by telephone for which an in-clinic assessment may be warranted, an unscheduled (optional) visit can be performed at any time.
Medical Questioning	The investigator will assess the participant for any reported AEs and ensure approval is provided before discharging the participant from the site.

8.4.1 Laboratory evaluations

Laboratory samples will be collected at time points as indicated in Table 5 Schedule of Assessments.

Local laboratory will be used for analysis of scheduled safety labs.

A midstream urine sample (approx. 30 mL) will be obtained, in order to avoid contamination with epithelial cells and sediments and allow proper assessments.

Clinically significant abnormalities must be recorded as either medical history/current medical conditions or adverse events as appropriate.

In the case where a laboratory range is not specified by the protocol, but a value is outside the reference range for the laboratory at screening and/or baseline, a decision regarding whether the

result is of clinical significance or not shall be made by the investigator (in consultation with the sponsor or designate) and shall be based, in part, upon the nature and degree of the observed abnormality. The assessment may be repeated once prior to randomization.

In all cases, the investigator must document in the source documents, the clinical considerations (i.e., result was/was not clinically significant and/or medically relevant) in allowing or disallowing the participant to continue in the study.

All abnormal lab results must be evaluated for criteria defining an adverse event and reported as such if the criteria are met. For those lab adverse events, repeated evaluations are mandatory until normalization of the result(s) or until the result is no longer considered to be clinically significant.

Table 7: Clinical laboratory parameters collection plan

Test Category	Test Name
Hematology	Hematocrit, Hemoglobin, MCH, MCHC, MCV, Platelets, Red blood cells (RBC), White blood cells (WBC), RBC Morphology, Differential (Basophils, Eosinophils, Lymphocytes, Monocytes, Neutrophils, Bands, Other (absolute value preferred, %'s are acceptable)
Chemistry	Albumin, Alkaline phosphatase by Fractionated Alkaline Phosphatase , ALT, AST, Gamma-glutamyl-transferase (GGT), Lactate dehydrogenase (LDH), Bicarbonate, Calcium, Magnesium, Phosphorus, Chloride, Sodium, Potassium, Cr, Creatine kinase, Direct Bilirubin, Indirect Bilirubin (only if total bilirubin is abnormal), Total Bilirubin, Total Cholesterol, LDL, HDL, Total Protein, Triglycerides, Blood Urea Nitrogen (BUN) or Urea, Uric Acid, Amylase, Lipase, Glucose (choose as applicable: non-fasting)
Urinalysis	Macroscopic Panel (Dipstick) (Color, Bilirubin, Blood, Glucose, Ketones, Leukocytes esterase, Nitrite, pH, Protein, Specific Gravity, Urobilinogen)<br>If the dipstick is abnormal, then Microscopic Panel (RBCs, WBCs, Casts, Crystals, Bacteria, Epithelial cells)
Coagulation	Prothrombin time (PT), International normalized ratio [INR]), Partial thromboplastin time (PTT), Activated partial thromboplastin time (APTT)
Thyroid	T3 [free], T4 [free], TSH
Hepatitis markers	HBsAg, HBcAb, HCV (HCV - RNA)
Additional tests	SARS-CoV-2 seropositivity or SARS-COV2 PCR or Rapid Test per local site policy
Pregnancy test and assessments of fertility	Luteinizing hormone (LH) and follicle stimulating hormone (FSH) (female only as outlined in Section 8.4.3 Pregnancy and assessments of fertility)

8.4.3 Pregnancy and assessments of fertility

Women of childbearing potential are excluded from this study.

Assessments of fertility

Medical documentation of oophorectomy, hysterectomy, or tubal ligation must be retained as source documents. Subsequent hormone level assessment to confirm the woman is not of childbearing potential must also be available as source documentation in the following cases:

- Surgical bilateral oophorectomy without a hysterectomy
- Reported 12 months of natural (spontaneous) amenorrhea with an appropriate clinical profile.

In the absence of the above medical documentation, LH and FSH testing is required of any female participant regardless of reported reproductive/menopausal status at screening.

8.4.4 Appropriateness of safety measurements

The porcine model has been extensively used as a model for studying human skin responses to medical devices. Compared to other common animal models, the epidermis and dermis of the pig has a thickness that is most similar to humans. The pig epidermis ranges from 30 to 140 µm; human skin ranges from 50 to 120 µm. In addition, the skin of the pig is more firmly attached to the underlying structures as seen in humans (Abdullahi et al., 2014). Further, pig skin heals through re-epithelialization, unlike mice, rats, and other rodents whose skin heals through contraction. These features make the minipig a particularly useful model for evaluating the local and systemic effects of MIMIX MAPs as well as a useful model for determining if the MIMIX tip material will ultimately be absorbed or ejected and the likely timeline for this event in humans. Vaxess has carried out a pilot evaluation of MAP tolerability and tip persistence and a pilot repeat-dose systemic toxicology study in the Göttingen minipig model. We find that the minipig develops robust immune responses to the VX-103 vaccine antigen. In both pilot studies the results showed transient erythema and edema at the MAP application site that resolved by 28 days post MAP application. The erythema and edema reported, characteristically resolves in the first several days, then reappears and resolves by 28 days. No detectable adverse safety findings were reported. The pilot implantation effects study provides evidence that, by day 90, tip material for MIMIX FLU test articles is absorbed or ejected, there is no longer inflammation, and tissue has been restored to normal structure.

In sum, no adverse systemic events have been reported to date in our preclinical safety studies and local reactogenicity has been limited to transient erythema and edema. These data have been used to inform the design of the Phase 1 clinical trial.

8.5 Additional assessments

8.5.1 Immunogenicity

To describe the immunogenicity of VX-103, serum samples for determination of HAI antibodies will be collected at the time points defined in the Assessment Schedule (Table 5).

HAI titers, virus microneutralization titers, anti-HA, anti-fibroin IgG and total IgE will be assessed in serum of all participants, including the placebo group.

Instructions are outlined in the lab manual regarding tube handling, sample collection, numbering, processing, and shipment. In order to better define immunogenicity, the timing of the serum sample collection may be altered based on emergent data.

Serum samples remaining after completion of analysis may be used for exploratory assessment or other bioanalytical purpose (e.g., other bioanalytical exploratory assessments).

Immunogenicity parameters

The immunogenicity of the study vaccine will be evaluated primarily by measuring the number of participants who demonstrate seroconversion by developing a four-fold rise in the HAI titer following vaccination HAI titers. Mean fold rises in geometric mean HAI titers will be compared

across treatment groups at each timepoint. Immunogenicity of the vaccine will also be evaluated by measuring an increase in serum microneutralization titers and anti-HA IgG titers postvaccination. Standard statistical tests will be used to define a significant rise in antibody titers.

8.5.2 Use of residual biological samples

There will be no residual samples from this trial.

9 STUDY DISCONTINUATION AND COMPLETION

9.1 Discontinuation and completion

9.1.1 Study treatment discontinuation and study discontinuation

The study is made of single dose administration. Therefore, discontinuation of study treatment is not applicable.

However, during the vaccination, the MAP should be removed if any of the following occur:

- Severe (symptomatic bronchospasm with or without urticaria, allergy related edema / angioedema, hypotension) or reaction
- Moderate (Rash, flushing, urticaria, dyspnea, drug fever > 38°C) or worse hypersensitivity reaction
- Severe other adverse event
- Local reaction
- General reaction such as an anaphylactic-like reaction
- General bad tolerance

Participants who do not wish to participate in the study further should NOT be considered withdrawn from the study UNLESS they withdraw their consent (see Section 9.1.2 Withdrawal of informed consent [WOC]). Where possible, they should return for the assessments indicated in the Assessment Schedule (Table 5). If they fail to return for these assessments for unknown reasons, every effort (e.g. telephone, e-mail, letter) should be made to contact the participant/pre-designated contact as specified in the lost to follow-up section. This contact should preferably be done according to the study visit schedule.

If the participant cannot or is unwilling to attend any visit(s), the site staff should maintain regular telephone contact with the participant. This telephone contact should preferably be done according to the study visit schedule.

After treatment discontinuation, at a minimum, in abbreviated visits, the following data should be collected at clinic visits or via telephone/email contact:

- New / concomitant treatments
- AEs / SAEs

If discontinuation occurs because treatment code has been broken, please refer to Emergency breaking of treatment code Section 6.6.3 Emergency breaking of assigned treatment codes.

9.1.1.1 Replacement policy

If a participant is not enrolled, a back-up subject will be enrolled until approximately 45 participants have been dosed.

9.1.2 Withdrawal of informed consent

Participants may voluntarily withdraw consent to participate in the study for any reason at any time. WOC occurs only when a participant:

- Does not want to participate in the study anymore,

and

- Does not want any further visits or assessments

and

- Does not want any further study related contacts

In this situation, the investigator should make a reasonable effort (e.g. telephone, e-mail, letter) to understand the primary reason for the participant's decision to withdraw his/her consent and record this information.

Where consent to the use of personal and coded data is not required, participant therefore cannot withdraw consent. They still retain the right to object to the further use of personal data.

No further assessments will be conducted, and the data that would have been collected at subsequent visits will be considered missing.

Further attempts to contact the participant are not allowed unless safety findings require communicating or follow-up.

All efforts should be made to complete the assessments prior to study discontinuation. A final evaluation at the time of the participant's study discontinuation should be made as detailed in the assessment schedule (Table 5).

Vaxess or designate will continue to retain and use all research results (data) that have already been collected for the study evaluation.

9.1.3 Lost to follow-up

For participants whose status is unclear because they fail to appear for study visits without stating an intention to discontinue or withdraw, the investigator must show "due diligence" by documenting in the source documents steps taken to contact the participant, e.g. dates of telephone calls, registered letters, etc. A participant should not be considered as lost to follow-up until due diligence has been completed.

9.1.4 Study stopping rules

Event	Number of Participants
Death or any life-threatening SAE	1
Any SAE that cannot reasonably be attributed to a cause other than vaccination	1
Any withdrawal from the study or withdrawal from study	1

vaccine(s)/product(s) (by investigator or subject request) following a grade 3 AE that cannot reasonably be attributed to a cause other than vaccination	
Any local or general solicited AE leading to hospitalization, or fever > 40°C or 104°F (oral route) that cannot reasonably be attributed to a cause other than vaccination, or necrosis at the injection site, within the 7-day (Days 1-7) post-vaccination period	1
Any grade 3 solicited local AE lasting 48h or more in an investigational group, within the 7-day (Days 1-7) post-vaccination period	3
Any grade 3 solicited general AE lasting 48h or more in an investigational group, that cannot reasonably be attributed to a cause other than vaccination, within the 7- day (Days 1-7) post-vaccination period	1
Any grade 3 unsolicited AE in an investigational group, that cannot reasonably be attributed to a cause other than vaccination, within the 7-day (Days 1-7) postvaccination period OR Any grade 3 abnormality in the same pre-specified hematological or biochemical laboratory parameters* in an investigational group, that cannot reasonably be attributed to a cause other than vaccination, within the 7 days post-vaccination	1

9.1.5 Early study termination by the sponsor

The study can be terminated by Vaxess at any time.

Reasons for early termination

- Unexpected, significant, or unacceptable safety risk to participants enrolled in the study
- Decision based on recommendations from applicable board(s) after review of safety and immunogenicity data
- Discontinuation of study drug development

In taking the decision to terminate, Vaxess will always consider participant welfare and safety. Should early termination be necessary, participants must be seen as soon as possible and treated as a prematurely withdrawn participant. The investigator may be informed of additional procedures to be followed in order to ensure that adequate consideration is given to the protection of the participant's interests. The investigator or sponsor depending on local regulation will be responsible for informing IRBs/IECs of the early termination of the trial.

9.2 Study completion and post-study treatment

Study completion is defined as when the last participant finishes their study completion visit and any repeat assessments associated with this visit have been documented and followed up appropriately by the investigator or, in the event of an early study termination decision, the date of that decision. Each participant will be required to complete the study in its entirety and thereafter no further study treatment will be made available to them.

The information collected is kept as source documentation. All SAEs reported during this time period must be reported as described in Section 10.1.3 SAE reporting. Documentation of attempts to contact the participant should be recorded in the source documentation.

10 SAFETY MONITORING AND REPORTING

10.1 Definition of adverse events and reporting requirements

10.1.1 Adverse events

An AE) is any untoward medical occurrence (e.g. any unfavorable and unintended sign [including abnormal laboratory findings], symptom or disease) in a clinical investigation participant after providing written informed consent for participation in the study. Therefore, an AE may or may not be temporally or causally associated with the use of a medicinal (investigational) product.

The investigator has the responsibility for managing the safety of individual participant and identifying AEs.

Vaxess or designate qualified medical personnel will be readily available to advise on trial related medical questions or problems.

The occurrence of AEs must be sought by non-directive questioning of the participant at each visit during the study. AEs also may be detected when they are volunteered by the participant during or between visits or through physical examination findings, laboratory test findings, or other assessments.

Adverse events must be recorded under the signs, symptoms, or diagnosis associated with them, accompanied by the following information (as far as possible) (if the event is serious refer to Section 10.1.2 Serious adverse events):

1. The severity grade:

- mild: usually transient in nature and not interfering with normal activities
- moderate: sufficiently discomforting to interfere with normal activities
- severe: prevents normal activities

2. Its relationship to the study treatment. If the event is due to lack of efficacy or progression of underlying illness (i.e. progression of the study indication) the assessment of causality will usually be ‘Not suspected.’ The rationale for this guidance is that the symptoms of a lack of efficacy or progression of underlying illness are not caused by the trial drug, they happen in spite of its administration and/or both lack of efficacy and progression of underlying disease can only be evaluated meaningfully by an analysis of cohorts, not on a single participant

3. Its duration (start and end dates) or if the event is ongoing, an outcome of not recovered/not resolved must be reported

4. Whether it constitutes a SAE (see Section 10.1.2 Serious adverse events for definition of SAE) and which seriousness criteria have been met

5. Action taken regarding with study treatment.

All adverse events must be treated appropriately. Treatment may include one or more of the following:

- Dose not changed
- Dose Reduced/increased
- Drug interrupted/withdrawn

6. Its outcome

Conditions that were already present at the time of informed consent should be recorded in medical history of the participant.

AEs (including lab abnormalities that constitute AEs) should be described using a diagnosis whenever possible, rather than individual underlying signs and symptoms.

Once an AE is detected, it must be followed until its resolution or until it is judged to be permanent (e.g. continuing at the end of the study), and assessment must be made at each visit (or more frequently, if necessary) of any changes in severity, the suspected relationship to the interventions required to treat it, and the outcome.

Information about known adverse reactions for the investigational drug can be found in the IB.

Abnormal laboratory values or test results constitute adverse events only if they fulfill at least one of the following criteria:

- they induce clinical signs or symptoms
- they are considered clinically significant
- they require therapy

Clinically significant abnormal laboratory values or test results must be identified through a review of values outside of normal ranges/clinically notable ranges, significant changes from baseline or the previous visit, or values which are considered to be non-typical in participant with the underlying condition or disease.

10.1.2 Serious adverse events

A SAE is defined as any adverse event [appearance of (or worsening of any preexisting)] undesirable sign(s), symptom(s), or medical conditions(s) which meets any one of the following criteria:

- fatal
- life-threatening

Life-threatening in the context of a SAE refers to a reaction in which the participant was at risk of death at the time of the reaction; it does not refer to a reaction that might have caused death if it were more severe (please refer to the ICH-GCP Guidelines).

- results in persistent or significant disability/incapacity
- constitutes a congenital anomaly/birth defect
- requires inpatient hospitalization or prolongation of existing hospitalization, unless hospitalization is for:
  - social reasons and respite care in the absence of any deterioration in the participant's general condition
  - treatment on an emergency outpatient basis for an event not fulfilling any of the definitions of a SAE given above and not resulting in hospital admission
  - is medically significant, e.g. defined as an event that jeopardizes the participant or may require medical or surgical intervention to prevent one of the outcomes listed above

Medical and scientific judgment should be exercised in deciding whether other situations should be considered serious reactions, such as important medical events that might not be immediately life threatening or result in death or hospitalization but might jeopardize the participant or might require intervention to prevent one of the other outcomes listed above. Such events should be considered as "medically significant." Examples of such events are intensive treatment in an emergency room or at home for allergic bronchospasm, blood dyscrasias, or convulsions that do not result in hospitalization or development of dependency or abuse (please refer to the ICH/ GCP Guidelines).

All new malignant neoplasms will be assessed as serious under "medically significant" if other seriousness criteria are not met.

Any suspected transmission via a medicinal product of an infectious agent is also considered a serious adverse reaction.

All reports of intentional misuse and abuse of the product are also considered serious adverse event irrespective if a clinical event has occurred.

10.1.3 SAE reporting

To ensure participant safety, every SAE, regardless of causality, occurring after the participant has provided informed consent and until the study completion visit or 30 days after end of trial for participants who stopped study early must be reported to Vaxess safety within 24 hours of learning of its occurrence. Detailed instructions regarding the submission process and requirements are to be found in the investigator folder provided to each site.

All follow-up information for the SAE including information on complications, progression of the initial SAE and recurrent episodes must be reported as follow-up to the original episode within 24 hours of the investigator receiving the follow-up information. An SAE occurring at a different time interval or otherwise considered completely unrelated to a previously reported one must be reported separately as a new event.

If the SAE is not previously documented in the IB (new occurrence) and is thought to be related to the study treatment, a safety associate may urgently require further information from the investigator for health authority reporting. Vaxess may need to issue an Investigator Notification (IN) to inform all investigators involved in any study with the same study treatment that this SAE has been reported.

Suspected Unexpected Serious Adverse Reactions (SUSARs) will be collected and reported to the competent authorities and relevant ethics committees in accordance with EU Guidance 2011/C 172/01 or as per national regulatory requirements in participating countries.

Screen failure participants
SAEs occurring after the participant has provided informed consent until the time the participant is deemed a Screen Failure must be reported to Vaxess or designate.

Randomized or treated participants
SAEs collected between time participant signs ICF until end of study (participant has completed study or stopped study early).

Any SAEs experienced after the end of study should only be reported to Vaxess or designate Safety if the investigator suspects a causal relationship to study treatment.

10.1.4 Pregnancy reporting

Pregnancies

If a female trial participant becomes pregnant, the trial participant must be asked to read and sign pregnancy consent form to allow the Study Doctor ask about her pregnancy. To ensure participant safety, each pregnancy occurring after signing the informed consent must be reported to Vaxess or designee within 24 hours of learning of its occurrence. The pregnancy should be followed up to determine outcome, including spontaneous or voluntary termination, details of the birth, and the presence or absence of any birth defects, congenital abnormalities, or maternal and/or newborn complications.

Pregnancy should be recorded and reported by the investigator to Vaxess or appropriate designee. Pregnancy follow-up should be recorded on the same form and should include an assessment of the possible relationship to the any pregnancy outcome. Any SAE experienced during pregnancy must be reported.

Pregnancy outcomes should be collected for the female partners of any males who took study treatment in this study. Consent to report information regarding these pregnancy outcomes should be obtained from the mother. Pregnancies should be followed up until birth.

10.1.5 Reporting of study treatment errors including misuse/abuse

Medication errors are unintentional errors in the prescribing, dispensing, administration or monitoring of a medicine while under the control of a healthcare professional, participant or consumer

Misuse refers to situations where the medicinal product is intentionally and inappropriately used not in accordance with the protocol.

Abuse corresponds to the persistent or sporadic, intentional excessive use of a medicinal product, which is accompanied by harmful physical or psychological effects.

Study treatment errors and uses outside of what is foreseen in the protocol will be recorded on the appropriate eCRF irrespective of whether or not associated with an AE/SAE and reported to Safety only if associated with an SAE. Misuse or abuse will be collected and reported in the safety database irrespective of it being associated with an AE/SAE within 24 hours of investigator's awareness.

Table 8: Guidance for capturing the study treatment errors including misuse/abuse

Treatment error type	Document in Dosing eCRF (Yes/No)	Document in AE eCRF	Complete SAE form
Unintentional study treatment error	Yes	Only if associated with an AE	Only if associated with an SAE
Misuse/Abuse	Yes	Yes	Yes, even if not associated with a SAE

For more information on AE and SAE definition and reporting requirements, please see the respective sections.

10.2 Additional Safety Monitoring

SERUM	Mild: Grade 1	Moderate: Grade 2	Severe: Grade 3	Potentially Life Threatening: Grade 4
ALT, AST (liver function tests) increase by a factor	1.1-2.5 x ULN	2.6-5.0 x ULN	5.1-10 x ULN	>10 x ULN
Cr mg/dL	1.5-1.7	1.8-2.0	2.1-2.5	>2.5
Blood Urea Nitrogen- BUN mg/dL	23-26	27-31	>31	Dialysis

- The laboratory values serve as a guide and are dependent on the institutional reference ranges.

11 DATA COLLECTION AND DATABASE MANAGEMENT

11.1 Data collection

All data should be recorded, handled, and stored in a way that allows its accurate reporting, interpretation, and verification.

Designated investigator staff will enter the data required by the protocol into the eCRFs. The eCRFs have been built using fully validated secure web enabled software that conforms to 21 CFR Part 11 requirements, Investigator site staff will not be given access to the EDC system until they have been trained. Automatic validation programs check for data discrepancies in the eCRFs, allow modification and/or verification of the entered data by the investigator staff.

The investigator/designee is responsible for assuring that the data (recorded on eCRFs) (entered into eCRF) is complete, accurate, and that entry and updates are performed in a timely manner. The Investigator must certify that the data entered are complete and accurate.

After final database lock, the investigator will receive copies of the participant data for archiving at the investigational site.

All data should be recorded, handled, and stored in a way that allows its accurate reporting, interpretation, and verification.

11.2 Database management and quality control

Vaxess personnel (or designated CRO) will review the data entered by investigational staff for completeness and accuracy. Electronic data queries stating the nature of the problem and requesting clarification will be created for discrepancies and missing values and sent to the investigational site as Data Clarification Forms (DCFs) via email. Designated investigator site staff are required to respond promptly to queries and to make any necessary changes to the data.

Concomitant treatments and prior medications entered into the database will be coded using the WHO Drug Reference List, which employs the Anatomical Therapeutic Chemical classification system. Medical history/current medical conditions and adverse events will be coded using the MedDRA terminology.

At the conclusion of a non-IRT study, the occurrence of any emergency code breaks will be determined after return of all code break reports and unused supplies to Vaxess or designate.

Once all the necessary actions have been completed and the database has been declared to be complete and accurate, it will be locked, and the treatment codes will be unblinded and made available for data analysis/moved to restricted area to be accessed by independent programmer and statistician. Any changes to the database after that time can only be made after written agreement by Vaxess development management.

11.3 Site monitoring

Before study initiation, at a site initiation visit, a Vaxess delegated Contract Research Organization (CRO) representative will review the protocol and data capture requirements (i.e. eCRFs) with the investigators and their staff. During the study, Vaxess or designate employs several methods of ensuring protocol and GCP compliance and the quality/integrity of the sites' data. The field monitor will visit the site to check the completeness of participant records, the accuracy of data capture / data entry, the adherence to the protocol and to GCP, the progress of enrollment, and to ensure that study treatment is being stored, dispensed, and accounted for according to specifications. Key study personnel must be available to assist the field monitor during these visits. Continuous remote monitoring of each site's data may be performed by a centralized Vaxess/delegated CRO/Clinical Research Associate (CRA) organization. Additionally, a central analytics organization may analyze data and identify risks and trends for site operational parameters and provide reports to Vaxess clinical team to assist with trial oversight.

The investigator must maintain source documents for each participant in the study, consisting of case and visit notes (hospital or clinic medical records) containing demographic and medical information, laboratory data, electrocardiograms, and the results of any other tests or assessments. All information on eCRFs must be traceable to these source documents in the participant's file. Data not requiring a separate written record will be defined before study start

and will be recorded directly on the eCRFs. The investigator must also keep the original ICF signed by the participant (a signed copy is given to the participant).

The investigator must give the monitor access to all relevant source documents to confirm their consistency with the data capture and/or data entry. Vaxess or designate' s monitoring standards require full verification for the presence of informed consent, adherence to the inclusion/exclusion criteria, documentation of SAEs, and of data that will be used for all primary variables. Additional checks of the consistency of the source data with the eCRFs are performed according to the study-specific monitoring plan. No information in source documents about the identity of the participants will be disclosed.

12 DATA ANALYSIS AND STATISTICAL METHODS

Any data analysis carried out independently by the investigator should be submitted to Vaxess before publication or presentation.

12.1 Analysis sets

For all analysis sets, participants will be analyzed according to the study treatment(s) received.

The safety analysis set will include all participants who received any study treatment.

The immunogenicity analysis set will include all participants with at least one available valid (i.e. not flagged for exclusion) immunogenicity concentration measurement, who received any study drug and with no protocol deviations that impact immunogenicity data.

12.2 Participant demographics and other baseline characteristics

Demographic and other baseline data will be listed and summarized descriptively by ethnicity and treatment group for the Safety set.

Categorical data will be presented as frequencies and percentages. For continuous data, mean, standard deviation (SD), median, minimum, and maximum will be presented.

Relevant medical histories and current medical conditions at baseline will be summarized jointly by system organ class, preferred term, ethnicity and by treatment group.

12.3 Treatments

The Safety set will be used for the analyses below. Categorical data will be summarized as frequencies and percentages. For continuous data, mean, standard deviation, median, minimum, and maximum will be presented.

Concomitant medications and significant non-drug therapies prior to and after the start of the study treatment will be listed by treatment group, ethnicity, and participant. Summaries will be presented according to the Anatomical Therapeutic Chemical (ATC) classification system by ethnicity and treatment group.

12.4 Analysis of the primary endpoint(s)

The primary objective of this study is to determine the safety, reactogenicity and tolerability of after single MIMIX MAP skin immunization in healthy adults. All safety summaries will be performed on the safety analysis set by ethnicity.

12.4.1 Definition of primary endpoint(s)

All safety data, including vital signs, laboratory parameters, AE, local and systemic reactogenicity events, physical exams are considered primary endpoints.

12.4.2 Statistical model, hypothesis, and method of analysis

For all safety analyses, the safety data set will be used. All listings will be presented by treatment group ethnicity, participant and visit/time (if applicable). Summaries will be presented by treatment group, ethnicity and visit/time (if applicable), with placebo data being pooled.

Adverse events

All information obtained on AEs will be displayed by treatment group, ethnicity, and participant.

The number (and percentage) of participants with treatment-emergent AEs (events started on or after the dose of study medication or events present prior to start of treatment but increased in severity on or after the dose of study medication based on preferred term) will be summarized in the following ways:

- by treatment, ethnicity, primary system organ class and preferred term.
- by treatment, ethnicity, primary system organ class, preferred term and maximum severity

Separate summaries will be provided for study medication related AEs, death, SAEs, other significant AEs leading to discontinuation.

A participant with multiple AEs within a primary system organ class is only counted once towards the total of the primary system organ class.

Vital signs

All vital signs data will be listed by treatment group, ethnicity, participant, and visit/time and if ranges are available, abnormalities will be flagged. Summary statistics will be provided by treatment group, ethnicity, and visit/time.

Clinical laboratory evaluations

All laboratory data will be listed by treatment group, ethnicity, participant, and visit/time and if normal ranges are available abnormalities will be flagged. Summary statistics will be provided by treatment group, ethnicity, and visit/time. Shift tables using the low/normal/high/ (low and high) classification will be used to compare baseline to the worst on-treatment value.

Other safety evaluations

All listings deemed relevant, including reactogenicity, physical examination, will be presented by treatment group, ethnicity, participant and visit. Summary statistics deemed relevant will be provided by treatment group, ethnicity and visit.

12.5 Analysis of secondary endpoints

12.5.1 Efficacy and/or immunogenicity endpoint(s)

All immunogenicity data will be listed by treatment group and timepoint.

12.5.2 Safety endpoints

Not applicable.

12.5.3 Immunogenicity

Although clinical efficacy trials of new influenza vaccines may be performed in late-stage development or post-licensure, FDA licensure can be achieved based upon antigen specific HAI titers. The primary immunogenicity analyses will assess HAI GMTs, GMT mean fold rise, seroconversion rates (defined as a fourfold rise in HAI titers) and seroprotection status (defined as an HAI titer of ≥1:40), by HAI.

HAI antibodies in serum will be determined at specific time points for all participants, including the placebo group and all immunogenicity results will be listed by treatment group, ethnicity, participant and visit/time. Summary statistics may be provided as appropriate in case of high incidence of positive participants/samples.

Sera analyzed for determination of HAI titers will be evaluated as follows:

- Calculation of the geometric mean of pre- and post-vaccination HAI titers for each treatment group.
- Change from pre- to post-vaccination in geometric mean HAI titers for each treatment group.
- Seroconversion rate defined as percentage of participants with either a pre-vaccination HAI titer <1:10 and post vaccination HAI titer of ≥ 1:40 or a pre-vaccination HAI titer of ≥ 1:10 and a minimum four-fold rise in post vaccination HAI antibody titer at each timepoint.
- Seroprotection rate defined as the percentage of participants with a HAI titer ≥ 1:40 at each timepoint for the vaccine H1 strain

Incidence of positive participants and correlation with selected AEs and clinical outcomes may be explored as appropriate in case of high incidence.

12.6 Analysis of exploratory endpoints

Exploratory analyses will include evaluations of seroprotection status (defined as an HAI titer of ≥1:40), H1 virus microneutralization titers, GMT of H1 HA specific IgG, total IgE titers, GMT of silk fibroin specific IgG and HAI titer against against a drifted H1 strain. These endpoints will be assessed on (vaccination day), and on Days 29 (±1), and 180 (± 5).

Sera analyzed for determination of HAI, serum anti-H1 HA IgG, virus microneutralization titers, total serum IgE and anti-silk fibroin by ELISA results will be evaluated as follows:

- Calculation of the geometric mean of pre- and post-vaccination HAI titers for each treatment group for a drifted H1 strain.
- Calculation of the geometric mean of pre- and post-vaccination anti-H1 IgG serum antibody titers for each treatment group for the vaccine H1 strain.

- Change from pre- to post-vaccination in geometric mean anti-H1 IgG for each treatment group for the vaccine H1 strain.
- Calculation of the geometric mean of pre- and post-vaccination anti-silk fibroin IgG serum antibody titers for each treatment group.
- Change from pre- to post-vaccination in geometric mean anti-silk fibroin IgG titers.
- Calculation of the geometric mean of pre- and post-vaccination total serum IgE titers
- Change from pre-to post-vaccination in geometric mean total IgE titers

12.7 Interim analyses

An interim analysis will be conducted once all enrolled participants have completed Day 57 assessments or End of Study Visits or have withdrawn or are lost to follow-up.

12.8 Sample size calculation

The primary objective of this FIH study is to assess the safety, reactogenicity and tolerability of VX-103. A sample size of 30 participants on active treatment (with additional 15 placebo participants) is consistent with historical clinical practice in similar studies.

13 ETHICAL CONSIDERATIONS AND ADMINISTRATIVE PROCEDURES

13.1 Regulatory and ethical compliance

This clinical study was designed and shall be implemented, executed and reported in accordance with the ICH Harmonized Tripartite Guidelines for Good Clinical Practice, with applicable local regulations (including European Directive 2001/20/EC, US CFR 21), and with the ethical principles laid down in the Declaration of Helsinki.

13.2 Responsibilities of the investigator and IRB/IEC

Before initiating a trial, the investigator/institution must obtain approval/favorable opinion from the Institutional Review Board/Independent Ethics Committee (IRB/IEC) for the trial protocol, written ICF, consent form updates, participant recruitment procedures (e.g., advertisements) and any other written information to be provided to participants. Prior to study start, the investigator is required to sign a protocol signature page confirming his/her agreement to conduct the study in accordance with these documents and all of the instructions and procedures found in this protocol and to give access to all relevant data and records to Vaxess or designate' s monitors, auditors, Quality Assurance representatives, designated agents of Vaxess, IRBs/IECs, and regulatory authorities as required. If an inspection of the clinical site is requested by a regulatory authority, the investigator must inform Vaxess and designate immediately that this request has been made.

13.3 Publication of study protocol and results

This study only involves healthy volunteers and as such Vaxess will register the protocol as required to databases specified by local regulations. After study completion and finalization of the study report, results of this trial may be submitted for publication (e.g. peer-reviewed journal) or registered to databases where required by local regulations.

13.4 Quality Control and Quality Assurance

Vaxess and/or designate maintains a Quality Management System (QMS) that includes all activities involved in quality assurance and quality control, to ensure compliance with written Standard Operating Procedures as well as applicable global/local GCP regulations and ICH Guidelines.

Audits of investigator sites, vendors, and Vaxess or designate may be performed by auditors, independent from those involved in conducting, monitoring or performing quality control of the clinical trial. The clinical audit process uses a knowledge/risk-based approach.

Audits may be conducted to assess GCP compliance with global and local regulatory requirements, protocols and internal SOPs, and are performed according to written Vaxess and/or designate' s processes.

14 PROTOCOL ADHERENCE

This protocol defines the study objectives, the study procedures and the data to be collected on study participants. Additional assessments required to ensure safety of participants should be administered as deemed necessary on a case-by-case basis. Under no circumstances including incidental collection is an investigator allowed to collect additional data or conduct any additional procedures for any purpose involving any investigational drugs under the protocol, other than the purpose of the study. If despite this interdiction prohibition, data, information, observation would be incidentally collected, the investigator shall immediately disclose it to Vaxess or designate and not use it for any purpose other than the study, except for the appropriate monitoring on study participants.

Investigators ascertain they will apply due diligence to avoid protocol deviations. If an investigator feels a protocol deviation would improve the conduct of the study this must be considered a protocol amendment, and unless such an amendment is agreed upon by Vaxess or designate and approved by the IRB/IEC and Health Authorities, where required, it cannot be implemented.

14.1 Protocol amendments

Any change or addition to the protocol can only be made in a written protocol amendment that must be approved by Vaxess, health authorities where required, and the IRB/IEC prior to implementation.

Only amendments that are required for participant safety may be implemented immediately provided the health authorities are subsequently notified by protocol amendment and the reviewing IRB/IEC is notified.

Notwithstanding the need for approval of formal protocol amendments, the investigator is expected to take any immediate action required for the safety of any participant included in this study, even if this action represents a deviation from the protocol. In such cases, Vaxess should be notified of this action and the IRB/IEC at the study site should be informed according to local regulations.

REFERENCES

Abdullahi, A., et al. (2014). Animal models in burn research. *Cell Mol Life Sci, 71*(17), 3241-3255. doi:10.1007/s00018-014-1612-5

Allen, J. D., et al. (2018). H3N2 influenza viruses in humans: Viral mechanisms, evolution, and evaluation. *Hum Vaccin Immunother, 14*(8), 1840-1847. doi:10.1080/21645515.2018.1462639

Belongia, E. A., et al. (2016). Variable influenza vaccine effectiveness by subtype: a systematic review and meta-analysis of test-negative design studies. *Lancet Infect Dis, 16*(8), 942-951. doi:10.1016/S1473-3099(16)00129-8

CDC. (2020). Who Needs a Flu Vaccine and When. Retrieved from https://www.cdc.gov/flu/prevent/vaccinations.htm

Darvishian, M., et al. (2014). Effectiveness of seasonal influenza vaccine in community-dwelling elderly people: a meta-analysis of test-negative design case-control studies. *Lancet Infect Dis, 14*(12), 1228-1239. doi:10.1016/S1473-3099(14)70960-0

Davis, C. W., et al. (2020). Influenza vaccine-induced human bone marrow plasma cells decline within a year after vaccination. *Science, 370*(6513), 237-241. doi:10.1126/science.aaz8432

Egunsola, O., et al. (2021). Immunogenicity and Safety of Reduced-Dose Intradermal vs Intramuscular Influenza Vaccines: A Systematic Review and Meta-analysis. *JAMA Netw Open, 4*(2), e2035693. doi:10.1001/jamanetworkopen.2020.35693

FDA. (2007). *Toxicity Grading Scale for Healthy Adult and Adolescent Volunteers Enrolled in Preventive Vaccine Clinical Trials* Retrieved from https://www.govinfo.gov/app/details/FR-2007-09-27/E7-19155

Kenney, R. T., et al. (2004). Dose sparing with intradermal injection of influenza vaccine. *N Engl J Med, 351*(22), 2295-2301. doi:10.1056/NEJMoa043540

La Montagne, J. R., et al. (2004). Intradermal influenza vaccination--can less be more? *N Engl J Med, 351*(22), 2330-2332. doi:10.1056/NEJMe048314

Rondy, M., et al. (2017). Effectiveness of influenza vaccines in preventing severe influenza illness among adults: A systematic review and meta-analysis of test-negative design case-control studies. *J Infect, 75*(5), 381-394. doi:10.1016/j.jinf.2017.09.010

Song, J. Y., et al. (2013). Long-term immunogenicity of the influenza vaccine at reduced intradermal and full intramuscular doses among healthy young adults. *Clin Exp Vaccine Res, 2*(2), 115-119. doi:10.7774/cevr.2013.2.2.115

Stinson, J. A., et al. (2021). Enhancing influenza vaccine immunogenicity and efficacy through infection mimicry using silk microneedles. *Vaccine, 39*(38), 5410-5421. doi:10.1016/j.vaccine.2021.07.064

APPENDICES

Appendix 1: Local Tolerability Scoring

Local tolerability scoring is adapted from the 2007 Food and Drug Association (FDA) Healthy Adult and Adolescent Volunteers Enrolled in Preventive Vaccine Clinical Trials (FDA, 2007) for assessment of Vaxess's 1 cm square MAP .

Local Reaction to Product	Grade 0	Mild (Grade 1)	Moderate (Grade 2)	Severe (Grade 3)	Potentially Life Threatening (Grade 4)
Pain	Pain is absent	Does not interfere with activity	Repeated use of non-narcotic pain reliever > 24 hours or interferes with activity	Any use of narcotic pain reliever or prevents daily activity	Emergency room (ER) visit or hospitalization
Tenderness	No discomfort to touch	Mild discomfort to touch	Discomfort with movement	Significant discomfort at rest	ER visit or hospitalization
Erythema/Redness*	<1 cm	1– 5 cm	5.1 – 10 cm	> 10 cm	Necrosis or exfoliative dermatitis
Induration/ Swelling **	<1 cm	1 – 5 cm and does not interfere with activity	5.1 – 10 cm or interferes with activity	> 10 cm or prevents daily activity	Necrosis

*In addition to grading the measured local reaction at the greatest single diameter, the measurement should be recorded as a continuous variable.
** Induration/Swelling should be evaluated and graded using the functional scale as well as the actual measurement.

Appendix 2: Other Local Tolerability Scoring

Bruising Extent
The treatment area will be assessed for bruising
Grade Description:
0 NONE No visible bruising around treatment area
1 MILD 0 to 2cm bruising around treatment area
2 MODERATE 2 to 5 cm bruising around treatment area
3 SEVERE Greater than 5 cm bruising around treatment area

Itching
Participants will be asked the degree of itching they are experiencing on average in the last 24 hours:
Grade Description:
0 NONE
1 MILD
2 MODERATE
3 SEVERE

Skin Flaking
Participants will be asked the amount of skin flaking of the treatment area they are experiencing:
Grade Description:
0 NONE
1 MILD
2 MODERATE
3 SEVERE

Application Site Visibility
Pigmented (melanin) coloration of the treatment area will be assessed following loss of erythema:
Grade Description:
0 Pigmented area <1 cm
1 MILD Pigmented area ≥1 cm and slightly visible
2 MODERATE Pigmented area ≥1 cm, noticeable but not clearly defined
3 SEVERE Pigmented area ≥1 cm and very clearly defined

Appendix 3: Systemic Toxicity Grading Scales

Toxicity Grading Scales are derived from 2007 Food and Drug Association (FDA) Healthy Adult and Adolescent Volunteers Enrolled in Preventive Vaccine Clinical Trials (FDA, 2007).

Clinical Abnormalities:

Vital Signs *	Mild (Grade 1)	Moderate (Grade 2)	Severe (Grade 3)	Potentially Life Threatening (Grade 4)
Fever (°C) **<br>(°F) **	38.0 – 38.4<br>100.4 – 101.1	38.5 – 38.9<br>101.2 – 102.0	39.0 – 40<br>102.1 – 104	> 40<br>> 104
Tachycardia - beats per minute	101 – 115	116 – 130	> 130	ER visit or hospitalization for arrhythmia
Bradycardia - beats per minute***	50 – 54	45 – 49	< 45	ER visit or hospitalization for arrhythmia
Hypertension (systolic) - mm Hg	141 – 150	151 – 155	> 155	ER visit or hospitalization for malignant hypertension
Hypertension (diastolic) - mm Hg	91 – 95	96 – 100	> 100	ER visit or hospitalization for malignant hypertension
Hypotension (systolic) – mm Hg	85 – 89	80 – 84	< 80	ER visit or hospitalization for hypotensive shock
Respiratory Rate – breaths per minute	17 – 20	21 – 25	> 25	Intubation

* Subject should be at rest for all vital sign measurements.
** Oral temperature; no recent hot or cold beverages or smoking.
*** When resting heart rate is between 60 – 100 beats per minute. Use clinical judgement when characterizingbradycardia among some healthy subject populations, for example, conditioned athletes.

Systemic (General)	Mild (Grade 1)	Moderate (Grade 2)	Severe (Grade 3)	Potentially Life Threatening (Grade 4)
Nausea/vomiting	No interference with activity or 1 – 2 episodes/24 hours	Some interference with activity or > 2 episodes/24 hours	Prevents daily activity, requires outpatient IV hydration	ER visit or hospitalization for hypotensive shock
Diarrhea	2 – 3 loose stools or < 400 gms/24 hours	4 – 5 stools or 400 – 800 gms/24 hours	6 or more watery stools or<br>> 800gms/24 hours or requires outpatient IV hydration	ER visit or hospitalization
Headache	No interference with activity	Repeated use of non-narcotic pain reliever > 24 hours or some interference with activity	Significant; any use of narcotic pain reliever or prevents daily activity	ER visit or hospitalization

Fatigue	No interference with activity	Some interference with activity	Significant; prevents daily activity	ER visit or hospitalization
Myalgia	No interference with activity	Some interference with activity	Significant; prevents daily activity	ER visit or hospitalization

Laboratory Abnormalities (Hematology):

Hematology	Mild (Grade 1)	Moderate (Grade 2)	Severe (Grade 3)	Potentially Life Threatening (Grade 4)
Hemoglobin (Female) - gm/dL	11.0 – 12.0	9.5 – 10.9	8.0 – 9.4	< 8.0
Hemoglobin (Female) change from baseline value - gm/dL	Any decrease – 1.5	1.6 – 2.0	2.1 – 5.0	> 5.0
Hemoglobin (Male) - gm/dL	12.5 – 13.5	10.5 – 12.4	8.5 – 10.4	< 8.5
Hemoglobin (Male) change from baseline value – gm/dL	Any decrease – 1.5	1.6 – 2.0	2.1 – 5.0	> 5.0
WBC Increase - cell/mm^3	10,800 – 15,000	15,001 – 20,000	20,001 – 25, 000	> 25,000
WBC Decrease - cell/mm^3	2,500 – 3,500	1,500 – 2,499	1,000 – 1,499	< 1,000
Lymphocytes Decrease - cell/mm^3	750 – 1,000	500 – 749	250 – 499	< 250
Neutrophils Decrease - cell/mm^3	1,500 – 2,000	1,000 – 1,499	500 – 999	< 500
Eosinophils - cell/mm^3	650 – 1500	1501 – 5000	> 5000	Hypereosinophilic
Platelets Decreased - cell/mm^3	125,000 – 140,000	100,000 – 124,000	25,000 – 99,000	< 25,000
PT – increase by factor (prothrombin time)	1.0 – 1.10 x ULN*	1.11 – 1.20 x ULN	1.21 – 1.25 x ULN	> 1.25 ULN
PTT – increase by factor (partial thromboplastin time)	1.0 – 1.2 x ULN	1.21 – 1.4 x ULN	1.41 – 1.5 x ULN	> 1.5 x ULN
Fibrinogen increase - mg/dL	400 – 500	501 – 600	> 600	--
Fibrinogen decrease - mg/dL	150 – 200	125 – 149	100 – 124	< 100 or associated with gross bleeding or disseminated intravascular coagulation (DIC)

* The laboratory values provided in the tables serve as guidelines and are dependent upon institutional normal parameters. Institutional normal reference ranges should be provided to demonstrate that they are appropriate.
** "ULN" is the upper limit of the normal range.

Laboratory Abnormalities (Serum):

Serum	Mild (Grade 1)	Moderate (Grade 2)	Severe (Grade 3)	Potentially Life Threatening (Grade 4)
Sodium – Hyponatremia mEq/L	132 – 134	130 – 131	125 – 129	< 125
Sodium – Hypernatremia mEq/L	144 – 145	146 – 147	148 – 150	> 150
Potassium – Hyperkalemia mEq/L	5.1 – 5.2	5.3 – 5.4	5.5 – 5.6	> 5.6
Potassium – Hypokalemia mEq/L	3.5 – 3.6	3.3 – 3.4	3.1 – 3.2	< 3.1

Glucose – Hypoglycemia mg/dL	65 – 69	55 – 64	45 – 54	< 45
Glucose – Hyperglycemia Fasting– mg/dL<br>Random – mg/dL	100 – 110<br>110 – 125	111 – 125<br>126 – 200	>125<br>>200	Insulin requirements or hyperosmolar coma
BUN mg/dL	23 – 26	27 – 31	> 31	Requires dialysis
Cr – mg/dL	1.5 – 1.7	1.8 – 2.0	2.1 – 2.5	> 2.5 or requires dialysis
Calcium – hypocalcemia mg/dL	8.0 – 8.4	7.5 – 7.9	7.0 – 7.4	< 7.0
Calcium – hypercalcemia mg/dL	10.5 – 11.0	11.1 – 11.5	11.6 – 12.0	> 12.0
Magnesium – hypomagnesemia mg/dL	1.3 – 1.5	1.1 – 1.2	0.9 – 1.0	< 0.9
Phosphorous – hypophosphatemia mg/dL	2.3 – 2.5	2.0 – 2.2	1.6 – 1.9	< 1.6
CPK – mg/dL	1.25 – 1.5 x ULN	1.6 – 3.0 x ULN	3.1 –10 x ULN	> 10 x ULN
Albumin – Hypoalbuminemia g/dL	2.8 – 3.1	2.5 – 2.7	< 2.5	--
Total Protein – Hypoproteinemia g/dL	5.5 – 6.0	5.0 – 5.4	< 5.0	--
Alkaline phosphate – increase by factor	1.1 – 2.0 x ULN	2.1 – 3.0 x ULN	3.1 – 10 x ULN	> 10 x ULN
Liver Function Tests –ALT, AST increase by factor	1.1 – 2.5 x ULN	2.6 – 5.0 x ULN	5.1 – 10 x ULN	> 10 x ULN
Bilirubin – when accompanied by any increase in Liver Function Test increase by factor	1.1 – 1.25 x ULN	1.26 – 1.5 x ULN	1.51 – 1.75 x ULN	> 1.75 x ULN
Bilirubin – when Liver Function Test is normal; increase by factor	1.1 – 1.5 x ULN	1.6 – 2.0 x ULN	2.0 – 3.0 x ULN	> 3.0 x ULN
Cholesterol	201 – 210	211 – 225	> 226	---
Pancreatic enzymes – amylase, lipase	1.1 – 1.5 x ULN	1.6 – 2.0 x ULN	2.1 – 5.0 x ULN	> 5.0 x ULN

* The laboratory values provided in the tables serve as guidelines and are dependent upon institutional normal parameters. Institutional normal reference ranges should be provided to demonstrate that they are appropriate.
** The clinical signs or symptoms associated with laboratory abnormalities might result in characterization of the laboratory abnormalities as Potentially Life Threatening (Grade 4). For example. a low sodium value that falls within a grade 3 parameter (125-129 mE/L) should be recorded as a grade 4 hyponatremia event if the subject had a new seizure associated with the low sodium value.
*** ULN" is the upper limit of the normal range.

Laboratory Abnormalities (Urine):

Urine	Mild (Grade 1)	Moderate (Grade 2)	Severe (Grade 3)	Potentially Life Threatening (Grade 4)
Protein	Trace	1+	2+	Hospitalization or dialysis
Glucose	Trace	1+	2+	Hospitalization for hyperglycemia
Blood (microscopic) – red blood cells per high power field (rbc/hpf)	1 - 10	11 – 50	> 50 and/or gross blood	Hospitalization or packed red blood cells (PRBC) transfusion

*The laboratory values provided in the tables serve as guidelines and are dependent upon institutional normal parameters. Institutional normal reference ranges should be provided to demonstrate that they are appropriate.
